# Robustness of cancer microbiome signals over a broad range of methodological variation

**DOI:** 10.1038/s41388-024-02974-w

**Published:** 2024-02-23

**Authors:** Gregory D. Sepich-Poore, Daniel McDonald, Evguenia Kopylova, Caitlin Guccione, Qiyun Zhu, George Austin, Carolina Carpenter, Serena Fraraccio, Stephen Wandro, Tomasz Kosciolek, Stefan Janssen, Jessica L. Metcalf, Se Jin Song, Jad Kanbar, Sandrine Miller-Montgomery, Robert Heaton, Rana Mckay, Sandip Pravin Patel, Austin D. Swafford, Tal Korem, Rob Knight

**Affiliations:** 1 Department of Bioengineering, University of California San Diego, La Jolla, CA USA; 2 Department of Pediatrics, University of California San Diego, La Jolla, CA USA; 3 Clarity Genomics, Antwerp, Belgium; 4 Department of Biomedical Informatics, Columbia University Irving Medical Center, New York, NY USA; 5 Program for Mathematical Genomics, Department of Systems Biology, Columbia University Irving Medical Center, New York, NY USA; 6 Present Address: Center for Microbiome Innovation, University of California San Diego, La Jolla, CA USA; 7 Department of Animal Sciences, Colorado State University, Fort Collins, CO USA; 8 Department of Medicine, University of California San Diego, La Jolla, CA USA; 9 Department of Psychiatry, University of California San Diego, La Jolla, CA USA; 10 Moores Cancer Center, University of California San Diego Health, La Jolla, CA USA; 11 Department of Obstetrics and Gynecology, Columbia University Irving Medical Center, New York, NY USA; 12 Department of Computer Science and Engineering, University of California San Diego, La Jolla, CA USA; 13 Present Address: Micronoma, San Diego, CA USA; 14 Present Address: Feinberg School of Medicine, Northwestern University, Chicago, IL USA; 15 Present Address: School of Life Sciences, Arizona State University, Tempe, AZ USA; 16 Present Address: Malopolska Centre of Biotechnology, Jagiellonian University in Kraków, Kraków, Poland; 17 Present Address: Algorithmic Bioinformatics, Department of Biology and Chemistry, Justus Liebig University Gießen, Gießen, Germany

**Keywords:** Microbiology, Genomics, Diagnostic markers

## Abstract

In 2020, we identified cancer-specific microbial signals in The Cancer Genome Atlas (TCGA) [1]. Multiple peer-reviewed papers independently verified or extended our findings [2–12]. Given this impact, we carefully considered concerns by Gihawi et al. [13] that batch correction and database contamination with host sequences artificially created the appearance of cancer type-specific microbiomes. (1) We tested batch correction by comparing raw and Voom-SNM-corrected data per-batch, finding predictive equivalence and significantly similar features. We found consistent results with a modern microbiome-specific method (ConQuR [14]), and when restricting to taxa found in an independent, highly-decontaminated cohort. (2) Using Conterminator [15], we found low levels of human contamination in our original databases (~1% of genomes). We demonstrated that the increased detection of human reads in Gihawi et al. [13] was due to using a newer human genome reference. (3) We developed Exhaustive, a method twice as sensitive as Conterminator, to clean RefSeq. We comprehensively host-deplete TCGA with many human (pan)genome references. We repeated all analyses with this and the Gihawi et al. [13] pipeline, and found cancer type-specific microbiomes. These extensive re-analyses and updated methods validate our original conclusion that cancer type-specific microbial signatures exist in TCGA, and show they are robust to methodology.

## Introduction

As late as 2015, the tumor microbiome was considered an elusive “mirage” [16], but this notion was dispelled by the discovery of chemo-degrading bacteria in >75% of pancreatic cancers [17]. Subsequent studies annotated the functional, often immunomodulatory, impacts of these intra-pancreatic bacteria [18, 19] and fungi [20, 21], followed by characterization of microbes in non-gastrointestinal cancer types, including lung cancer [22–24] and leukemia [4, 25]. However, multi-cancer microbiome profiling was rare, and the largest attempts excluded ~85% of The Cancer Genome Atlas (TCGA) patients while lacking systematic decontamination, batch correction, cross-cancer comparisons, or blood-related analyses [26]. In 2020, we published a comprehensive analysis of microbial abundances across all 33 TCGA cancer types, with standardized methods for batch correction, in silico decontamination, and machine learning (ML) comparisons [1]. These approaches allowed us to conclude that microbial compositions were distinct between and within cancer types, and that trace amounts of their DNA were detectable in human blood samples, thereby suggesting a novel diagnostic approach [1].

Subsequently, the cancer microbiome field accelerated, including direct validation within months of our cancer type-specific conclusions in an independent, highly-decontaminated cohort [2], followed by numerous papers from independent labs around the world [3–12], and the eventual inclusion of the tumor microbiome as an emerging hallmark of cancer [27]. In 2022, we updated our methods to reflect contemporaneous host depletion and microbial read assignment approaches, allowing us to detect eukaryotes (fungi) in the same TCGA samples previously analyzed for bacteria and viruses, with matching experimental validation in an international cohort from the Weizmann Institute of Science (WIS) [28].

In 2023, Gihawi et al. [13] raised concerns about potential mishandling of human contaminants or batch effect correction artificially driving the conclusion of cancer type-specific microbiomes in TCGA. Notably, most of their methods were published after our original paper (Supplementary Fig. 1A), and although future tools do not invalidate critiques of earlier publications, it implies that the central question is whether the *conclusions* are correct, not whether a non-contemporaneous tool should have been applied. Herein, we first perform extensive re-analyses of the originally published data to address claims of data analysis errors, finding instead that their observations resulted from using new methods and human genome references. As these tools were not available at the time of the original analysis, we also re-analyze TCGA data from scratch using new methods, and demonstrate that our conclusions remain correct even with state-of-the-art resources (Supplementary Text 1.1–1.2).

The claims made by Gihawi et al. [13] depend on interacting factors. To help readers find individual claims, we provide a roadmap to our response and the locations of the specific supporting evidence in Table S1 and Table S2. These tables outline section-by-section responses to the claims made (Table S1), and a summary of the analyses performed with their associated figures (Table S2). We also provide a summary of analyses on the originally published data versus updated methods (Supplementary Text 1.1), and a timeline to contextualize this response (Supplementary Fig. 1A, B; Supplementary Text 1.2).

## Results

### Voom-SNM batch correction did not introduce systematic bias in the original data

Gihawi et al. claim that “errors in the [Voom-SNM] transformation of the raw data created an artificial signature […] tagging each tumor type with a distinct signal that the machine learning programs then used to create an apparently accurate classifier” [13]. However, in the original paper’s methods, we stated the Voom-SNM (VSNM) normalization was not provided with or otherwise exposed to cancer type information (Supplementary Text 1.3).

**Fig. 1:**
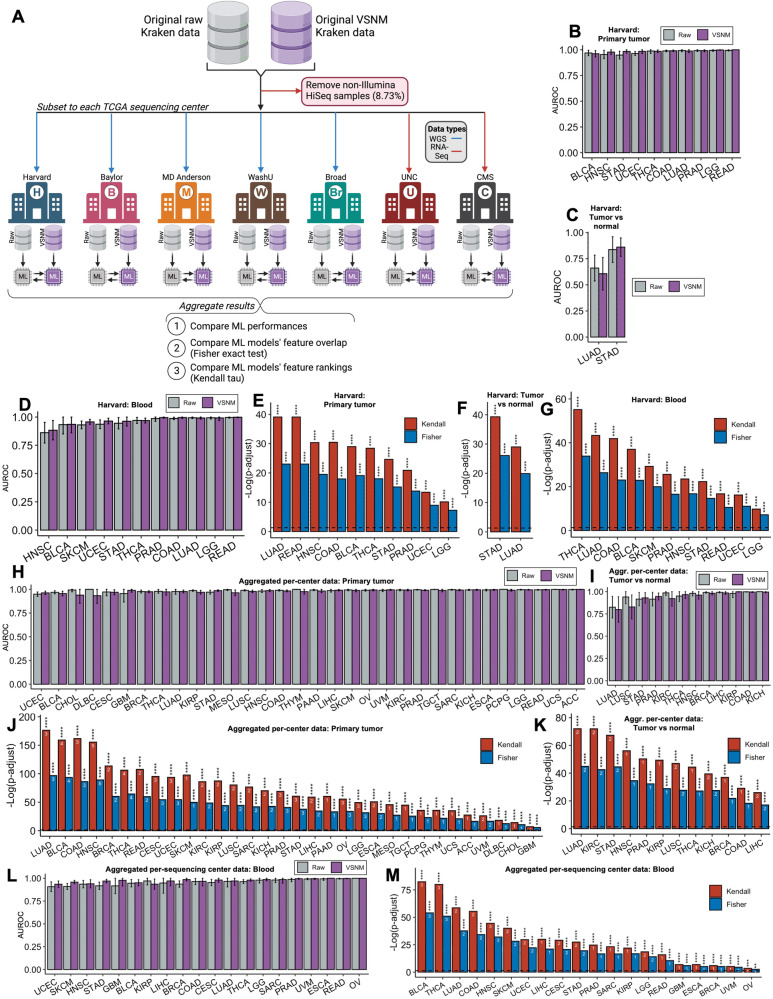
Comparing raw and Voom-SNM (VSNM) data within batches does not reveal a systematic bias from batch correction. **A** Data splitting strategy for comparing the originally published raw and VSNM data using ML performances and ML model feature similarities. Raw-versus-VSNM AUROCs from comparing cancer types using (**B**) primary tumors, (**C**) tumor versus normal tissues, and (**D**) blood samples in Harvard Medical School. Fisher exact test (blue) and Kendall tau correlation (red) *p* values from comparing raw-versus-VSNM model feature similarities when predicting cancer type among (**E**) primary tumors, (**F**) tumor versus normal tissues, and (**G**) blood samples in Harvard Medical School. Aggregated AUROC data across all per-batch (**H**) primary tumor and (**I**) tumor versus normal comparisons. Aggregated and combined *p*-values from per-batch Fisher exact tests (blue) and Kendall tau correlations (red) across all per-batch (**J**) primary tumor and (**K**) tumor versus normal comparisons. Inset white numbers denote the number of batches (i.e., sequencing centers) from which data derived for each particular cancer type. **L** Aggregated AUROC data across all per-batch blood sample comparisons. **M** Aggregated and combined *p*-values from per-batch Fisher exact tests (blue) and Kendall tau correlations (red) across all per-batch blood sample comparisons. Inset white numbers denote the number of batches (i.e., sequencing centers) from which data derived for each particular cancer type. **B**–**D**, **H**–**I**, **L** Error bars denote 99% confidence intervals. **E**–**G**, **J**, **K**, **M** *P* values adjusted among cancer types using Benjamini-Hochberg correction. When *p* values were combined across multiple batches, Fisher’s method was used on the raw per-batch *p*-values, followed by Benjamini-Hochberg correction across cancer types. Logarithms are base 10. See Supplementary Fig. 1C for list of TCGA cancer type abbreviations.

A systematic way to evaluate whether batch correction artificially drove cancer type-specific differences is to rerun the ML in every individual batch using the originally published raw and VSNM data (Fig. 1A). Artifactual signal is unlikely if per-batch VSNM results are similar to those produced from raw data, and equivalent performances would provide evidence against artificial bias. Further support for lack of artifactual signal could be obtained if the two types of per-batch ML models independently choose similar features. We thus directly compared VSNM-versus-raw, per-batch ML models’ features, both their binary overlap (i.e., Fisher’s exact test on a 2 × 2 contingency table)—since most ML models used only 10–20% of the total genera—and correlation of the models’ relative rankings of genera using feature importances (i.e., Kendall’s tau correlation) (Fig. 1A). Finding significantly similar, and similarly ranked, feature lists among all per-batch analyses, in conjunction with equivalent ML performances, would argue that systematic bias was not introduced.

We proceeded to subset the originally published raw and VSNM data to an individual sequencing platform accounting for 91.27% of samples (Illumina HiSeq), individual data types (WGS or RNA-Seq), and seven sequencing centers that focused on single data types with sufficient samples: Harvard (WGS), Baylor (WGS), MD Anderson (WGS), Washington University (WashU; WGS), the Broad Institute (WGS), University of North Carolina (UNC, RNA-Seq), and Canada’s Michael Smith Genome Sciences Centre (CMS, RNA-Seq) (Fig. 1A, Supplementary Text 1.4). We then compared areas under the receiver operating characteristic (AUROC) and precision recall (AUPR) curves. AUROC evaluates recall (i.e., true positive rate) as a function of the false positive rate. AUPR evaluates precision (i.e., one minus the false discovery rate) as a function of recall, and is not affected by true negatives. The two measures therefore provide different insights into classifier accuracy [29]. Importantly, every per-batch comparison yielded equivalent AUROCs for cancer type predictions (Fig. 1B–D; Supplementary Fig. 2E, H, K, N, Q, T; Supplementary Fig. 2B, E, H, K, O, R, U, X). AUPRs were also similar among batches, and, in a few cases (e.g., UNC primary tumor; Supplementary Fig. 2S) the raw data models outperformed the VSNM data models (Supplementary Fig. 2D, F, I, L, O, S, U; Supplementary Fig. 3A, C, F, I, L, N, P, S, V, Y). Aggregated AUROC and AUPR performances provided similar conclusions (Fig. 1H, I, L; Supplementary Fig. 2A–C). Every evaluation of per-batch model features showed significant overlap and significantly similar feature rankings (Fig. 1E–G; Supplementary Fig. 2G, J, M, P, R, V; Supplementary Fig. 3D, G, J, M, Q, T, W, Z), which increased in significance after aggregating data from each center (because Fisher’s method combines *p*-values through multiplication; Fig. 1J, K, M). We also confirmed that both data types equivalently responded to negative control analyses in which samples or labels were permuted (Supplementary Text 1.5, Supplementary Figs. 4, 5). Thus, we conclude that VSNM did not systematically bias the data: (i) equivalent ML model performances and significantly similar feature rankings result from the raw data; (ii) we observe equivalent negative control responses.

### Voom-SNM and ConQuR provide equivalent conclusions to uncorrected data

**Fig. 2:**
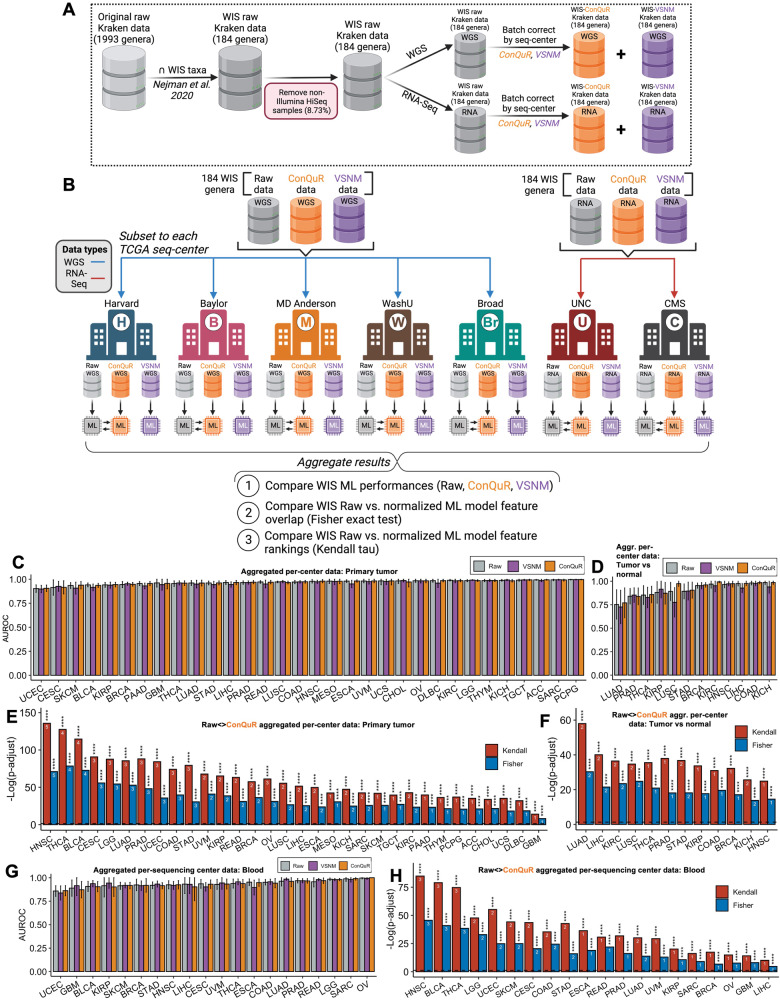
Application of a microbiome-specific batch correction tool (ConQuR) and restricting the features to WIS-overlapping genera does not change the original manuscript’s conclusions. **A** WIS-overlapping data was generated by intersecting decontaminated bacterial genera from Nejman et al. [2], followed by subsetting to Illumina HiSeq samples and WGS or RNA-Seq groups. Within each WGS or RNA-Seq group, ConQuR and VSNM were applied to correct for sequencing center bias. **B** Data splitting strategy to accommodate separate WGS and RNA-Seq datasets due to ConQuR limitations. Downstream goals were to compare ML performances among the raw, ConQuR, and VSNM data types, as well as to compare the model feature similarities between the raw and normalized data. Aggregated AUROC data across all per-batch (**C**) primary tumor and (**D**) tumor versus normal comparisons. Aggregated and combined *p*-values from ConQuR-versus-raw, per-batch Fisher exact tests (blue) and Kendall tau correlations (red) across all per-batch (**E**) primary tumor and (**F**) tumor versus normal comparisons. Inset white numbers denote the number of batches (i.e., sequencing centers) from which data derived for each particular cancer type. **G** Aggregated AUROC data across all per-batch blood sample comparisons. **H** Aggregated and combined *p* values from ConQuR-versus-raw, per-batch Fisher exact tests (blue) and Kendall tau correlations (red) across all per-batch blood sample comparisons. Inset white numbers denote the number of batches (i.e., sequencing centers) from which data derived for each particular cancer type. **C**, **D**, **G** Error bars denote 99% confidence intervals. **E**, **F**, **H** *P* values were combined across multiple batches using Fisher’s method on the raw per-batch *p* values, followed by Benjamini-Hochberg correction across cancer types. Logarithms are base 10. See Supplementary Fig. 1C for list of TCGA cancer type abbreviations.

VSNM was not designed for batch correction of microbiome data. Accordingly, we tested ConQuR [14], a newer (2022) tool developed specifically for batch correction of microbiome data. Because the critiquing authors previously cited WIS-overlapping taxa to justify their own work [30] and a cancer microbiome patent application [31]—demonstrating their acceptance of these taxa—we next limited re-analyses of our original paper’s data to the cited 184 WIS-overlapping, decontaminated, bacterial genera [2] (Fig. 2A) to test whether the same conclusions could be obtained.

We performed direct ML comparisons of VSNM, ConQuR, and raw data subsets using the same data splitting strategy (Fig. 2B). Per-batch ML of the WIS-overlapping raw data demonstrated cancer type-specificity (Supplementary Text 1.6). Voom-SNM and ConQuR were used to correct for sequencing center biases within WGS and RNA-Seq sample groups (Methods). Importantly, both ConQuR and VSNM provided equivalent reductions in sequencing center effect sizes down to ≤2.7% variance using principal variance components analyses while increasing effect sizes of cancer type up to 16.2% variance (Supplementary Fig. 6A, B). Beyond ConQuR, we also tested another recently introduced microbiome-specific batch correction tool, MMUPHin [32]. We decided not to use PLSDA-batch [33] because its output is not compatible with standard metagenomics workflows. To our knowledge, these are the only three microbiome-specific batch correction tools applicable to TCGA that exist. MMUPHin performed substantially worse than ConQuR or VSNM in reducing the sequencing center effect (Supplementary Fig. 6A, B), so we focused on the ConQuR and VSNM results for subsequent analyses.

Per-batch AUROCs and AUPRs demonstrated equivalence between ConQuR and VSNM data types in every ML comparison, and both of these had similar per-batch performances to raw data counterparts (Fig. 2C, D, G; Supplementary Fig. 6C–E). For negative control analyses, we repeated all ML model assessments using two strategies: scrambled metadata labels and shuffled counts. In both cases, we found equivalent reductions in AUROCs (Supplementary Fig. 7) and AUPRs (Supplementary Fig. 8) that were significantly worse across all cancer types. These results strongly argue against systemic biases from VSNM or ConQuR, and show their equivalence for batch correction on these data (Supplementary Fig. 6A, B) and downstream ML (Fig. 2C, D, G).

We then calculated feature similarity comparisons between WIS-overlapping raw and normalized data types. We calculated both Kendall tau correlations (rank similarity) and Fisher exact tests (binary overlap), although externally constraining the feature space to 184 WIS genera can weaken the latter (i.e., WIS taxa are putatively cancer-associated and larger proportions of features are being used by the ML models). Notably, all rank-based comparisons were significantly similar between ML models built using the raw versus normalized data types (Fig. 2E, F, H; Supplementary Fig. 6F–H). Compared to raw data ML models, all ConQuR-based overlap comparisons showed significant enrichment (Fig. 2E, F, H), whereas 90.6% (58 of 64 cancer type comparisons) VSNM-based overlap comparisons had significant enrichment (Supplementary Fig. 6F–H). Additionally, the significance for ConQuR-based feature similarities were consistently higher than that for VSNM (note y-axes in Fig. 2E, F, H versus Supplementary Fig. 6F–H). Collectively, these data suggest that a microbiome-specific batch correction method (ConQuR) can indeed retain greater within-batch feature variability than a non-microbiome-specific batch correction method (VSNM), although for most cases the difference is in the *degree* of preservation rather than if they are preserved.

**Fig. 3:**
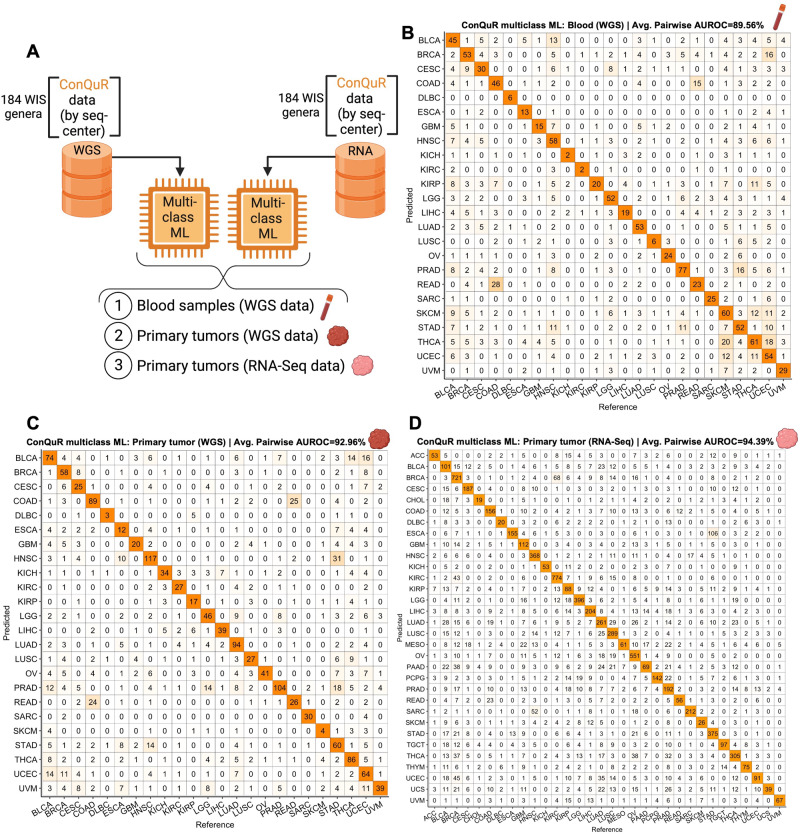
ConQuR-corrected, WIS-overlapping genera abundances demonstrate pan-cancer discrimination among blood and primary tumor samples across dozens of cancer types. **A** Diagram of data sources for the multiclass ML. Since ConQuR was limited to correcting one batch variable, sequencing center bias was corrected within WGS and RNA-Seq groups after subsetting analyses to Illumina HiSeq-processed samples. Multiclass ML was run independently on the ConQuR-corrected WGS and RNA-Seq groups; of note, blood samples were only in the WGS group. **B** Multiclass gradient boosting ML across 24 cancer types using all blood samples in TCGA. Average pairwise AUROC is denoted above the confusion matrix. No information rate: 9.1%, mean balanced accuracy: 73.2%. **C** Multiclass gradient boosting ML across 24 cancer types using all WGS primary tumors in TCGA. Average pairwise AUROC is denoted above the confusion matrix. No information rate: 8.5%, mean balanced accuracy: 76.9% (**D**) Multiclass gradient boosting ML across 32 cancer types using all RNA-Seq primary tumors in TCGA. Average pairwise AUROC is denoted above the confusion matrix. No information rate: 10.7%, mean balanced accuracy: 79.2%. **B**–**D** *P* values are all less than 2.2 × 10^-16^ for comparing the no information rate to the observed accuracy. See Supplementary Fig. 1C for list of TCGA cancer type abbreviations.

Both ConQuR-corrected (Fig. 3) and VSNM-corrected (Supplementary Fig. 9) WIS-overlapping genera provided pan-cancer discrimination using multiclass ML, in agreement with per-batch analyses of the raw data (Supplementary Figs. 10–15). Thus, with respect to batch correction, the use of VSNM, or a modern microbiome-specific method, ConQuR, are consistent with the raw data and do not change our original conclusions about cancer type-specific microbiomes.

### Claims of widespread database contamination were not supported with data

Gihawi et al. [13] additionally allege that the original microbial database was highly contaminated with human sequences, which, they claim, “create[d] the false appearance of bacteria” (see quotes in Supplementary Text 1.7). However, despite these claims, they did not analyze either of the two databases used in the original work for human sequences: an in-house, custom Kraken database with 59,963 genomes, and the Web of Life database [34] (hereon “WoLr1”) with 10,575 genomes. Notably, the smaller WoLr1 database has been publicly available for ~4 years, and although the custom Kraken database was too large to directly post online (>1.3 terabytes), we detailed how it was created [1]. Instead, the critiquing authors centered their claim on apparent differences in the published microbial read counts versus those from their pipeline, which used the T2T-CHM13v1.1 human reference to remove additional human reads from TCGA—typically first aligned to hg19 (i.e., the TCGA “legacy” pipeline [35])—followed by mapping these T2T-depleted reads against an independent KrakenUniq database (“MicrobialDB”). Neither T2T-CHM13v1.1 nor MicrobialDB existed prior to our original paper (Supplementary Fig. 1A), and to suggest that our study should have implemented tools that became available after it was published is unreasonable. Nevertheless, their untested database contamination claim relies on two assumptions: (i) the original databases contained substantial human contamination and (ii) the updated host depletion methods employed by the critiquing authors did not cause the observation of decreased microbial reads. In the next sections, we provide evidence demonstrating that these two assumptions are unsupported.

### Database contamination with human sequences was rare and did not drive cancer microbiome classifiers

**Fig. 4:**
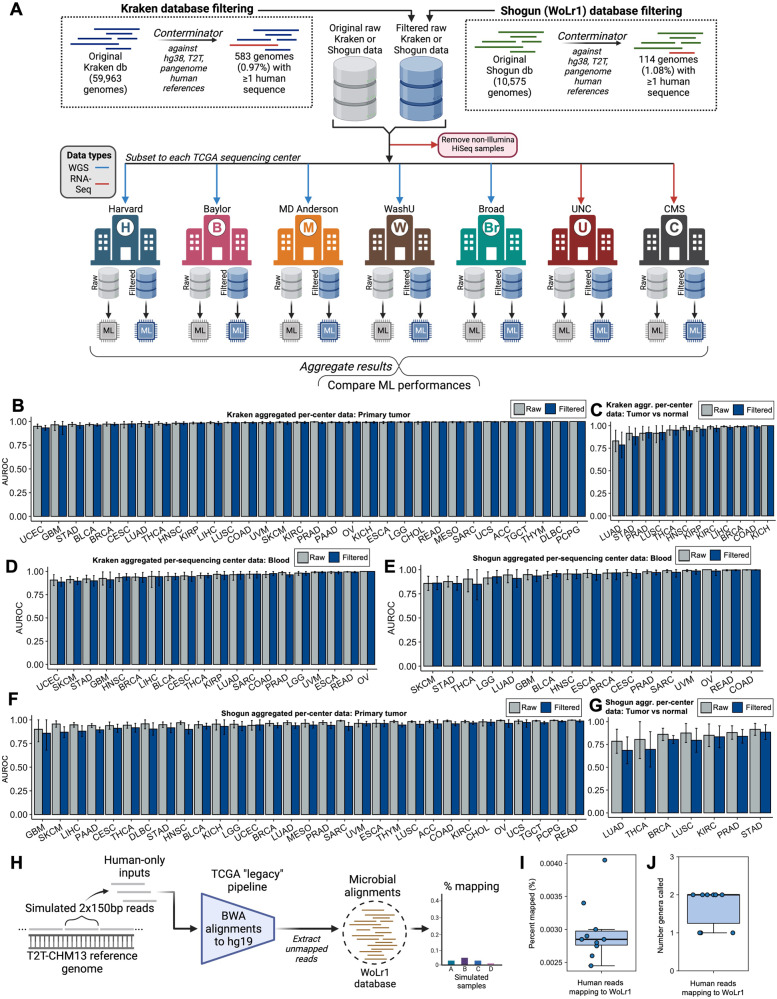
Human sequence contamination in the original databases was rare and does not impact downstream conclusions when all associated genera are removed. **A** Microbial genomes in the original two databases (custom Kraken, WoLr1) were processed with Conterminator [15] to identify any regions shared with hg38, T2T-CHM13, and human pangenome references, with ≤1.08% of microbial genomes affected in either database. Genus-level features in the associated count tables linked to any of these genomes with human contamination were removed to form filtered versions of the Kraken and SHOGUN/WoLr1 tables. A data splitting strategy was applied to compare filtered versus raw data. Aggregated AUROC data across all per-batch (**B**) primary tumor and (**C**) tumor versus normal, and (**D**) blood sample comparisons using the Kraken raw and filtered data. Aggregated AUROC data across all per-batch (**E**) blood sample (**F**) primary tumor, and (**G**) tumor versus normal comparisons using the SHOGUN/WoLr1 raw and filtered data. **H** Simulation diagram to evaluate false positive rate of human-only reads aligning to WoLr1 using the SHOGUN pipeline described in the original paper. Paired-end 150 bp Illumina reads from the T2T-CHM13 human reference were simulated using ART [64], subsampled to one million reads each, followed by initial mapping with BWA against hg19 and alignments of the unmapped reads against WoLr1. **I** Percent of human reads mapping against WoLr1 out of one million per sample. **J** Number of false positive genera based on human reads mapped against WoLr1. **B**–**G** Error bars denote 99% confidence intervals. See Supplementary Fig. 1C for list of TCGA cancer type abbreviations.

To evaluate the impact of human sequence contamination on the databases and downstream conclusions, we applied Conterminator [15], a state-of-the-art tool published by some of the critiquing authors in mid-2020, to identify any microbial genomes in the original Kraken database (Table S3**)** or WoLr1 (Table S4) sharing ≥1 Conterminator-identified human sequences with GRCh38 [36], T2T-CHM13v2.0 [37], or human pangenome (HPRC) [38] references (Fig. 4A, top; Methods). These analyses revealed that just 0.97% (583 of 59,963) of the Kraken database genomes, and just 1.08% (114 of 10,575) of the WoLr1 genomes, contained any amount of human sequence (Fig. 4A, top; Tables S3–S4); this represented ~0.0006% or 0.0005% of nucleotides in our Kraken database or WoLr1, respectively. As a conservative approach, we then identified and removed any genus-level features having ≥1 genome affected by human sequences in the originally published Kraken data (145 genera) and WoLr1 data (82 genera). We then compared ML performances between these filtered data versus their raw data counterparts within every individual batch; per-batch application limits the set of samples available but was chosen as a conservative approach to rule out concerns of batch correction. Per-batch performances were then aggregated to calculate confidence intervals for AUROC and AUPR. Importantly, we found overlapping AUROC and AUPR confidence intervals between the filtered and raw data for every Kraken-based ML comparison: one-cancer type versus all others using primary tumors or blood samples, or primary tumors versus adjacent normal tissues (Fig. 4B–D; Supplementary Fig. 16A–C). All blood and tumor-versus-normal ML comparisons using WoLr1 data similarly had equivalent performances, and 87.1% (27/31) of its primary tumor ML comparisons had overlapping AUROC and AUPR confidence intervals; moreover, the exceptions still had AUROCs ≥85% with filtered data (Fig. 4E–G; Supplementary Fig. 16D–F). We also repeated the above using only WIS-overlapping genera, with similar conclusions (Supplementary Fig. 17, Supplementary Text 1.8). These results show that database contamination with human sequences was minimal, that the number of genomes affected was very low, and that cancer type-specific classifiers were not dependent on the human sequences.

### Excluding the human reference in the original database did not materially affect the SHOGUN validation pipeline

In another section, Gihawi et al. [13] state, “their Kraken database did not include the human genome […] This dramatically increased the odds for human DNA sequences present in the TCGA reads to be falsely reported as matching microbial genomes.” This statement overlooked the identical conclusions we made using direct genome alignments with SHOGUN [39] as validation on 13,157 TCGA samples (Ext. Data Figs. 4i-4t in the original work). To quantitatively evaluate this hypothesis, we estimated what percentage of human reads and how many genera would have been false positives within the original TCGA SHOGUN pipeline (Fig. 4H). Specifically, we simulated 10 samples of 2 × 150 base pair Illumina paired end reads from the T2T-CHM13v2.0 human reference genome at 1x coverage, randomly subsampled each to a million reads, aligned them against hg19 using BWA (i.e., the legacy TCGA pipeline [35]), and aligned the non-hg19 reads with SHOGUN to the originally published WoLr1 database [34]. This mimics how we extracted non-human reads from pre-aligned TCGA files as an initial host depletion step, followed by processing the non-human reads for microbial abundances. Notably, among these human-only samples, just 0.002965% ± 0.0004558 of human reads mapped to WoLr1, or 1.7 ± 0.483 microbial genera (Fig. 4I, J). Given that the original SHOGUN data reported 1240 total genera, these results suggest false positive rates of ~0.1% genera and ~0.003% reads using the original SHOGUN pipeline. Thus, the SHOGUN results in the original manuscript were not materially affected by excluding a human reference in the underlying database, and its conclusions were the same as the original manuscript’s Kraken-derived data (Ext. Data Figs. 4i-4t in the original work).

### Microbial read differences are driven by non-contemporaneous host-depletion, and not by database contamination

**Fig. 5:**
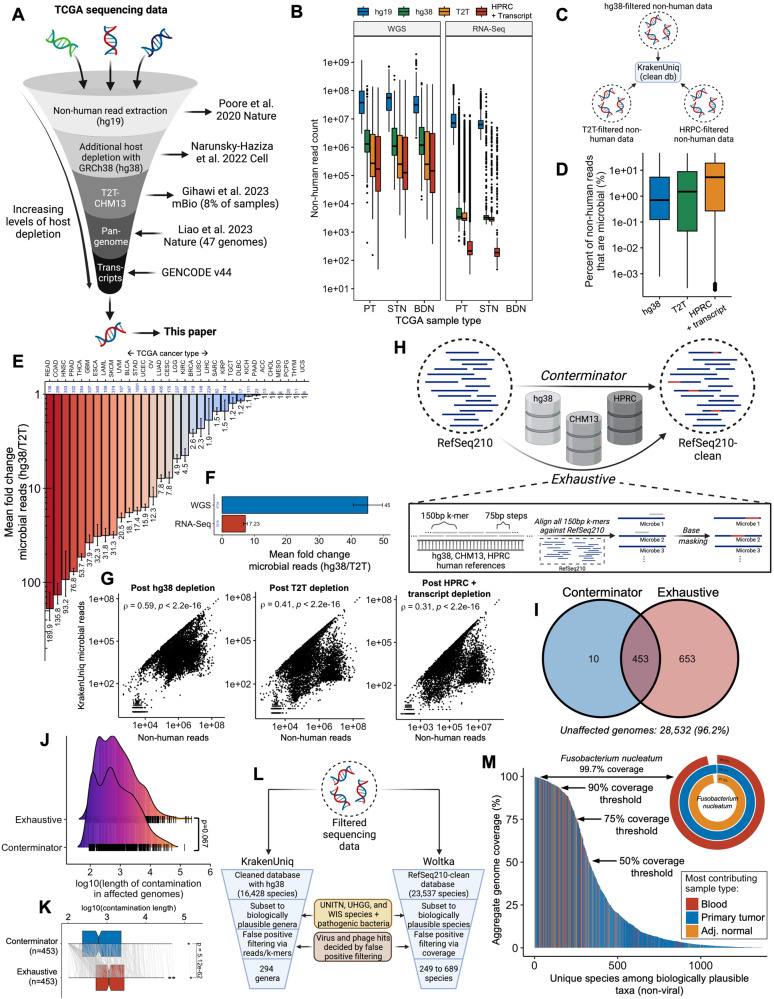
Sequential host depletion of TCGA reduces read counts without eliminating microbial signals. **A** Sequential host depletion steps of TCGA with respect to past literature and this current work. **B** Non-human TCGA read count totals after successive host depletion with hg19, hg38, T2T-CHM13, HPRC, and for RNA-Seq data additionally against GENCODE across the three major sample types: primary tumor (PT), solid tissue normal (STN), and blood derived normal (BDN). **C** Strategy for mapping hg38-, T2T-, and HPRC-transcript-depleted data with KrakenUniq against MicrobialDB, as used by Gihawi et al. [13], to compare microbial read counts. **D** Percent of hg38-, T2T-, and HPRC-transcript-depleted reads mapped by KrakenUniq to MicrobialDB as microbial. Y-axis is log10-transformed; zero-valued samples excluded. **E** Per-sample, per-cancer type mean fold changes (MFCs) in KrakenUniq-MicrobialDB counts between hg38- and T2T-depleted data. Per-cancer average MFCs inset below bars. Sample counts inset in blue above bars. Overlaid error bars denote standard errors. Zero-valued samples excluded to avoid ratios of infinity. **F** Per-sample, per-experimental strategy MFCs in KrakenUniq-MicrobialDB counts between hg38- and T2T-depleted data. Per-experimental strategy average MFCs inset to right of bars. Sample counts inset in blue to left of bars. Overlaid error bars denote standard errors. Zero-valued samples excluded to avoid ratios of infinity. **G** Scatter plots of non-human reads versus KrakenUniq-MicrobialDB counts for hg38-depleted (left), T2T-depleted (middle), and HPRC-GENCODE-depleted (right) data. Axes are log10-transformed; zero-valued samples excluded. Spearman correlation values inset with concomitant *p*-values. **H** Strategy for cleaning RefSeq version 210 (RS210) of human reads using Conterminator and Exhaustive with hg38, T2T, and HPRC reference genomes. Main steps of Exhaustive are visually described on bottom: all 150 bp k-mers with steps of 75 bp are aligned against RS210, and contiguously mapped regions are masked. **I** Number of RS210 genomes found to have at least one region of human sequence overlap detected by Conterminator and/or Exhaustive. **J** Unpaired, cumulative length of human sequence contamination in RS210 genomes found to have at least one region of human sequence overlap via Conterminator or Exhaustive. Wilcoxon test inset. **K** Paired cumulative length of human sequence contamination in 453 RS210 genomes found to have at least one region of human sequence overlap via Conterminator and Exhaustive. Wilcoxon rank-sum test inset. **L** Strategy for deriving filtered microbial abundances in TCGA using KrakenUniq against MicrobialDB or direct genome alignments against RS210-clean. Note that both MicrobialDB and RS210-clean occasionally include more than one genome per species. **M** Aggregate microbial genome coverages in TCGA among non-viral, human-associated species found in UNITN, UHGG, WIS, or pathogenic bacteria references. Each bar denotes a unique species, and the bar color denotes which TCGA sample type (primary tumor, blood, adjacent normal) had the highest amount of aggregate genome coverage by itself. Inset upper right: radial bar plots of aggregate genome coverage for *Fusobacterium nucleatum* in primary tumors (blue, 99.7%), blood (96.5%), and adjacent normal tissues (orange, 97.5%).

Computational host depletion comprises a series of steps to remove human reads prior to microbial classification (Fig. 5A). Our original paper [1] contained one step of host depletion via hg19 [35], which was updated to two-step host depletion using hg38 in our mycobiome work [28]. Gihawi et al. [13] processed ~8% of TCGA samples with T2T-CHM13v1.1 [40], a reference unavailable at the time of the original work, comprising a third step. To consider host depletion comprehensively, we included these steps and additionally filtered using T2T-CHM13v2.0 [37], the human pangenome (HPRC) [38], and the GENCODE v44 human transcript [41] references for RNA-Seq data.

Host depletion substantially lowers the number of non-human reads available for microbial classification in every TCGA cancer type (Fig. 5B; Supplementary Fig. 18A), and causes concomitant decreases in the number of microbial reads. We reported the latter when progressing from our original paper [1], which found 2.5% of total TCGA reads were microbial, to our mycobiome work [28], which found 0.08% of total reads were microbial, or a 31.25-fold decrease. Gihawi et al. [13] stated, without direct supporting evidence, that the lower number of microbial reads output from their pipeline is primarily the result of database contamination. Here, we show that the number of reads output from their pipeline is dependent on the inputted number of non-human reads (i.e., degree of host filtration). Specifically, we evaluated whether serial host depletion alone could cause substantive decreases in TCGA microbial read counts using the critiquing authors’ KrakenUniq pipeline with MicrobialDB (Fig. 5C), a database the authors emphasized “mitigated [false positives] by including the human genome and using only complete bacterial genomes” [13]. In other words, the critiquing authors’ conclusion of database contamination is confounded by their simultaneous addition of T2T-CHM13 host filtering.

Despite the multiple orders of magnitude decreases in non-human reads between hg19, hg38, T2T-CHM13v2.0, and pangenomes (Fig. 5B), we observe that the percentage of non-human reads that are microbial via KrakenUniq-MicrobialDB appear to concentrate as human depletion increases in stringency (Fig. 5D). In contrast, if this proportion had decreased with greater host depletion, it would suggest that the “non-human” reads were indeed human and not microbial. When calculated per-sample, and aggregated by cancer type, the mean fold changes (MFCs) in microbial reads between hg38- and T2T-based KrakenUniq-MicrobialDB reduced up to 189.9-fold (rectal adenocarcinoma [READ], Fig. 5E), with an average MFC of 24.6-fold. These reductions are relative to the larger drop of non-human reads overall (Fig. 5B). Similarly, even more extensive MFC decreases existed between hg38- and pangenome-based KrakenUniq-MicrobialDB comparisons, up to 204.1-fold (READ, Supplementary Fig. 19A), with an average MFC of 32.26-fold. Whole genome sequenced (WGS) samples experienced larger reductions on average than RNA-Seq samples (Fig. 5F; Supplementary Fig. 19B), as expected given that most of the improvements to newer genomes are in non-coding regions. Overall KrakenUniq-MicrobialDB microbial read counts were significantly correlated with input non-human reads but with decreasing effect sizes after each round of host depletion (Fig. 5G), suggesting that the true level of microbial reads is being asymptotically approximated. Collectively, these data demonstrate that the use of T2T-CHM13 directly confounded the critiquing authors’ conclusion that microbial read count reduction must imply database contamination, since their own database is described to lack human sequence contamination and included a human reference genome. Differences between these hg38- and T2T-based KrakenUniq-MicrobialDB TCGA data alone comprised 631 million microbial reads, and this may be even larger for hg19-versus-T2T comparisons, although we did not perform that analysis due to prohibitive computational cost.

### Development of a generalizable, sensitive method for database cleaning

Using only complete bacterial genomes for mapping microbial reads (as in MicrobialDB) is undesirable, because this precludes utilization of human-associated metagenome assemblies [42, 43] or public databases like RefSeq [44]. We thus developed a generalizable, sensitive method for microbial database cleaning of human sequences called “Exhaustive,” using RefSeq version 210 (“RS210”) as an example, and benchmarked it against Conterminator (Methods).

Exhaustive comprises a one-time, computationally-intensive process that aligns all 150 base pair sequences from hg38, T2T-CHM13v2.0, and HPRC human reference genomes, with 75 base pair steps, against a microbial database of interest (Fig. 5H). All contiguously mapped regions in the microbial database are subsequently masked to “Ns” to provide a human-cleaned counterpart. Parallel evaluation of Conterminator and Exhaustive against RS210 with hg38, T2T-CHM13v2.0, and HPRC human genomes demonstrated that Exhaustive was 2.38-times more sensitive (1106 versus 463 contaminated genomes in RS210; Fig. 5I), and identified regions of similar length overall (Fig. 5J), but significantly longer regions when restricting to genomes found by both methods (Fig. 5K). Repeating these analyses with the WoLr1 database revealed similar results, with Exhaustive providing 2.01-times greater sensitivity than Conterminator (229 versus 114 contaminated genomes), and again identified significantly longer regions on overlapping genomes (Supplementary Fig. 18B–D). As a conservative measure, we masked any bases flagged by Conterminator or Exhaustive to create a cleaned version of RS210 (“RS210-clean”; Fig. 5H). Simulations of this host depletion pipeline with RS210-clean using human-only sequences provided an estimated false positive rate of 1 in 60 million reads (Supplementary Fig. 18E, F, Supplementary Text 1.9).

### Bioinformatic pipelines implementing host read removal and human-cleaned databases reveal hundreds of human-associated microbial species in TCGA

To verify that the existence of microbes in TCGA is independent of the bioinformatic pipeline, we applied the critiquing authors’ workflow of T2T-CHM13 host depletion followed by KrakenUniq with MicrobialDB (Fig. 5L, left), in addition to performing direct genome alignments of hg38-T2T-HPRC-GENCODE-depleted data against RS210-clean (Fig. 5L, right). To further improve host removal, we used T2T-CHM13v2.0, rather than T2T-CHM13v1.1 as suggested [13], to include a more complete Y-chromosome. With the goal of performing a sensitivity analysis, we required taxa to be human-associated by overlapping with one or more of the following: the UNITN [42] or UHGG [43] human-associated metagenome assemblies; WIS-derived decontaminated bacteria [2] or fungi [28]; or bacterial species known to be pathogenic to humans [45]. To further eliminate false positives within this taxa subset, we performed additional filtering using combined read and k-mer counts for KrakenUniq-MicrobialDB data, or using ≥50% aggregate microbial genome coverages for RS210-clean data (Methods). Since virus and phage human associations remain poorly characterized in the literature, only genera/species that passed the restrictive KrakenUniq-MicrobialDB or RS210-clean filtering were kept for downstream analyses. These steps provided 294 genera in KrakenUniq-MicrobialDB data (Table S5**)** and 689 to 249 species in RS210-clean when varying the genome coverage filter between 50% and 90% (Table S5–S6). Remarkably, in RS210-clean data, hundreds of human-associated, non-viral species had nearly complete genome coverages among blood or tumor samples, including numerous orally-derived bacteria (Fig. 5M, Supplementary Fig. 19C, Supplementary Text 1.10, Tables S5–S6). We next considered whether these taxa with rigorous bioinformatic evidence would verify the existence of cancer type-specific microbiomes, beginning with the critiquing authors’ T2T-KrakenUniq-MicrobialDB pipeline.

### Applying the T2T-KrakenUniq-MicrobialDB pipeline to all of TCGA verifies the existence of cancer type-specific microbiomes

Gihawi et al. [13] analyzed the microbiomes of 1255 TCGA samples (8.1% of total in [28]) from three cancer types (bladder [BLCA], head and neck [HNSC], and breast [BRCA]), and ended their manuscript by saying, “the near-perfect association between microbes and cancer types […] is, simply put, a fiction” [13]. However, the authors never examined their own data for cancer type differences. Indeed, re-analyzing their supplementary raw data demonstrates that cancer type-specific microbiomes among BLCA, HNSC, and BRCA are clearly found in TCGA tissues and blood (Supplementary Fig. 20A–G), including when subsetting to WIS-overlapping genera (Supplementary Fig. 20H, I), or a subset of just 9 WIS-overlapping, human-associated genera (Supplementary Fig. 20J–S). This was true across measures of alpha diversity, beta diversity, log-ratios, or ML classification performance (Supplementary Fig. 20B–I, L–S). Thus, the critiquing authors’ own data supports the existence of cancer type-specific microbiomes in TCGA.

**Fig. 6:**
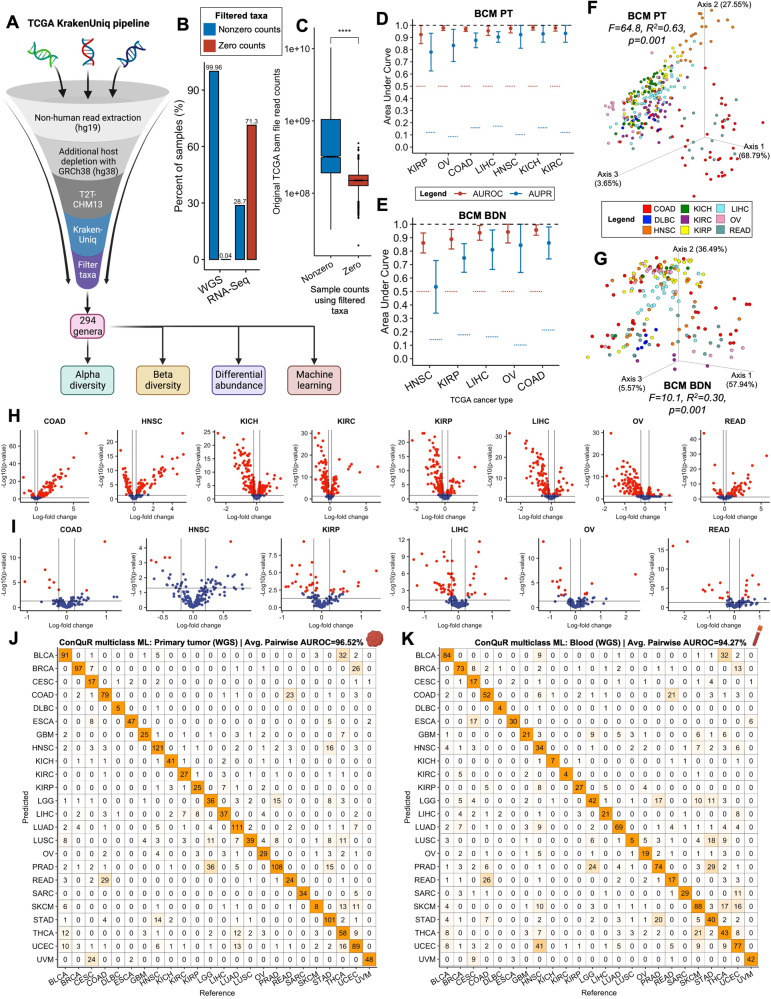
Application of the KrakenUniq-MicrobialDB pipeline on T2T-depleted TCGA data demonstrates cancer type-specificity in tissues and blood. **A** Steps taken to derive 294 filtered genera based on running KrakenUniq mapping against MicrobialDB on T2T-depleted TCGA data. Genera abundances were input into alpha diversity, beta diversity, differential abundance, and ML analyses to evaluate cancer type-specificity. **B** Prevalence of filtered genera among TCGA WGS and RNA-Seq samples. **C** Original TCGA read depths of samples containing (blue) or lacking (red) filtered genera. **D** Exemplary one-cancer-type-versus-all-others ML among primary tumors (PT) at Baylor College of Medicine (BCM) using the filtered genera. Error bars denote averages (dots) and 95% confidence intervals (brackets) of 10-fold cross-validation. Null AUROC and AUPR shown as dotted horizontal lines. **E** Exemplary one-cancer-type-versus-all-others ML among blood derived normals (BDN) at BCM using the filtered genera. Error bars denote averages (dots) and 95% confidence intervals (brackets) of 10-fold cross-validation. Null AUROC and AUPR shown as dotted horizontal lines. **F** Aitchison distance beta diversity among BCM PTs colored by cancer type. PERMANOVA values inset, based on cancer type separation. **G** Aitchison distance beta diversity among BCM BDNs colored by cancer type. PERMANOVA values inset, based on cancer type separation. **H** Filtered genera differential abundance among BCM cancer types using PTs in a one-cancer-type-versus-all-others manner. Red dots denote microbes with *q* ≤ 0.05. Positive log-fold changes denote microbes associated with that particular cancer type. **I** Filtered genera differential abundance among BCM cancer types using BDNs in a one-cancer-type-versus-all-others manner. Red dots denote microbes with *q* ≤ 0.05. Positive log-fold changes denote microbes associated with that particular cancer type. **J** Multiclass ML confusion matrix among cancer types using WGS PTs after ConQuR batch correction. **K** Multiclass ML confusion matrix among cancer types using WGS BDNs after ConQuR batch correction. **D**–**E**, **H**–**K** TCGA cancer type abbreviations shown in Supplementary Fig. 1C.

We extended this analysis to all available TCGA samples. We applied the KrakenUniq-MicrobialDB pipeline on all 15,512 hg38-depleted TCGA samples profiled in our mycobiome manuscript [28] after additional host depletion with T2T-CHM13v2.0 (Fig. 6A; filtered genera abundances in Table S8 and metadata in Table S9). As we previously noted, the number of samples decreased from our original paper [1] after hg38 depletion (18,166 to 15,512), of which ~97% were RNA-Seq samples of lower total read depth [28]; however, extra host depletion with T2T-CHM13v2.0 did not cause further sample dropout. Conservatively, we then only examined 294 human-associated genera passing read count and unique k-mer filtering (Fig. 5L; Methods), followed by evaluating their abundances for cancer type-specific differences across alpha diversity, beta diversity, differential abundance, and ML without or with ConQuR [14] batch correction (Fig. 6A). Nearly all WGS samples contained one or more of these filtered taxa, but fewer RNA-Seq samples did (Fig. 6B), and, overall, samples lacking them had significantly fewer total reads (Wilcoxon: *p* < 2.2 × 10^−16^; Fig. 6C). Filtered microbial reads significantly varied across cancer types within every TCGA sequencing center (all Kruskal-Wallis tests: *p* < 6.8 × 10^−16^; Supplementary Fig. 21A–G) but revealed low read counts among RNA-Seq samples from the University of North Carolina (UNC; Supplementary Fig. 21E), which were dropped from further analysis.

Calculating alpha diversity within each sequencing center subset for primary tumors and blood revealed significant richness variation among cancer types in every analysis (all Kruskal-Wallis tests: *p* < 3.1 × 10^−3^; Supplementary Fig. 22A, K). ML analysis using the raw data within every sequencing center similarly found that all primary tumor and blood-based comparisons of cancer types were significantly above their expected null performances (Fig. 6D, E; Supplementary Fig. 22M, N, P–S, U–W). Repeating this process for tumor versus normal ML demonstrated significantly better-than-null performances for multiple kidney cancers (KIRC, KICH; Supplementary Fig. 22L), liver cancer (LIHC; Supplementary Fig. 22L), and stomach cancer (Supplementary Fig. 22O, X), but not for lung cancers (LUAD, LUSC; Supplementary Fig. 22O, T). Beta diversity analyses with robust Aitchison distances of the raw abundances among primary tumor tissues and blood samples from every sequencing center revealed significant per-cancer variation (all PERMANOVAs [999 iterations]: *p* = 0.001; Fig. 6F, G; Supplementary Fig. 23). We then computed one-cancer-type-versus-all-other differential abundances with ANCOM-BC [46] on the raw abundances within every sequencing center, finding that all primary tumor and blood sample comparisons had significantly differentially abundant microbes (Fig. 6H, I; Supplementary Fig. 24).

To combine all WGS samples, we corrected sequencing center bias with ConQuR (Supplementary Fig. 25A) (Methods). ConQuR batch correction used sample type (e.g., primary tumor [PT], blood derived normal [BDN], adjacent solid tissue normal [STN]), and not cancer type, for supervised correction, but the cancer type signal nonetheless slightly increased with batch correction (19.4 to 21.6% variance) while WGS sequencing center bias decreased by 1.9-fold (9.3 to 4.9%) (Supplementary Fig. 25B). We then applied multiclass ML to discriminate among all WGS primary tumors simultaneously, finding an average pairwise AUROC of 96.52% and mean balanced accuracy of 82.53% that was significantly greater than the no information rate (NIR) of 8.30% (*p* < 2.2 × 10^−308^; Fig. 6J). Repeating multiclass ML among all blood samples, which were only WGS, provided an average pairwise AUROC of 94.27% and mean balanced accuracy of 77.59% that was significantly greater than the NIR of 8.96% (*p* < 2.2 × 10^−308^; Fig. 6K). For completeness, we also calculated multiclass ML using the CMS RNA-Seq raw data, finding an average pairwise AUROC of 97.22% and mean balanced accuracy of 91.95% that was significantly greater than the NIR of 50.52% (*p* = 2.0 × 10^−264^; Supplementary Fig. 22Y). Subsequent head-to-head analyses between ConQuR and raw data did not reveal artifactual enhancements of per-center data among tumor and blood comparisons (Supplementary Fig. 25; Supplementary Text 1.11).

### KrakenUniq-MicrobialDB-derived cancer microbiome discrimination and signatures are stable over three levels of host depletion

**Fig. 7:**
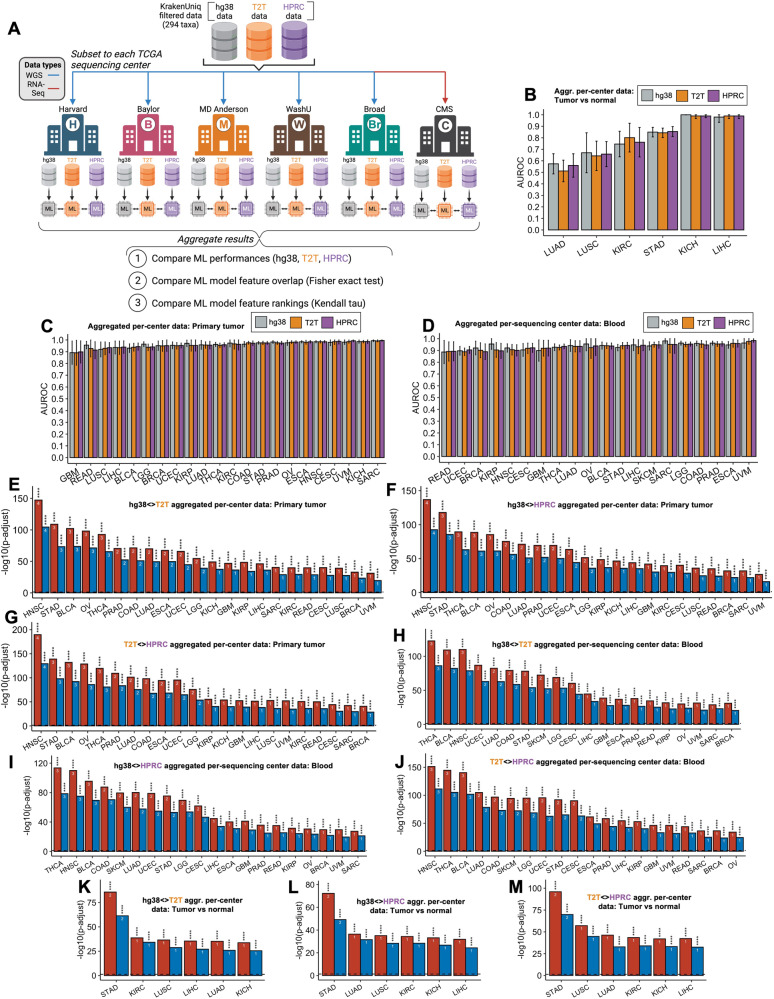
Cancer microbiome signals are consistent across three levels of host depletion. **A** Data splitting strategy for comparing per-batch ML performances and signatures between raw KrakenUniq-MicrobialDB abundances derived from hg38-, T2T-, and HPRC-transcript-depleted data. Note: Only Illumina HiSeq samples were used for comparison. **B** Aggregated per-batch AUROCs for tumor-versus-normal ML comparisons. **C** Aggregated per-bach AUROCs for cancer type comparisons using primary tumors. **D** Aggregated per-bach AUROCs for cancer type comparisons using blood samples. ML feature similarities for cancer type comparisons when using primary tumors between (**E**) hg38- versus T2T-depleted, (**F**) hg38- versus HPRC-transcript-depleted, and (**G**) T2T- versus HPRC-transcript-depleted microbial data. ML feature similarities for cancer type comparisons when using blood samples between (**H**) hg38- versus T2T-depleted, (**I**) hg38- versus HPRC-transcript-depleted, and (**J**) T2T- versus HPRC-transcript-depleted microbial data. ML feature similarities for tumor versus normal comparisons between (**K**) hg38- versus T2T-depleted, (**L**) hg38- versus HPRC-transcript-depleted, and (**M**) T2T- versus HPRC-transcript-depleted microbial data. **B**–**D** Error bars denote 95% confidence intervals. **E**–**M** Kendall tau correlations are shown in red. Fisher exact tests are shown in blue. *P*-values combined across multiple batches using Fisher’s method on the raw per-batch *p*-values, followed by Benjamini-Hochberg correction across cancer types. Number of combined batches per cancer type inset in white text. Logarithms are base 10. See Supplementary Fig. 1C for list of TCGA cancer type abbreviations.

Since we already calculated KrakenUniq-MicrobialDB-derived microbial reads individually on hg38-, T2T-, and HPRC-depleted data (Fig. 5C; hg38 filtered genera abundances in Table S10; T2T filtered genera abundances in Table S11; HPRC filtered genera abundances in Table S12), and introduced methods to compare ML performance and signature similarities (cf. Fig. 1A), we next evaluated whether serial host depletion impacted the ability to identify consistent cancer microbiome signals. Specifically, we calculated within-center ML classification accuracy using raw data across primary tumors, blood samples, and tumor-versus-normal comparisons with the same 294 human-associated, T2T-filter-passing genera across all overlapping WGS and RNA-Seq samples (Fig. 7A). Aggregated per-batch AUROCs and AUPRs were equivalent for all ML comparisons (Fig. 7B–D), and perhaps more importantly, the ML signatures were significantly similar for every pairwise data type comparison among primary tumors (Fig. 7E–G), blood samples (Fig. 7H–J), and tumor versus normals (Fig. 7K–M). These data demonstrate remarkable stability of cancer type-specific findings across multiple levels of host depletion, despite concomitant microbial read decreases comprising hundreds of millions of reads (cf. Figure 5E; Supplementary Fig. 19A). These data further argue against the critiquing authors’ implication that fewer total reads discredits cancer type-specific conclusions.

### Well-covered, human-associated microbial species in TCGA verify the existence of cancer type-specific microbiomes

**Fig. 8:**
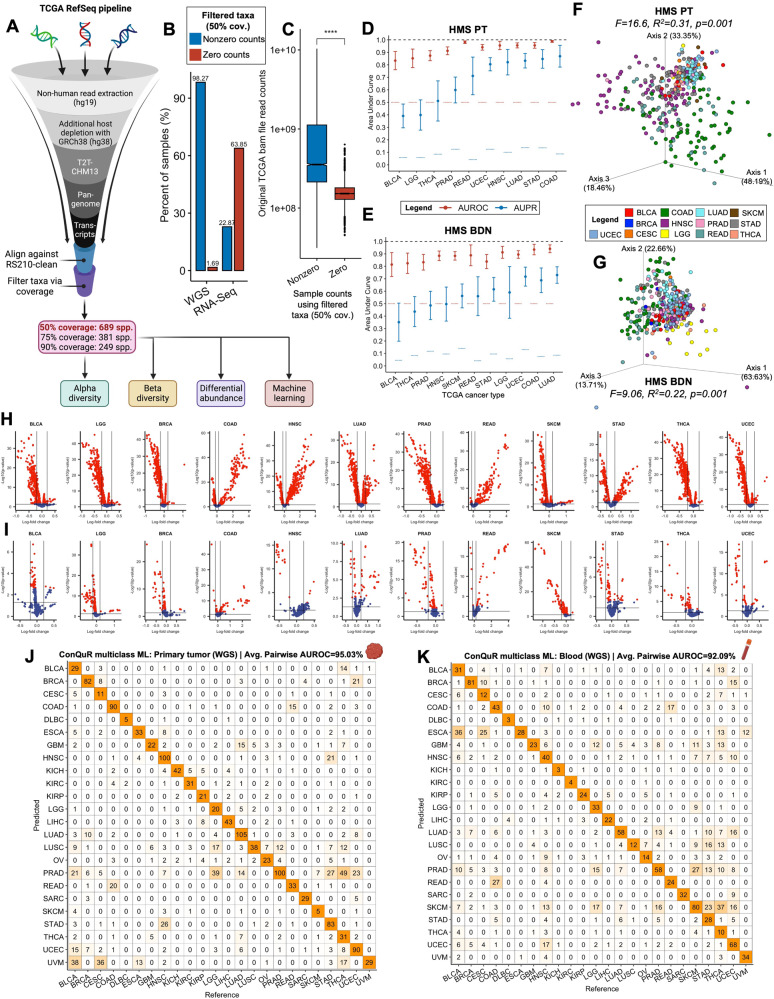
Application of the SHOGUN/Woltka-RS210-clean pipeline on HPRC and transcript-depleted TCGA data demonstrates cancer type-specificity in tissues and blood. **A** Steps taken to derive 689 unique, filtered species with ≥50% aggregate genome coverage based on running SHOGUN/Woltka mapping against RS210-clean on HPRC and transcript-depleted TCGA data. Species abundances were input into alpha diversity, beta diversity, differential abundance, and ML analyses to evaluate cancer type specificity. **B** Prevalence of filtered species among TCGA WGS and RNA-Seq samples. **C** Original TCGA read depths of samples containing (blue) or lacking (red) filtered species. **D** Exemplary one-cancer-type-versus-all-others ML among primary tumors (PT) at Harvard Medical School (HMS) using the filtered species. Error bars denote averages (dots) and 95% confidence intervals (brackets) of 10-fold cross-validation. Null AUROC and AUPR shown as dotted horizontal lines. **E** Exemplary one-cancer-type-versus-all-others ML among blood derived normals (BDN) at HMS using the filtered species. Error bars denote averages (dots) and 95% confidence intervals (brackets) of 10-fold cross-validation. Null AUROC and AUPR shown as dotted horizontal lines. **F** Aitchison distance beta diversities calculated by RPCA [77] among HMS PTs colored by cancer type. PERMANOVA values inset, based on cancer type separation. **G** Aitchison distance beta diversities calculated by RPCA [77] among HMS BDNs colored by cancer type. PERMANOVA values inset, based on cancer type separation. **H** Filtered species differential abundance among HMS cancer types using PTs in a one-cancer-type-versus-all-others manner. Red dots denote microbes with *q* ≤ 0.05. Positive log-fold changes denote microbes associated with that particular cancer type. **I** Filtered species differential abundance among HMS cancer types using BDNs in a one-cancer-type-versus-all-others manner. Red dots denote microbes with *q* ≤ 0.05. Positive log-fold changes denote microbes associated with that particular cancer type. **J** Multiclass ML confusion matrix among cancer types using WGS PTs after ConQuR batch correction. **K** Multiclass ML confusion matrix among cancer types using WGS BDNs after ConQuR batch correction. **D**–**E**, **H**–**K** TCGA cancer type abbreviations shown in Supplementary Fig. 1C.

Having comprehensively demonstrated cancer type-specific conclusions with the T2T-KrakenUniq-MicrobialDB pipeline, we repeated all analyses using direct genome alignments of hg38-T2T-HPRC-GENCODE-depleted data against RS210-clean (genome level abundances Table S13 and metadata in Table S14; Figs. 5L, 8A). To our knowledge, this is the most host depleted version of TCGA in existence, and yet 90.77% (14,080 of 15,512) of samples still had microbial hits against RS210-clean with 1.2 billion total microbial reads. Nonetheless, for conservative analyses, we restricted all downstream analyses to 689 unique species with ≥50% genome coverage and enforced known human associations for all non-viral species (Fig. 8A). Nearly all WGS samples had one or more of these filtered species whereas their prevalence among RNA-Seq samples was lower (Fig. 8B), and samples without filtered species had significantly fewer total reads (Fig. 8C). Within every TCGA sequencing center, filtered microbial read counts significantly varied across cancer types (all Kruskal-Wallis tests: *p* < 2.2 × 10^−8^; Supplementary Fig. 26A–G), but again revealed low read counts among RNA-Seq samples from the University of North Carolina (UNC; Supplementary Fig. 26E), which were dropped from further analysis. Analogous to the T2T-KrakenUniq-MicrobialDB analyses, we also focused on Illumina HiSeq-processed samples for downstream analyses to remove a large batch effect (Methods).

Alpha diversities significantly varied among cancer types for primary tumors and blood samples within every sequencing center (all Kruskal-Wallis tests: *p* < 2.8 × 10^-4^; Supplementary Fig. 27A–K). One-cancer-type-versus-all-others ML with the raw data showed that every sequencing center had significantly higher predictive performance than null for primary tumors and blood (Fig. 8D, E; Supplementary Fig. 27M, N, P–S, U–W). Tumor-versus-normal ML demonstrated significantly better-than-null performances for kidney chromophobe cancer (KICH; Supplementary Fig. 27O), liver cancer (LIHC; Supplementary Fig. 27O), stomach cancer (Supplementary Fig. 27L, X), and lung squamous cell carcinoma (LUSC; Supplementary Fig. 27T), but not for lung adenocarcinoma (LUAD; Supplementary Fig. 27L) or kidney clear cell carcinoma (KIRC; Supplementary Fig. 27O). All robust Aitchison distance beta diversity analyses using the raw abundances in primary tumors or blood showed significant cancer type variation (all PERMANOVAs [999 iterations]: *p* = 0.001; Fig. 8F, G; Supplementary Fig. 28). Similarly, all primary tumor and blood comparisons showed significantly differentially abundant microbes within every sequencing center subset (Fig. 8H, I; Supplementary Fig. 29).

After combining all WGS samples across sequencing centers with ConQuR (Supplementary Fig. 30A, B), multiclass ML provided primary tumor-based discrimination among 24 cancer types simultaneously with an average pairwise AUROC of 95.03% and mean balanced accuracy of 78.82% that was significantly greater than the NIR of 8.48% (*p* < 2.2 × 10^−308^; Fig. 8J). Multiclass ML among blood samples from 24 cancer types similarly showed an average pairwise AUROC of 92.09% and mean balanced accuracy of 74.60% that was significantly greater than the NIR of 8.94% (*p* < 2.2 × 10^−308^; Fig. 8K). Calculating multiclass ML with raw data from CMS-sequenced RNA-Seq samples revealed similar results (Supplementary Fig. 27Y). We also verified that ConQuR did not artifactually bias cancer-type specific conclusions among per-batch subsets (Supplementary Fig. 30C–H). Therefore, using the most stringently depleted version of TCGA to date alongside a thoroughly human-cleaned database while restricting analyses to well-covered, human-associated taxa, we continue to reproduce the cancer type-specific conclusions from our original paper, published years before these methods and resources became available. Because many researchers are interested in the technical details of the strengths/weaknesses of different choices for steps in the computational workflow, we have summarized our recommendations in Supplementary Fig. 31.

### Taxon feature lists and similarities between original and state-of-the-art pipeline

Taxon feature lists and variable importance for the multiclass classifiers can be found in Tables S15–S18, and per-taxon lists of cancer-associated taxa with statistical significance per ANCOM-BC [46] after ConQuR correction are in Tables S19, S20 (see Supplementary Text 1.12 for further details). Although the currently recommended pipeline (Supplementary Fig. 31) substantively differs from our original work [1]—distinct and improved mapping algorithms, databases (a custom Kraken db vs. RefSeq), and resolution of the features (genera vs. per-genome abundances)—we sought to compare their taxon similarities for distinguishing cancer tissue of origin (TOO), which represents a common diagnostic goal, especially in liquid biopsies [47]. We thus computed multiclass ML on VSNM- and ConQuR-corrected, full-feature original data (Supplementary Fig. 32, 33), followed by comparing their feature lists (Tables S21, S22) to RS210-clean, ConQuR-corrected multiclass features (Table S18; cf. Fig. 8J–K, Supplementary Fig. 27Y) using Fisher’s exact tests and Kendall tau correlations (Methods). We find that using ConQuR-corrected old and new data, the feature lists are highly correlated (*p* < 0.01 on all measures and sample types), and that in most situations, even the original VSNM features and ConQuR-corrected RS210-clean features are highly correlated despite the difference in normalization methods (Table S23).

## Discussion

Though in its infancy, the cancer microbiome field has made major strides over the last 5 years in microbial characterization, and actively continues to improve the available repertoire of tools for sensitively detecting low-biomass microbes while excluding contaminants [48, 49]. We have been encouraged by the subsequent rapid release of manuscripts from independent labs that validated the cancer type-specific conclusions we had found in TCGA, including efforts that used up to 811 experimental contamination controls and/or applied orthogonal methods (e.g., imaging, cultivation, cell-free RNA-Seq) [2, 3, 9].

Our updated analyses, using modern techniques and resources, have demonstrated thorough support of the original findings relative to each concern raised by Gihawi et al. [13]. When examining our original databases for human sequences, we find detectable but rare (≤1.1% of genomes used) examples that were noncontributory to the original conclusions that cancer type-specific microbiomes exist (Fig. 4, Supplementary Fig. 17A–G). Simulations also suggested that immaterial amounts of human content was mapped to microbes in our prior SHOGUN validation analyses (Fig. 4H–J), which replicated all Kraken-based conclusions in our original manuscript [1]. We then found that the degree of host depletion directly confounded the critiquing authors’ argument that fewer microbial reads must imply database contamination (Fig. 5A–G), with their own pipeline differing by 631 million microbial reads between hg38- and T2T-depleted data. Because many microbiome tools have moved beyond databases of complete genomes [50, 51], alongside the development of human-associated metagenome assemblies [42, 43], we developed a generalizable method for cleaning microbial databases from human sequences called Exhaustive that is empirically twice as sensitive as Conterminator (Fig. 5H–K; Supplementary Fig. 18B–D). After creating RS210-clean, direct genome alignments demonstrated that hundreds of human-associated species had substantial aggregate genome coverages in TCGA (Fig. 5M; Supplementary Fig. 19C). We then showed that cancer type-specific microbiomes were clearly evident using both the critiquing authors’ KrakenUniq-MicrobialDB pipeline (Fig. 6), including with stable microbial signals across multiple levels of host depletion (Fig. 7), or with our direct alignment pipeline against RS210-clean (Fig. 8).

Using re-analyses of the originally published data, we find no systematic bias caused by VSNM that enabled cancer type identification, and that comparisons of the normalized data to raw data within every individual batch provide equivalent ML performance and significantly similar model features (Fig. 1, Supplementary Figs. 2–5). We re-confirmed the lack of any systematic bias of VSNM using WIS-overlapping features, and its ML performance equivalence with raw data subsets and later-published, microbiome-specific batch correction tools, ConQuR and MMUPHin (Fig. 2, Supplementary Figs. 6–9). This additionally prompted us to evaluate whether ConQuR-corrected data provided pan-cancer discrimination in blood and tumor samples, which indeed found average AUROCs of ~90% and higher among dozens of cancer types (Fig. 3). Evaluating ConQuR alongside VSNM also provided insight into how a microbiome-specific tool better preserves the degree of per-batch feature variation (cp. Figure 2E, F, H and Supplementary Fig. 6F–H), but this must be counterbalanced against practical dataset considerations such as the number of batch factors. Although results were similar, we generally recommend ConQuR because it is more compatible with downstream workflows than VSNM, more effective than MMUPHin, and because it does not require log-transform of the data and conversion back to per-sample counts. However, we did find that ConQuR can, in some circumstances, introduce technical artifacts that VSNM does not (Supplementary Fig. 25B, 30B), possibly because of the user choices that the supervised aspects of the method require. Collectively, these re-analysis efforts thoroughly support the reliability and integrity of the originally published data and findings, including using WIS taxonomic subsets previously cited by the critiquing authors to justify their own work [30, 31].

Related to concerns of data distribution changes between raw and VSNM-corrected data in the original study [13], we note that all microbiome batch correction methods modify the underlying distribution (abundance, presence/absence) to reduce variance attributed to technical factors. For example, ConQuR adjusts taxonomic counts and prevalence based on a reference batch, and thus “biases” all non-reference samples to look more like the reference batch [14]. This batch correction bias can only be avoided with proper experimental design and otherwise comprises a necessary cost for using the full dataset. Importantly, one must consider direct and indirect ways in which biological information can leak into this batch correction process, including technical variables themselves (e.g., sequencing centers) when biological phenotypes (e.g., cancer types) are not equally distributed. Our original application of VSNM explicitly avoided using cancer type information (Supplementary Text 1.3), but, due to how TCGA was collected, two technical variables (sequencing center, hospital of origin) did not have equal distribution of cancer types. Although it is theoretically possible that correcting for these two technical factors could have indirectly introduced cancer type information, the practical importance of this factor is minimal, because both uncorrected and VSNM-corrected data had equivalent per-batch ML performances and significantly similar features (Fig. 1, Supplementary Figs. 2, 3). Because the degree to which direct and indirect information in batch correction influences downstream analyses is rarely known a priori, we strongly recommend retrospective per-batch comparisons between uncorrected and corrected data that verify the robustness of those conclusions, as shown here, as well as negative control analyses (e.g., Fig. 1, Supplementary Figs. 2–8).

Having demonstrated the above, we find it important to explicitly state that computational methods to analyze cancer microbiome data continue to rapidly improve, and the field’s awareness of which areas are most critical to address is also evolving, thereby implying that methods even a few months old can become outdated. For example, today we would implement stronger host depletion measures and taxonomic filtering (e.g., by coverage, pathogenicity) than reflected in our original manuscript, and indeed we applied such methods in our later work [28]. Doing so may decrease the number of microbial reads, but our data suggest that the conclusions regarding cancer type-specific microbiomes remain intact. We anticipate that better, and more diverse, human pangenome references, decontamination algorithms, batch correction methods, and taxonomic annotations are likely to be published in the next few years; similarly, efforts such as CAMI [52] highlight that no currently available taxonomic assignment tool is perfect, and implausible taxon assignments are likely to remain a challenge for the whole microbiome field for some time. These considerations do not automatically invalidate conclusions and analyses from our 2020 paper. We thus expect and advocate for newer methods to keep pushing the sensitivity and specificity of cancer microbe detection, and that, until “gold-standard” pipelines can be agreed upon, continuing to find tumor-specific microbial signatures using diverse computational and experimental workflows by independent labs provides reassuring evidence of their presence and utility.

To conclude, being able to thoroughly and confidently profile the cancer microbiome holds great promise to improve all aspects of patient care and, ultimately, patient outcomes [53]. Progress in this space has led to rapidly evolving analytic tools that will help us better elucidate the contribution of the microbiome in oncogenesis.

## Materials and methods

### Data accession

#### Processed TCGA files

The originally published, quality-controlled Kraken raw and Voom-SNM-normalized abundances tables (*n* = 17,625), the raw SHOGUN/WoLr1 abundance table (*n* = 13,517), and TCGA metadata (*n* = 17,625) were accessed from Poore and Kopylova et al. [1] and used for downstream processing. Details on how those tables were created are described in the associated manuscript. WIS taxa were derived from a “hit” list of microbes shared by the first author of Narunsky-Haziza et al. [28], which contained bacterial and fungal genera across all tissue samples (tumors, NATs, or true normals [breast only]) from that study and the study by Nejman et al. [2]. Specifically, these “hits” represented microbes with genus-level evidence that passed decontamination based on 811 experimental contamination controls for bacteria [2], and 295 controls for fungi [28]. Since fungi were not considered or profiled in our original manuscript, only the bacterial genera were intersected with the Kraken and SHOGUN/WoLr1 data, and used for consideration here. Overall, 84.4% of WIS genera (184 total) were found in the original Kraken data.

#### Raw host depleted TCGA files

Per-sample sequence data were obtained from Qiita [54] studies 13722 and 13767 (WGS and RNA-Seq respectively). These data were already human filtered against GRCh38 as described in Narunsky-Haziza et al. [28]. The per-sample data were filtered with fastp as described below, and human depleted either with T2T-CHM13v2.0 or with T2T-CHM13v2.0 in addition to the human pangenome and GENCODE v44.

### Data installations

Unless otherwise noted, software was installed from Bioconda [55].

### Host depletion pipeline

The sequence data were filtered for all adapters known to fastp [56] (version 0.23.4) in paired end mode by explicitly specifying a known adapters file composed of the adapters used by fastp at compile time. This was done to avoid its autodetection which is limited to the first 100,000 sequences, to allow for removal of a large number of possible adapters, and ensure application of adapter removal in paired end mode as it is implicitly disabled otherwise. Sequences shorter than 45 nucleotides were removed with “-l 45”. Each sample was then filtered against each genome in the human pangenome [38], as well as both T2T-CHM13v2.0 (ref. [40]) and GRCh38 (ref. [36]), using minimap2 (refs. [57, 58], version 2.26-r1175) with “-ax sr” for short read mode. The data were first run in paired end mode, and then run in single end mode, per human genome. Each successive run was converted from SAM to FASTQ using samtools [59] (version 1.17) with arguments “-f 12 -F 256 -N” for paired end data and “-f 4 -F 256” for single end. Single end data were repaired using fastq_pair [60] (version 1.0) specifying a table size of 50 M with “-t”. Compute support was provided with GNU Parallel [61] (version 20160222). Single-end FASTQ output from samtools was split into R1 and R2 with a custom Rust program, with rust-bio for parsing [62] (version 1.4.0). Data were multiplexed with GNU sed version 4.2.2 such that a unique sample identifier was added to each sequence record, and demultiplexed using a custom Python script.

RNA-Seq data, in addition to the aforementioned human filtering steps, were additionally filtered against the Gencode v44 database [63]. Specifically, a spliced minimap2 database was created from the Gencode v44 transcripts (https://ftp.ebi.ac.uk/pub/databases/gencode/Gencode_human/release_44/gencode.v44.transcripts.fa.gz), and the RNA-Seq data were mapped in paired end mode using the same parameters as upstream filtering.

### Simulating false positive human reads passing host depletion

For the SHOGUN-based simulation (Fig. 4H–J), the T2T-CHM13v2.0 human reference was used for generating human reads. Specifically, 10 random samples of one million pairs of reads each were generated (see parameters below) from T2T-CHM13v2.0, followed by BWA (v. 0.7.17) alignment against hg19 (GRCh37.p13) and extraction of unmapped reads. The BWA command was “bwa mem db fwd.fq rev.fq | samtools fastq -f 12 -F 256 -1 fwd.clean.fq -2 rev.clean.fq”. For the RS210-clean simulation (Supplementary Fig. 18E, F), ten HPRC genomes [38] were selected for human read generation: {HG002, HG00438, HG005, HG00621, HG00673, HG00733, HG00735, HG00741, HG01071, HG01106}. We simulated 1x coverage from each HPRC genome using ART [64] 3x times; maternal and paternal genomes were treated separately. For both simulations, the parameters provided to art_illumina were “-na -l 150 -m 270 -s 27 -f 1 -1 ART_MBARC-26_HiSeq_R1.txt -2 ART_MBARC-26_HiSeq_R2.txt”, where the read quality profiles (-1 and -2) used came from CAMI [65] (https://github.com/CAMI-challenge/CAMISIM/tree/master/tools/art_illumina-2.3.6/profiles). A random seed was provided with “-rs”, starting at 42 and incremented 1 per sample. One million paired end reads were then randomly sampled from each sample using the “sample” command from seqtk version 1.4-r122 (https://github.com/lh3/seqtk/). A random seed was provided with “-s” starting 42 and incrementing 1 per sample. For the SHOGUN-based simulation, unmapped reads after BWA-hg19 filtering were aligned against the originally published WoLr1 database [34]. For the RS210-clean simulations, the resulting sequences were filtered by GRCh38, GRCh38 + T2T-CHM13v2.0, or GRCh38 + T2T-CHM13v2.0 + pangenome; during filtering, the pangenomes used to generate simulated data were withheld.

### Database cleaning

Two complementary approaches were used to identify human reads in microbial databases: Exhaustive (this paper) and Conterminator [15]. Human sequences identified by either were masked in the respective microbial database genomes, as described below.

#### Exhaustive-based human sequence removal

Using a sliding window, all 150 bp sequences in steps of 75 bp were obtained from GRCh38, T2T-CHM13v2.0, and the human pangenome contigs. Any sequence with ≥100 N’s, or which was shorter than 75 bp (e.g., 3’ terminal positions), were omitted from subsequent use. Each sequence was then mapped against a given target database using the SHOGUN [66] bowtie2 (version 2.5.1) [67] parameter set. SAM [68] were subset using GNU awk version 4.0.2 to generate start and stop coordinates relative to the reference, specifically “awk -F’\t’ -v OFS = ‘\t’ ‘{sum = $4 + length($10); print $3, $4, sum, $1, $10}’”. This application is imperfect and can generate stop positions beyond the end of a contig; end positions exceeding the length of a contig were cut to the max length of the contig using a custom Python script. The SAM output were then sorted using GNU sort version 8.22 (“--parallel 8 --buffer-size = 100 g -k1,1 -k2,2n”) followed by line-based deduplication from a stream with a custom Python script. The deduplicated, sorted, mapping results were then merged into contiguous intervals using the merge action of bedtools [69] v2.31.0 using “-c 5 -o count”. A final fasta file of the contigs and regions were produced with bedtools getfasta action.

#### Conterminator human sequence removal for RS210, WoLr1, and custom Kraken database

Fasta files for microbial genomes used to construct the RefSeq version 210 database (29,648 genomes), WoLr1 database (10,575 genomes), or the original custom Kraken database (59,963 genomes) were processed with Conterminator [15] to identify all genomic regions shared with GRCh38 (ref. [36]), T2T-CHM13v2.0 (ref. [37]) or human pangenome [38] references. We clarify that the original Kraken database was first stated to have 59,974 genomes [1] but during this work was determined to have 59,963 genomes, related to a download error in 2016 that processed genome metadata but not the corresponding genome file. For Conterminator, the --kingdom parameter was set to “(2||2157||4751||10239),9606” to identify any human sequences shared by “bacteria OR archaea OR fungi OR viruses.” Conterminator outputs were processed to create fasta files containing the microbial genome ID, region, and sequence shared with any of the human references. For purposes of filtering the Kraken and Shogun data, the per-genome NCBI taxonomy was extracted and summarized at the genus-level, followed by intersecting all unique Conterminator-identified genera against the original abundance tables. This meant that genera were removed even if they contained a single contig in which a region was shared with a human sequence.

On examining the original Kraken database for human sequences, we did not observe any viruses detected by Conterminator despite running Conterminator as recommended specifying virus in the kingdom specification. In contrast, we observe a small number of viral genomes, including at least one known retrovirus, flagged as having overlapping regions with the human genome using Exhaustive. Differentiating contamination from integration is not in the scope of Exhaustive. However, as a practical matter these regions represent an ambiguity in differentiating a genomic source from a short read match. Additionally, for transparency, we attempted to run Conterminator as recommended on the original Kraken database’s viral subset alone, or with the viral data combined with other kingdoms. With viruses alone, we encountered a segmentation fault, where the output resembled failures that a Conterminator author suggested indicates a lack of contamination (see https://github.com/steineggerlab/conterminator/issues/12#issuecomment-771534666). When run in combination with other kingdoms, we did not observe any virus flagged as contamination, and the run completed without a segmentation fault. This inability to detect viruses was unexpected; nonetheless, we note that, per the Conterminator manuscript [15], the authors state they intentionally did not consider viruses in their work.

#### Base masking

For a given fasta representing a sequence database (e.g., RS210), subsequences between start and stop coordinates determined by Conterminator and the Exhaustive method were replaced with Ns. A Bowtie2 index was then constructed from the masked fasta.

### KrakenUniq pipeline

KrakenUniq version 1.0.4 (ref. [70]) was run on human filtered paired end samples with arguments “--report ${report} --db ${database} --threads ${threads} --paired --output off ${r1} ${r2}”, where the environment variables were substituted as needed at runtime. To improve resource utilization, groups of samples were processed serially such that the first sample in a group additionally specified the “--preload” argument. The database used was “KrakenUniq-MicrobialDB” downloaded on August 18, 2023 from https://benlangmead.github.io/aws-indexes/k2 and setup in accordance with the instructions.

### RefSeq210-clean pipeline

All human filtered short reads were mapped to the masked RS210 database with bowtie2 (v. 2.5.1) [67] using the SHOGUN [66] parameter set, specifically “-p ${threads} -x ${db} -q - --seed 42 --very-sensitive -k 16 --np 1 --mp “1,1” --rdg “0,1” --rfg “0,1” --score-min “L,0,-0.05” --no-head --no-unal” with environment variables set accordingly. To minimize overhead associated with the database load, samples were multiplexed such that each sequence ID was tagged with a unique sample identifier using GNU sed version 4.2.2, and demultiplexed with a custom Python program. The resulting SAM output was then processed with Woltka (version 0.1.5) [71] with the “classify” action and “-i ${sam} -o ${output} --no-demux --rank none” with environment variables set accordingly. Processing support was provided with GNU parallel version 20160222. The SAM output was additionally compressed for long term storage with xz 5.2.6. Individual Woltka tables were merged with “woltka_merge” from qp-woltka (https://github.com/qiita-spots/qp-woltka/blob/main/scripts/woltka_merge) which combines many feature tables using BIOM version 2.1.15’s Table.concat method [72].

### Calculating aggregate microbial coverages

The calculate_coverages.py script from Zebra [73] Filter (unversioned) was modified to emit per-sample coverage information in Python pickle files. Zebra Filter’s cover.py module was then modified to include an “add_ranges” method, such that pre-computed coverages could be added in. These modifications allowed for calculating genome coverage samples in parallel, and for aggregating coverage information across different sets of samples.

### Taxonomic filtering

TCGA lacked experimental contamination controls, precluding usage of blanks to infer true presence and biological likelihood of taxa in samples. Although *decontam* [74] was applied in our original work to filter, we later showed that its performance can be unideal in tumor or blood samples [49]. Thus, we developed a conservative, two step approach in this work: (i) enforce biological plausibility by restricting to human-associated taxa as much as possible, followed by (ii) removing false positives by microbial coverage metrics, either directly or by proxy. We realize that enforcing step (i) prevents observation of novel cancer-microbe associations and may be too restrictive for future studies, but nonetheless found it important for the context of this work.

For (i), we identified all species-level hits present in two large human body-site metagenome assemblies [42, 43], in an independent cancer cohort of decontaminated samples from the WIS of bacteria [2] and fungi [28], and among a comprehensive list of known pathogenic bacteria that infect humans dating to the 1800s [45]. We caveat that these species lists are biased towards bacteria, reliant on taxonomic naming that can vary over time, and do not include viruses or phages. The resultant table was filtered to unique species, which were extracted along with their unique genera, for the next stage of filtering. Since viruses and phages were excluded from these lists, and since we could not find a suitable equivalent describing which ones were human-associated, we more heavily relied on filtering them using step (ii), described below.

#### Additional KrakenUniq-specific filtering

The KrakenUniq paper states: “For the discovery of pathogens in human patients […] a read count threshold of 10 and unique k-mer count threshold of 1000 eliminated many background identifications while preserving all true positives, which were discovered from as few as 15 reads” [70]. We thus intersected the list of unique genera passing step (i) with those that had a read count ≥10 and a unique k-mer ≥1000 among TCGA samples to derive filtered non-viral genera. We then added human-associated (manually verified) viral genera that passed the same thresholds among TCGA samples. The combination of these steps provided the final list of 294 filtered KrakenUniq-MicrobialDB genera for downstream processing (Table S5).

#### Additional RS210-clean-specific filtering

Aggregated per-genome microbial coverages were calculated across TCGA using Zebra [73]. Genomes associated with unique non-viral species passing step (i) were filtered for having ≥50% aggregate genome coverage, followed by filtering viral genomes with the same threshold, leaving 689 unique species. *Cutibacterium acnes* and *Escherichia virus phiX174* were manually excluded from downstream analyses, and are not counted among these 689 species. Additional thresholds of ≥75% and ≥90% aggregate genome coverages were evaluated, finding 381 and 249 unique species, respectively. The set of 689 species with ≥50% aggregate genome coverages were used for downstream analyses (Table S7**)**.

### TCGA data splitting strategy

The principal variance component analysis by Poore and Kopylova et al. [1] revealed three main batch effect sources in the raw Kraken microbial data in TCGA: sequencing center (34.2% of total variance), sequencing platform (25.5% of total variance), and experimental strategy (36.2% of total variance). Fortunately, 16,087 of 17,625 quality-controlled TCGA samples were sequenced on a single instrument (Illumina HiSeq), and many sequencing centers focused on a single experimental strategy, including Harvard (WGS), Baylor (WGS), MD Anderson (WGS), Washington University (WGS), University of North Carolina (RNA-Seq), and Canada’s Michael Smith Genome Sciences Centre (RNA-Seq). Additionally, although the Broad Institute processed both WGS and RNA-Seq samples, among quality-controlled samples, only glioblastoma (GBM) samples were RNA-Seq, preventing across cancer ML comparisons; thus, only WGS data were used from the Broad Institute for downstream analyses. Other centers with fewer than 20 total samples were also excluded. This collectively provided 7 data batches in which raw data and normalized data subsets could be directly compared with minimal impact of batch effects.

### TCGA batch correction

#### Original batch corrected data

Voom-SNM-normalized data from the original manuscript was not re-computed, and details of how it was calculated are described therein [1].

#### ConQuR batch correction on originally published data

To compare Voom-SNM, which was not developed specifically for microbiome data, with a microbiome-specific supervised batch correction tool, we chose ConQuR, which performs non-parametric modeling to generate batch-removed zero-inflated read counts [14]. Importantly, ConQuR can only correct for one batch variable at a time, although it can accommodate multiple biological covariates. This limitation implied that it would not be possible to directly apply ConQuR to the entire TCGA dataset, which contained three large batch sources, and our empirical attempts to run ConQuR serially did not work well (data not shown). Thus, we decided to apply ConQuR to correct for sequencing center effects within WGS and RNA-Seq groups after subsetting to a single sequencing platform (Illumina HiSeq). ConQuR was run using default parameters, wherein the batch ID denoted the sequencing center and a single covariate comprised the TCGA sample type (e.g., primary tumor, blood); for the WGS group, the reference batch was “Harvard Medical School”, and for the RNA-Seq group, the “University of North Carolina” comprised the reference batch. We note that ConQuR can optionally tune over all available reference batches to find the ideal reference that most mitigates batch effects. After computing the ConQuR-normalized data on the WGS and RNA-Seq sample groups, we recomputed Voom-SNM-normalized data in an identical manner on each group. Specifically, Voom was run with quantile normalization followed by SNM with TCGA sample type (e.g., primary tumor, blood) as the biological variable and sequencing center as the technical factor for correction. PVCA was then performed on WGS and RNA-Seq groups separately using a percentage threshold parameter of 70% based on the NIEHS recommendation of 60–90% (https://www.niehs.nih.gov/research/resources/software/biostatistics/pvca/index.cfm). To make consistent comparisons, raw and ConQuR-corrected discrete count matrices were log-transformed with a pseudocount of 0.5 added since Voom-SNM outputs are already log-transformed.

#### ConQuR batch correction on KrakenUniq and RS210-clean data

For KrakenUniq and RS210-clean data (i.e., filtered taxa abundances), only Illumina HiSeq WGS samples were considered for ConQuR batch correction due to poor quality microbial read counts among the University of North Carolina samples (cf. Supplementary Fig. 21E, Supplementary Fig. 26E), leaving a single RNA-Seq center (Canada’s Michael Smith Genome Sciences Centre). ConQuR batch correction for KrakenUniq and RS210-clean data was calculated using default parameters while parallelizing across 32 cores and using “Baylor College of Medicine” as the WGS reference batch due to improvements in subsequent PVCA batch effect sizes (data not shown). Since only the discrete raw counts were being compared to the ConQuR-corrected counts, raw data were input into PVCA to evaluate the reduction in batch effect with a threshold parameter of 80%.

### Machine learning and feature comparisons

#### Two-class machine learning strategy

Machine learning was performed as detailed by Narunsky-Haziza et al. [28]. No hyperparameter optimization was used; all parameters were set prior to analysis, as follows: number of trees: 150, interaction depth: 3, shrinkage (learning rate): 0.1, number of minimally observed nodes: 1. Gradient boosting machine learning was applied with 10-fold cross validation, with AUROCs and precision recall (AUPRs) curves calculated on each holdout fold. Up/oversampling the minority class was used to correct for class imbalance, as recommended elsewhere [29]. Cancer types were compared in a one-type-versus-all-others manner for evaluating primary tumor samples and blood samples; alternatively, primary tumor versus adjacent normal comparisons were made with the concomitant samples. We required at least 20 samples per class in one-cancer-type-versus-all-other comparisons. Per-fold, per-cancer-type AUROCs and AUPRs were used to estimate confidence intervals. Model feature importances were saved based on the final cross-validated model.

#### Feature comparisons among machine learning models

All feature comparisons were consistently done within the same feature space (e.g., the 184 WIS-overlapping genera in the originally published data for Fig. 2E, F, H). When comparing features from the original paper [1] to RS210-clean, it was necessary to first intersect the features between each database to create a single, shared feature space. Specifically, all RS210-clean genomes with known human associations (i.e., species in UNITN, UHGG, WIS, or known pathogens; cf. Figure 5L) were intersected with the original Kraken database [1] at the genus-level (i.e., the taxonomic level previously examined [1]), identifying 428 unique overlapping genera. Once a single feature space was identified or established, additional testing could be done to compare downstream ML feature lists. Binary feature overlap was performed by first constructing 2×2 contingency tables based on intersecting two models’ feature importances to identify which features they both used and did not use (i.e., the diagonal values), and to identify which features were used by only one model in either direction (i.e., the off-diagonal values); features assigned an importance score of “0” were defined as not used by the model, and features with non-zero feature importances were defined as used by the model. Empirically, per-batch ML models typically used 50–150 features with non-zero feature importances; however, no explicit model regularization was implemented, and the models could theoretically use up to all available genera. Fisher exact tests were then calculated on the 2 × 2 contingency tables to identify the significance, or lack thereof, of the enrichment between the two feature lists. Kendall tau rank-based correlations were performed on the feature importances of each ML model, wherein features with zero-valued importance were assigned a rank of *k* + *1* where *k* denotes the number of features with non-zero importances, and features with non-zero importances were ranked according to their score. When Fisher exact tests and Kendall tau correlations were computed on a per-cancer-type basis within each batch, their output *p*-values were corrected across cancer types using Benjamini-Hochberg multiple hypothesis correction. When aggregating *p*-values across batches, the raw *p*-values from each batch were combined on a per-cancer-type basis using Fisher’s method, followed by Benjamini-Hochberg correction across the total number of aggregated cancer types. If a cancer type was represented in a single batch, then the raw *p*-value within that batch was included in the aggregated data, followed by Benjamini-Hochberg correction across the total number of aggregated cancer types. Since Fisher’s method effectively multiplies the *p*-values to combine them, and since the per-batch data consistently had significant results, the combined p-values are often very small. For plotting adjusted *p*-values, the negative logarithm base 10 was used; however, if a combined *p*-value was less than double xmin (2.2 × 10^−308^; see Statistical analyses section below), it was plotted as double xmin since the logarithm of 0 is infinity and could not be represented on the graph.

#### Multiclass machine learning

Gradient boosting machines were employed for multiclass machine learning with 10-fold cross validation and performed using xgboost [75]. No hyperparameter optimization was used; all parameters were set prior to analyses, as follows: nrounds = 10, max_depth = 4, eta = 0.1, gamma = 0, colsample_bytree = 0.7, min_child_weight = 1, subsample = 0.8. Up/oversampling the minority class was used to correct for class imbalance, as recommended elsewhere [29]. Concatenated predictions on holdout folds were used to calculate multiclass performance metrics, including mean balanced accuracy and average pairwise AUROCs. Multiclass confusion matrices can be colored in several ways. Since single gradient coloring is prone to give the false appearance that classes (e.g., cancer types) with few samples always have poor performance because they do not have strong shading along the diagonal, we separately shaded the diagonals to prevent such confusion in Fig. 3B–D, Fig. 6J, K, and Fig. 8J, K. Nonetheless, since some readers may prefer single gradient shading, we have also created concomitant versions of these confusion matrices in Supplementary Figs. 34, 35.

### Alpha and beta diversity analyses

Alpha and beta diversity were calculated on raw abundances from Illumina HiSeq samples.

#### Alpha diversity

Alpha diversity was calculated on rarefied per-batch, TCGA, KrakenUniq or RS210-clean filtered data using phyloseq [76]. Since RS210-clean data was at the genome level, and multiple genomes could exist for one species, counts were first aggregated to the species-level prior to alpha diversity calculations. Per-batch count data were rarefied to approximately the first quartile of the sample read distribution, or an absolute minimum of 25 reads, whichever was larger. Per-sample, per-cancer-type observed richness was determined and compared among cancer types within batches using non-parametric Kruskal-Wallis tests.

#### Beta diversity

Aitchison beta diversity analyses were calculated using RPCA [77] in QIIME 2 (ref. [78]) using default parameters except --p-min-sample-count was lowered to 100 for HMS, BCM, WashU, Broad, and CMS sequencing centers, and to 25 for MDA sequencing center. Subsequent PERMANOVAs were calculated with Qiime 2 (“qiime diversity adonis”) using 999 permutations and TCGA “investigation” (i.e., cancer type) as the target variable.

### Differential abundance analyses

Differential abundance was calculated on raw per-batch, TCGA, KrakenUniq or RS210-clean filtered data using ANCOM-BC [46] using each cancer type of interest versus all other cancer types in that respective batch. ANCOM-BC default parameters were used except for the following modifications: zero_cut = 0.999, p_adj_method = “BH”. We also required a minimum of 10 samples in each class (i.e., cancer type of interest, others). Output beta values, *p*-values, and BH-adjusted *q*-values were used to create volcano plots (e.g., Fig. 8H), such that positive log-fold changes denote microbes associated with the respective cancer type of interest.

### Statistical analyses

When ≥3 tests were performed, Benjamini-Hochberg multiple testing correction was performed to obtain adjusted *p*-values. Analyses and plots were generated with R version 4.1.1. Common R packages used include doMC (1.3.7), dplyr (v. 1.0.7), reshape2 (v. 1.4.4), ggpubr (0.4.0), ggsci (v. 2.9), rstatix (v. 0.7.0), tibble (v. 3.1.6), caret (v. 6.0-90), gbm (v. 2.1.8), xgboost (v. 1.5.0.1), MLmetrics (v. 1.1.1), PRROC (v. 1.3.1), pROC (v. 1.18.0), e1071 (v. 1.7–9), gmodels (v. 2.18.1), limma (v. 3.50.0), edgeR (v. 3.36.0), snm (v. 1.42.0), and sva (3.35.2). Fisher’s method for combining *p*-values was implemented using the survcomp (1.44.1) package’s combine.test function. The rstatix package corrected for multiple hypothesis testing where applicable. Sample sizes were not estimated in advance and power calculations were not performed. The gbm package was used for two-class ML; the xgboost package was used for multiclass gradient boosting ML. AUROC and AUPR were calculated using the PRROC package. We note that the R programming language has two numerical limits when it comes to calculating small numbers, including *p*-values: (1) double eps, or smallest positive floating-point number x such that 1 + x! = 1, which is 2.220446 × 10^−16^; (ii) double xmin, or the smallest non-zero normalized floating-point number, which is 2.225074 × 10^−308^ (although this limit may be even lower depending on the computing environment). Some R packages, notably ggpubr, do not report *p*-values less than double eps, so they are denoted in our data as *p* < 2.2 ×10^−16^; conversely, other R packages, notably rstatix, report *p*-values as low as double xmin, and any *p*-values less than double xmin in our data would be reported as *p* < 2.2 × 10^−308^. They are not a range of *p*-values.

### Supplementary information

Supplementary Text, Figures, & Table Legends

Supplementary Tables

## Data Availability

Re-analyses of the data from Gihawi et al. [13] are located here: https://github.com/gregpoore/tcga_rebuttal. Datasets too large to host on GitHub, including per-sample, host filtered TCGA FASTQ files may be shared upon reasonable request.

## Code availability

Analyses for this work are located here: https://github.com/gregpoore/MSR.
