# Correction: Robustness of cancer microbiome signals over a broad range of methodological variation

**DOI:** 10.1038/s41388-024-03018-z

**Published:** 2024-04-05

**Authors:** Gregory D. Sepich-Poore, Daniel McDonald, Evguenia Kopylova, Caitlin Guccione, Qiyun Zhu, George Austin, Carolina Carpenter, Serena Fraraccio, Stephen Wandro, Tomasz Kosciolek, Stefan Janssen, Jessica L. Metcalf, Se Jin Song, Jad Kanbar, Sandrine Miller-Montgomery, Robert Heaton, Rana Mckay, Sandip Pravin Patel, Austin D. Swafford, Tal Korem, Rob Knight

**Affiliations:** 1 Department of Bioengineering, University of California San Diego, La Jolla, CA USA; 2 Department of Pediatrics, University of California San Diego, La Jolla, CA USA; 3 Clarity Genomics, Antwerp, Belgium; 4 Department of Biomedical Informatics, Columbia University Irving Medical Center, New York, NY USA; 5 Program for Mathematical Genomics, Department of Systems Biology, Columbia University Irving Medical Center, New York, NY USA; 6 Present Address: Center for Microbiome Innovation, University of California San Diego, La Jolla, CA USA; 7 Department of Animal Sciences, Colorado State University, Fort Collins, CO USA; 8 Department of Medicine, University of California San Diego, La Jolla, CA USA; 9 Department of Psychiatry, University of California San Diego, La Jolla, CA USA; 10 Moores Cancer Center, University of California San Diego Health, La Jolla, CA USA; 11 Department of Obstetrics and Gynecology, Columbia University Irving Medical Center, New York, NY USA; 12 Department of Computer Science and Engineering, University of California San Diego, La Jolla, CA USA; 13 Present Address: Micronoma, San Diego, CA USA; 14 Present Address: Feinberg School of Medicine, Northwestern University, Chicago, IL USA; 15 Present Address: School of Life Sciences, Arizona State University, Tempe, AZ USA; 16 Present Address: Malopolska Centre of Biotechnology, Jagiellonian University in Kraków, Kraków, Poland; 17 Present Address: Algorithmic Bioinformatics, Department of Biology and Chemistry, Justus Liebig University Gießen, Gießen, Germany

**Keywords:** Microbiology, Genomics, Diagnostic markers

Correction to: *Oncogene* 10.1038/s41388-024-02974-w, published online 23 February 2024

Following publication of this article, an amendment was made to the Supplementary Text, Figures, & Table Legends document.

The original Supplementary Fig. 1B and the associated paragraph from the supplement were removed. To prevent figure reference renumbering, the authors separated the original Supplementary Fig. 1A into two panels A and B.

The new file is available via the link below:

10.1038/s41388-024-02974-w

### Supplementary information

Supplementary Information

