# Supplementary material for: Robustness of cancer microbiome signals over a broad range of methodological variation
Source: Oncogene. 2024 Feb 23;43(15):1127–48. doi: 10.1038/s41388-024-02974-w (PMC10997506; doi:10.1038/s41388-024-02974-w)
Supplement: Supplementary file 1 — Supplementary Text, Figures, & Table Legends [file 41388_2024_2974_MOESM1_ESM.pdf]

Supplementary Information

Robustness of cancer microbiome signals over a broad range of methodological variation

Gregory D. Sepich-Poore, Daniel McDonald, Evguenia Kopylova, Caitlin Guccione, Qiyun Zhu, George Austin, Carolina Carpenter, Serena Fraraccio, Stephen Wandro, Tomasz Kosciolek, Stefan Janssen, Jessica L. Metcalf, Se Jin Song, Jad Kanbar, Sandrine Miller-Montgomery, Robert Heaton, Rana Mckay, Sandip Pravin Patel, Austin D Swafford, Tal Korem, & Rob Knight

---

Table of contents

1. **Supplementary Text (p. 4)**
   - 1.1. Summary of analyses using original data and new data (p. 4)
   - 1.2. Two timelines for establishing context of this response (p. 5)
   - 1.3. Voom-SNM batch correction did not include cancer type information (p. 5)
   - 1.4. Subsetting strategy to compare uncorrected vs. VSNM-corrected data (p. 6)
   - 1.5. Negative control analyses comparing uncorrected vs. VSNM-corrected data (p. 6)
   - 1.6. Restricting analyses to raw WIS-overlapping genera abundances reproduces the original paper's conclusions (p. 6)
   - 1.7. Quotes of alleged database contamination by Gihawi et al. (p. 7)
   - 1.8. Database contamination with human sequences did not drive cancer microbiome classifiers when restricting to WIS-overlapping genera (p. 8)
   - 1.9. Estimating specificity of Exhaustive-pangenome host depletion pipeline (p. 8)
   - 1.10. Hundreds of human-associated species have well-covered genomes in TCGA blood and tumor samples (p. 9)
   - 1.11. Evaluating if ConQuR artifactually modifies the T2T-KrakenUniq-MicrobialDB or RS210-clean data to provide cancer type-specific microbiomes (p. 9)
   - 1.12. Top features and their biological plausibility (p. 10)
   - 1.13. Supplementary text references (p. 12)
2. **Supplementary Figures (p. 15)**
   - 2.1. Timeline providing context for this response (p. 15)
   - 2.2. Comparing raw and Voom-SNM (VSNM) data within batches does not reveal a systematic bias from batch correction among primary tumors (p. 16)
   - 2.3. Comparing raw and VSNM data within batches does not reveal a systematic bias from batch correction among blood samples or tumor versus normals (p. 18)
   - 2.4. Negative control analyses comparing raw and VSNM data does not reveal a systematic bias from batch correction among AUROCs across all cancer types and comparisons (p. 20)

2.5. Negative control analyses comparing raw and VSNM data does not reveal a systematic bias from batch correction among AUPRs across all cancer types and comparisons (p. 22)
2.6. ConQuR and VSNM methods provide similar levels of batch correction among TCGA sequencing centers, and comparing their normalized data to raw data subsets provides similar ML performances and feature similarities (p. 24)
2.7. Negative control analyses comparing raw, VSNM, and ConQuR WIS-overlapping data does not reveal a systematic bias from batch correction among AUROCs across all cancer types and comparisons (p. 26)
2.8. Negative control analyses comparing raw, VSNM, and ConQuR WIS-overlapping data does not reveal a systematic bias from batch correction among AUPRs across all cancer types and comparisons (p. 28)
2.9. VSNM-corrected, WIS-overlapping genera abundances demonstrate pan-cancer discrimination among blood and primary tumor samples across dozens of cancer types (p. 30)
2.10. Classifier performance using subset raw data with WIS-overlapping bacterial genera for cancer discrimination using primary tumor-derived microbial nucleic acids (p. 32)
2.11. WIS-overlapping raw data control analyses to verify TCGA primary tumor classifier performances (p. 34)
2.12. Classifier performance using subset raw data with WIS-overlapping bacterial genera for primary tumor versus adjacent tissue normal discrimination (p. 36)
2.13. WIS-overlapping raw data control analyses to verify TCGA primary tumor vs. adjacent normal classifier performances (p. 38)
2.14. Classifier performance using subset raw data with WIS-overlapping bacterial genera for cancer discrimination using blood-derived microbial nucleic acids (p. 40)
2.15. WIS-overlapping raw data control analyses to verify TCGA blood classifier performances (p. 42)
2.16. Removing genera with any human sequence contamination in the original databases does not impact downstream conclusions (p. 44)
2.17. Conservative removal of WIS-overlapping genera having any genome(s) with human sequences maintains cancer type-specific conclusions (p. 45)
2.18. Non-human read decreases in TCGA cancer types with successive host depletion and evaluation of Exhaustive versus Conterminator on WoLr1 (p. 47)
2.19. Impact of pangenome host depletion on KrakenUniq-MicrobialDB microbial read counts and high aggregate genome coverages of RS210-clean mapped species (p. 49)
2.20. Re-analysis of the data provided by Gihawi et al. [8] reveal cancer type-specific microbiomes (p. 51)
2.21. Filtered microbial reads per TCGA sequencing center using the T2T-KrakenUniq-MicrobialDB pipeline (p. 54)

2.22. Alpha diversity and machine learning (ML) reveals cancer type specific microbiomes in individual TCGA batches using KrakenUniq-MicrobialDB filtered genera (p. 55)
2.23. Aitchison beta diversity in individual TCGA batches using KrakenUniq-MicrobialDB filtered genera reveals cancer type specific microbiomes (p. 57)
2.24. Microbial differential abundances using KrakenUniq-MicrobialDB filtered genera abundances provide evidence for cancer type specific microbiomes (p. 58)
2.25. Evaluation of efficacy and impact of ConQuR batch correction using KrakenUniq-MicrobialDB-derived filtered genera (p. 60)
2.26. Filtered microbial reads per TCGA sequencing center using the SHOGUN/Woltka pipeline against RS210-clean (p. 62)
2.27. Alpha diversity and machine learning (ML) reveals cancer type specific microbiomes in individual TCGA batches using RS210-clean filtered species (p. 63)
2.28. Aitchison beta diversity in individual TCGA batches using RS210-clean filtered species reveals cancer type specific microbiomes (p. 65)
2.29. Microbial differential abundances using RS210-clean filtered species abundances provide evidence for cancer type specific microbiomes (p. 66)
2.30. Evaluation of efficacy and impact of ConQuR batch correction using RS210-clean-derived filtered species (p. 68)
2.31. Recommendations for each step of the cancer microbiome workflow (p. 70)
2.32. VSNM-corrected, full genera abundances demonstrate pan-cancer discrimination among blood and primary tumor samples across dozens of cancer types (p. 71)
2.33. ConQuR-corrected, full genera abundances demonstrate pan-cancer discrimination among blood and primary tumor samples across dozens of cancer types (p. 73)
2.34. Alternative version of Fig. 3 using a single color gradient for confusion matrices (p. 75)
2.35. Alternative versions of Fig. 6J-K and Fig. 8J-K using a single color gradient for confusion matrices (p. 76)

3. **Supplementary Table Legends (p. 77)**

Supplementary Text

1.1 Summary of analyses using original data and new data

To provide confidence in the originally published data, as well as demonstrate that newer methods reach the same conclusions, we (i) conducted re-analyses of the published data and (ii) added analyses on data updated with state-of-the-art techniques. These approaches are outlined below.

Regarding re-analyses of published data, we explicitly make direct machine learning (ML) comparisons between the raw and Voom-SNM (VSNM)-corrected data within every sequencing center batch, demonstrating equivalent performance and significantly similar features and rankings thereof within every ML model. We then use Conterminator [1] to identify and remove microbial genera having any associated genome with ≥1 human sequence mapping to GRCh38 [2], T2T-CHM13v2.0 [3], or human pangenome research consortium (HPRC) [4] references, finding minimal human contamination (≤1.08% of original microbial genomes in the databases) and equivalent downstream ML conclusions. As a conservative test, we repeat these analyses using only 184 Weizmann Institute of Science (WIS)-overlapping bacterial genera [5], again finding cancer type-specific results within the original raw data. With WIS-overlapping genera, we also demonstrate how a newer microbiome-specific batch correction method, ConQuR [6], provides equivalent ML performance to VSNM while more closely retaining per-batch microbial-cancer associations, albeit without being able to simultaneously correct for multiple batch variables.

For new methods, we sequentially deplete host reads from TCGA using GRCh38, T2T-CHM13v2.0, HPRC, and additionally human transcriptome (GENCODE [7]) references for RNA-Seq data, followed by serial application of the KrakenUniq-MicrobialDB pipeline described by Gihawi et al. [8], which contains a database with “human-cleaned” complete microbial genomes plus a human reference genome. We find that the number of input non-human reads following host depletion significantly correlates with the number of output microbial reads, even with no human contamination in the underlying database and only a single additional reference used for host depletion (i.e., host depletion with GRCh38 only versus with GRCh38+T2T-CHM12v2.0). These findings demonstrate that the degree of human depletion is the primary factor that drives the observed microbial read count rather than the inclusion of a human reference or human masking in the microbial databases. We then develop a new tool called “Exhaustive,” which is empirically twice as sensitive as Conterminator, for cleaning human sequences from RefSeq (v. 210) and finds significantly longer human sequences on identical microbial genomes. We then combine results from Exhaustive and Conterminator to derive a cleaned version of RefSeq v. 210 (“RS210-clean”), followed by direct alignments to it of TCGA reads fully host depleted by GRCh38, T2T-CHM13v2.0, HPRC, and GENCODE (RNA-seq only). We then filter taxonomic hits based on biological plausibility—species identified in large-scale human metagenomic assembly efforts [9,10], in independent WIS tumors [5,11], and known pathogenicity [12]—along with a minimal aggregate genome coverage of 50% [13], leaving 689 unique species for downstream analyses. Finally, using the KrakenUniq-MicrobialDB pipeline

described by Gihawi et al. [8] and these RefSeq results, we evaluate and find cancer type-specific microbiomes in tissues and blood using alpha diversity, beta diversity, differential abundance, and ML without or with ConQuR batch correction. Collectively, these systematic re-analyses of the original data and updated methods applied from scratch to the raw TCGA data once again substantiate the integrity of our original conclusions.

1.2 Two timelines for establishing context of this response

We present a timeline (**Supplementary Fig. 1A-B**) to provide context for the material and timing of this response. We first note that a careful reading of Gihawi et al. [8] reveals that most employed methods were published months to years after our original paper (**Supplementary Fig. 1A-B**), including the use of Conterminator [1], the "human-cleaned" microbial database ("MicrobialDB", released on "8/16/2020" per https://benlangmead.github.io/aws-indexes/k2), and the T2T-CHM13v1.1 human reference genome [14] utilized for host depletion prior to microbial mapping. Although the usage of future tools does not preclude critiques of former publications, it implies (i) that there is no reasonable justification that the original analysis could have included such methods, and (ii) that the primary implication of such critiques is whether the *conclusions* made by the original authors are still valid. This includes Voom-SNM, since no microbiome-specific batch correction tools contemporaneously existed that could account for the structure of TCGA data (e.g., percentile normalization [15] required cases and controls, and TCGA had no controls; ConQuR was not released until 2022 [6]) [16]. Stated more positively, if the application of future methods reveals that the original authors' conclusions remain intact (i.e., cancer type-specific microbiomes), then the robustness of those conclusions is indeed verified. Thus, in order to both provide confidence in the original methods and to verify that future methods demonstrate the robustness of the original findings, we conducted comprehensive re-analyses of the published data alongside new methods that were only recently developed.

1.3 Voom-SNM batch correction did not include cancer type information

As we stated in the original paper's methods, the Voom-SNM (VSNM) normalization did not contain cancer type information:

> "The Voom and SNM model matrices were equivalent and built using <u>sample type</u> as the target biological variable (n = 7; for example, primary tumour tissue) owing to expected biological differences between them [...] <u>It was not possible to model disease type as the target biological variable</u> owing to complete confounding between certain types of cancer and sequencing centres (that is, some types of cancer were only sequenced at one TCGA site)." [17]

This was an important choice because most TCGA patients had both tumor and normal (blood or tissue) sample types processed, often at the same sequencing center, meaning that "sample type" did not provide proxy information for cancer type either.

1.4 Subsetting strategy to compare uncorrected vs. VSNM-corrected data

To compare raw and VSNM data, we subset the samples to avoid interfering batch effects (**Fig. 1A**), which primarily comprised TCGA sequencing platform, sequencing center, and data type (WGS or RNA-Seq). We subset samples by these three batch variables because they collectively accounted for 95.9% of the original data variance (cf. Fig. 1e in original text [17]), thereby comprising the main batch effect sources. Fortunately, 91.27% of samples were sequenced on the Illumina HiSeq platform and most of the 7 sequencing centers focused on single data types. Of the 16,087 Illumina HiSeq samples, 15,739 (97.8%) were represented in these 7 batch subsets. Within each of the 7 batches, we then ran three ML comparisons using either the subset raw or Voom-SNM data: primary tumor-based one cancer type versus all others, blood-based one cancer type versus all others, and tumor versus normal. Finally, for each batch and comparison, we compared raw-versus-VSNM AUROCs and AUPRs, Fisher exact tests based on binary overlap of the genera used by each ML model, and Kendall tau correlations based on the relative feature importance rankings (**Fig. 1B-G, Supplementary Fig. 2-3, Methods**). For ease of plotting, we also aggregated the results across all of the 7 batches, using Fisher's method to combine p-values from the Fisher exact tests and Kendall tau correlations in a per-cancer type manner, with subsequent multiple testing correction among cancer types (**Fig. 1J-K, M**).

1.5 Negative control analyses comparing uncorrected vs. VSNM-corrected data

We sought to confirm that both raw and VSNM data would equivalently respond to negative control analyses, and thus repeated all ML models using scrambled metadata labels or shuffled counts (**Supplementary Fig. 4-5**). Under shuffling or scrambling, predictive signals would indicate leakage of test labels in the model. These negative control analyses revealed substantial, significant reductions in AUROCs (**Supplementary Fig. 4**) and AUPRs (**Supplementary Fig. 5**) compared to actual data across all cancer types. Aggregating the data in this manner also revealed no clear trend among the raw and VSNM data performances: no significant difference among tumor vs. normal AUROCs or AUPRs (**Supplementary Fig. 4F, 5F**), slightly but not significantly higher VSNM data performance among blood samples (**Supplementary Fig. 4E, 5E**), and slightly but not significantly higher raw data performance among primary tumors (**Supplementary Fig. 4D, 5D**). Thus, we conclude that VSNM did not systematically bias the data to enable cancer type discrimination, because equivalent ML model performances and significantly similar feature rankings are obtained using the raw data, as well as equivalent negative control responses.

1.6 Restricting analyses to WIS-overlapping genera reproduces the original paper's conclusions

Gihawi et al. [8] cite WIS-overlapping bacteria within their own work [18] and cancer microbiome patent application [19], signaling their acceptance of these taxa, so we investigated whether our published raw abundances of these taxa could replicate the original conclusion of cancer type-specific microbiomes. After subsetting TCGA samples to single sequencing centers,

sequencing platform (Illumina HiSeq), and data types, we performed per-batch ML within and between cancer types. Doing so revealed that, in all raw data subsets, primary tumors were always distinguishable using WIS-overlapping bacteria (**Supplementary Fig. 10**). Implementing scrambled and shuffled ML control analyses revealed significantly better performance for the non-shuffled and non-scrambled samples in every raw data subset (**Supplementary Fig. 11**). We continued this pattern for tumor versus normal tissues using raw data subsets, finding that every model outperformed (**Supplementary Fig. 12**) its null expected values, with the sole exception of lung adenocarcinoma at Harvard Medical School (**Supplementary Fig. 12A**). Still, when aggregating cancer types in each sequencing center, the raw data subsets performed significantly better than matched scrambled or shuffled count models (**Supplementary Fig. 13**). In the same manner, repeating the blood-related ML models using raw data subsets consistently showed strong performances that were always better than the null expected values (**Supplementary Fig. 14**). This was complemented by these models showing significantly better performances than scrambled or shuffled counterparts (**Supplementary Fig. 15)**. Thus, conservative analyses that restrict the raw data to decontaminated taxa from an independent, decontaminated cohort provide the same cancer type-specific conclusions whether using raw data, VSNM (**Supplementary Fig. 9**), or ConQuR (**Fig. 3**) batch correction.

1.7 Quotes of alleged database contamination by Gihawi et al.

In numerous places, Gihawi et al. [8] allege that the original microbial database was highly contaminated with human sequences:

1. "errors in the genome database [...] led to millions of false-positive findings of bacterial reads across all samples, largely because most of the sequences identified as bacteria were instead human"
2. "Database contamination can, in turn, lead to misclassification of human reads that match a contaminated non-human genome"
3. "i.e., reads that were reported to match a bacterial genome when in fact the reads were from human DNA"
4. "the vast majority of the excessive counts in the Poore et al. study were apparently due to human reads in the filtered data that were incorrectly labeled as bacterial"
5. "the vast majority of these over-counts are human reads that were erroneously assigned to bacteria"
6. "[Section title] How human reads create the false appearance of bacteria"
7. "The likely reason for these vast over-counts is that human reads were erroneously categorized as bacterial by Poore et al. "
8. "The number of human reads matching bacteria was unrelated to the actual presence of bacteria in the tumor sample; instead, it was determined by the database itself, in which many draft bacterial genomes contained mislabeled human sequences."
9. "Thus, the Kraken matches were nearly all false positives, caused by the presence in the database of bacterial genomes that erroneously contained human sequences."
10. "The likely cause of these overestimates was that the metagenomics database included thousands of draft genomes, which are known to be contaminated with human

sequences. Consequently, as we showed above, millions of human reads were erroneously assigned to bacterial or archaeal genera”

1.8 Database contamination with human sequences did not drive cancer microbiome classifiers when restricting to WIS-overlapping genera

As a conservative measure, we repeated the comparison of raw versus Conterminator-filtered genera after subsetting the published Kraken data to WIS-overlapping bacterial genera [5], followed by removing all genera having any associated genome detected by Conterminator to share ≥1 human sequence against GRCh38, T2T-CHM13v2.0, or human pangenome references (**Supplementary Fig. 17A, top**). Using the remaining 125 genera versus the original 184 overlapping genera, we applied the same data splitting strategy to compare per-batch ML performances among the raw and filtered data subsets (**Supplementary Fig. 17A, bottom**). Aggregated AUROCs were equivalent across all blood-based comparisons, across 93.8% (30 of 32; LUSC and PCPG excluded) of primary tumor comparisons, and across 91.7% (11 of 12; COAD excluded) of tumor versus normal comparisons (**Supplementary Fig. 17B, D, F**). Similarly, aggregated AUPRs were equivalent across all blood-based comparisons, across 84.4% (27 of 32; ACC, DLBC, LUSC, PCPG, THYM excluded) of primary tumor comparisons, and across 91.7% (11 of 12; COAD excluded) of tumor versus normal comparisons (**Supplementary Fig. 17C, E, G**). Nonetheless, these exceptions still had average AUROCs exceeding 89% and used just 6.3% of the total features identified in original work. Thus, we conclude that highly conservative analyses that restrict the raw data to taxa from an independent, highly-decontaminated cohort at the WIS, and whose genomes lacked human sequences still provide cancer type-specific microbiome conclusions.

1.9 Estimating specificity of Exhaustive-pangenome host depletion pipeline

Because database cleaning is complementary to, but not a substitute for, host depletion, we quantitatively estimated how many reads would remain after sequentially increasing levels of host depletion (**Supplementary Fig. 18E-F**). Specifically, we held out 5 male and 5 female HPRC reference genomes to simulate paired-end 150 base pair Illumina reads at 1x coverage, followed by subsampling to one million reads per sample three times and performing sequential host depletion with hg38, T2T-CHM13v2.0, and independent HPRC references, calculating how many reads passed each step (**Supplementary Fig. 18E; Methods**). Notably, sequential application of all three host depletion tiers left 0 reads in 60% of the held out HPRC samples, and <15 reads in the remaining samples (**Supplementary Fig. 18F**). Of the 44 sequences remaining out of 60 million total reads simulated, a single sequence (forward read only) aligned to four genomes: *Parabacteroides merdae* (GCF_004166975.1), *Blautia faecis* (GCF_013302415.1), *Eubacterium tenue* (GCF_015668515.1), and *Actinomyces oris* (GCF_016127955.1). The sequence (“TTACTTTGCCAACCATTCGATAACTGTTAAGTAGACATGTATATATTGCACTGGCTATTCATCT TGCACTTTTCCTCTTTCTTCTTCCCAGTAGCCTCATCCTTTTACGCTGCCTCTCTGGAACTTG CCATCATCATTCCCTAGAAACTG”) aligns to many *Saccharomyces cerevisiae* with MegaBLAST [20] against the nucleotide collection (nr/nt), but does not align when limited to

human (taxid: 9606) against the nucleotide collection (nr/nt), RefSeq reference genomes, or RefSeq genomes, reporting “No significant similarity found”. Searching for “somewhat similar sequences” using BLASTN [21], limited to human (taxid: 9606) against the nucleotide collection (nr/nt), yields two hits with 23% query coverage and 91.43% sequence identity. These data suggest that full host depletion alone would eliminate most false positives in addition to using a cleaned reference database, although we recommend using both together in practice. We further advocate for continued methods development, and expansion of databases suitable for removal of human reads, and stress that human read removal is not a solved problem.

1.10 Hundreds of human-associated species have well-covered genomes in TCGA blood and tumor samples

To obtain further insight into the relative contribution of TCGA sample types to RS210-clean microbes, we re-computed genome coverages using sample type subsets, including only with primary tumors (PT), blood derived normal (BDN) samples, or solid tissue normal (STN) samples (**Fig. 5M**). Notably, we find that dozens of non-viral species had nearly complete genome coverages from blood samples alone, including numerous orally-derived bacteria such as *Streptococcus mutans* (98.1%), *Prevotella denticola* (97.6%), *Rothia denticariosa* (97.3%), *Actinomyces naeslundii* (97.1%), *Scardovia wiggsiae* (95%), and *Porphyromonas endodontalis* (94.6%) among others (**Supplementary Fig. 19C**). Deconvolving coverage origins further revealed that *Fusobacterium nucleatum* had nearly complete coverage within each sample subset (PT: 99.7%, BDN: 96.5%, STN: 97.5%; **Fig. 5M, inset**), including blood, supporting recent data that it spreads to tumors hematogenously [22,23]. Repeating these non-viral coverage calculations by WGS or RNA-Seq supersets also revealed significant correlation (Spearman rho: 0.74; $p<2.2\times10^{-16}$; **Supplementary Fig. 19D**), either due to sufficient flow-through during polyA-enrichment and/or bacterial mRNA polyadenylation, which, although less frequent than mammalian mRNA polyadenylation, does occur [24–26]. Thus, even after applying rigorous human depletion and database cleaning, we find that hundreds of human-associated microbial species have substantial portions of their genomes covered in TCGA (**Table S5-S6)**.

1.11 Evaluating if ConQuR artifactually modifies the T2T-KrakenUniq-MicrobialDB or RS210-clean data to provide cancer type-specific microbiomes

To test whether ConQuR artifactually modified the KrakenUniq-MicrobialDB data or features to enhance cancer type diagnosis, we performed a head-to-head comparison against the raw data by subsetting both among every individual batch, followed by performing within-batch ML (**Supplementary Fig. 25A, top**). We then compared ML performances, as well as feature similarity within every ML model by Fisher exact and Kendall tau tests, for ConQuR versus raw data for every individual cancer type (**Supplementary Fig. 25A, bottom**). Per-cancer-type AUROC and AUPRs were aggregated across all batches to construct confidence intervals, and p-values of feature comparisons were aggregated using Fisher’s method (**Methods**). Importantly, all primary tumor and blood comparisons were equivalent or better using the raw data (**Supplementary Fig. 25C, G**). Some tumor versus normal comparisons lacked agreement

(**Supplementary Fig. 25D**), with significantly better ConQuR-corrected performances, potentially due to sample type being used as the biological variable during supervised batch correction (**Methods**), which may make these batch corrected comparisons less reliable. Feature comparisons revealed a similar pattern, with significantly similar ConQuR-versus-raw data cancer microbiome signatures for primary tumor and blood comparisons for every cancer type (**Supplementary Fig. 25E, H**), but weaker or nonsignificant similarity for tumor versus normal comparisons (**Supplementary Fig. 25F**).

We then repeated the same data splitting strategy with the RS210-clean, ConQuR-corrected data as done with the KrakenUniq-MicrobialDB data, verifying that ConQuR did not artifactually create cancer type-specific conclusions in either ML performance or feature similarities, although we continue to caution about the limitations of the ConQuR-corrected tumor versus normal models (**Supplementary Fig. 30C-H**). Thus, we conclude that ConQuR did not artifactually enhance the data for primary tumor or blood comparisons and simultaneously find that microbial signatures can accurately discriminate between dozens of cancer types (**Fig. 6J-K, Fig. 8J-K**) using either the critiquing authors' preferred bioinformatic pipeline or a direct alignment method.

1.12 Top features and their biological plausibility

All non-viral taxa in the KrakenUniq and RS210-clean pipelines were required to have known human associations (and all required ≥50% genomic coverage for RS210-clean or read-based filters for KrakenUniq, see **Methods**) prior to downstream analyses by overlapping with at least one of the following databases: UNITN [9], UHGG [10], WIS [5,11], or known pathogens [12]. This was done to ensure their biological plausibility upfront given the sensitivity of this work, although in the future this constraint could be relaxed to enable detection of novel cancer-microbe associations.

We have provided the feature lists in **Table S15-S18** while carefully noting the following limitations:

1. By virtue of how the TCGA Consortium designed their cohorts, there is no bona fide "control" group of healthy individuals to which one can compare cancer types. Instead, an internal control of "normal" tissue, either adjacent solid tissue or blood, was collected on a per patient basis. This unfortunately prevents comparisons of each TCGA cancer type to a consistent, single control group. Thus, in both the 2020 paper and this work, we used "all other cancer types" as a proxy control group.
2. Feature importances do not indicate if a particular feature was differentially abundant or indicate in which group that feature was more or less abundant. For transparency, we previously tried to emphasize this on the website for the 2020 paper data:

    > "Moreover, a high feature importance score for a given taxon does *not* guarantee or imply an overabundance of that taxon for this comparison. A high feature importance score only means that the taxon was important for making predictions; whether the taxon is less or more abundant in

certain samples requires statistical testing.” (taken from http://cancermicrobiome.ucsd.edu/)

3. The combination of #1 and #2 above means that ranked features could be associated with either the cancer type of interest or those in the control group (i.e., “all other cancer types”). Although it is anticipated that highly ranked features correspond to the cancer type of interest, it is not guaranteed, and this necessitates caution when interpreting top ranked features for per-cancer type feature lists, even if the overall conclusion is clear that cancer types differ. We therefore also performed statistical testing in this work with ANCOM-BC [27], which was published after our original 2020 paper, to test whether each taxon is associated with the cancer type of interest.
4. Using the recommended R210-clean pipeline with enforced human associations of taxa (cf. **Fig. 8**), nearly all of the TCGA WGS samples are maintained, but many of the RNA-Seq samples do not have sufficient read counts for subsequent analyses (**Fig. 8B**), likely due to their lower read depth (**Fig. 8C**) and/or polyA-enrichment. This reduced the number of samples available for ML, including some of the cancer types; for example, adrenocortical carcinoma (ACC) primary tumors only had RNA-Seq samples (n=79) available for the 2020 paper, and not enough of these samples had sufficient read depths to analyze ACC in the RS210-clean pipeline (cf. **Fig. 8J**). Additionally, since ConQuR can only correct for one batch variable at a time, the RS210-clean+ConQuR pipeline corrected for sequencing center bias but could not also correct for WGS vs. RNA-Seq biases, requiring us to keep these data types separate in the ML analyses (e.g., **Fig. 6J-K**, **Fig. 8J-K**). These two factors—RNA-Seq sample drop out and sequencing center-only batch correction—preclude doing the ML in an equivalent manner to the 2020 paper, as the control group (i.e., “all other cancer types”) is not equivalent, and highly ranked features can be associated with those cancer types in the control group.
5. Because a primary goal of this work was to show that cancer types remain differentiable by human-associated microbes, and since no healthy control group was/is available in TCGA, we focused on effectively detecting tissue of origin (TOO) by computing multiclass ML models (**Fig. 3**, **Fig. 6J-K**, **Fig. 8J-K**, **Supplementary Fig. 9**). Each of these multiclass ML models provide a single list of taxa that are useful for simultaneously discriminating between cancer types, and they better reflect real-world diagnostic scenarios when the TOO is unknown and needs to be identified to determine therapeutic management (e.g., see GRAIL’s TOO efforts in Figure 6 of [28]).

Taking into account the above considerations and limitations, the taxa feature lists and importance of each taxon derived from the multiclass ML models in **Table S15-S18** correspond to **Fig. 3**, **Fig. 6J-K**, **Fig. 8J-K**, and **Supplementary Fig. 9**. For the KrakenUniq and RS210-clean model lists, we have clearly noted the overlap of each non-viral species (or genera) with the human associated databases used for initial filtering (UNITN, UHGG, WIS, known pathogens), and their genomic coverages when possible, to demonstrate their biological plausibility. To derive per-cancer type lists of associated taxa—wherein the results clearly indicate strength and directionality of the association rather than just predictive capacity—we

performed differential testing using ANCOM-BC [27] on the ConQuR-corrected, KrakenUniq and RS210-clean data among primary tumors and blood samples (**Table S19-S20**).

1.13 Supplementary text references

1. Steinegger M, Salzberg SL. Terminating contamination: large-scale search identifies more than 2,000,000 contaminated entries in GenBank. Genome Biol. 2020;21: 115. doi:10.1186/s13059-020-02023-1

2. Schneider VA, Graves-Lindsay T, Howe K, Bouk N, Chen H-C, Kitts PA, et al. Evaluation of GRCh38 and de novo haploid genome assemblies demonstrates the enduring quality of the reference assembly. Genome Res. 2017;27: 849–864. doi:10.1101/gr.213611.116

3. Rhie A, Nurk S, Cechova M, Hoyt SJ, Taylor DJ, Altemose N, et al. The complete sequence of a human Y chromosome. Nature. 2023;621: 344–354. doi:10.1038/s41586-023-06457-y

4. Liao W-W, Asri M, Ebler J, Doerr D, Haukness M, Hickey G, et al. A draft human pangenome reference. Nature. 2023;617: 312–324. doi:10.1038/s41586-023-05896-x

5. Nejman D, Livyatan I, Fuks G, Gavert N, Zwang Y, Geller LT, et al. The human tumor microbiome is composed of tumor type-specific intracellular bacteria. Science. 2020;368: 973–980. doi:10.1126/science.aay9189

6. Ling W, Lu J, Zhao N, Lulla A, Plantinga AM, Fu W, et al. Batch effects removal for microbiome data via conditional quantile regression. Nat Commun. 2022;13: 5418. doi:10.1038/s41467-022-33071-9

7. Frankish A, Diekhans M, Ferreira A-M, Johnson R, Jungreis I, Loveland J, et al. GENCODE reference annotation for the human and mouse genomes. Nucleic Acids Res. 2019;47: D766–D773. doi:10.1093/nar/gky955

8. Gihawi A, Ge Y, Lu J, Puiu D, Xu A, Cooper CS, et al. Major data analysis errors invalidate cancer microbiome findings. MBio. 2023; e0160723. doi:10.1128/mbio.01607-23

9. Pasolli E, Asnicar F, Manara S, Zolfo M, Karcher N, Armanini F, et al. Extensive Unexplored Human Microbiome Diversity Revealed by Over 150,000 Genomes from Metagenomes Spanning Age, Geography, and Lifestyle. Cell. 2019;176: 649–662.e20. doi:10.1016/j.cell.2019.01.001

10. Almeida A, Nayfach S, Boland M, Strozzi F, Beracochea M, Shi ZJ, et al. A unified catalog of 204,938 reference genomes from the human gut microbiome. Nat Biotechnol. 2021;39: 105–114. doi:10.1038/s41587-020-0603-3

11. Narunsky-Haziza L, Sepich-Poore GD, Livyatan I, Asraf O, Martino C, Nejman D, et al. Pan-cancer analyses reveal cancer type-specific fungal ecologies and bacteriome interactions. Cell. 2022;185: 3789–3806.e17. doi:10.1016/j.cell.2022.09.005

12. Bartlett A, Padfield D, Lear L, Bendall R, Vos M. A comprehensive list of bacterial pathogens infecting humans. Microbiology. 2022;168. doi:10.1099/mic.0.001269

13. Hakim D, Wandro S, Zengler K, Zaramela LS, Nowinski B, Swafford A, et al. Zebra: Static and Dynamic Genome Cover Thresholds with Overlapping References. mSystems. 2022;

e0075822. doi:10.1128/msystems.00758-22

14. Nurk S, Koren S, Rhie A, Rautiainen M, Bzikadze AV, Mikheenko A, et al. The complete sequence of a human genome. Science. 2022;376: 44–53. doi:10.1126/science.abj6987

15. Gibbons SM, Duvallet C, Alm EJ. Correcting for batch effects in case-control microbiome studies. PLoS Comput Biol. 2018;14: e1006102. doi:10.1371/journal.pcbi.1006102

16. Wang Y, LêCao K-A. Managing batch effects in microbiome data. Brief Bioinform. 2020;21: 1954–1970. doi:10.1093/bib/bbz105

17. Poore GD, Kopylova E, Zhu Q, Carpenter C, Fraraccio S, Wandro S, et al. Microbiome analyses of blood and tissues suggest cancer diagnostic approach. Nature. 2020;579: 567–574. doi:10.1038/s41586-020-2095-1

18. Hurst R, Meader E, Gihawi A, Rallapalli G, Clark J, Kay GL, et al. Microbiomes of Urine and the Prostate Are Linked to Human Prostate Cancer Risk Groups. Eur Urol Oncol. 2022;5: 412–419. doi:10.1016/j.euo.2022.03.006

19. Cooper CS, Brewer DS, Hurst R, Gihawi AFS, O'grady JJ, Wain JR, et al. Tumour microbiome analysis as a prognostic indicator of prostate cancer. World Patent. 2023139137:A1, 2023. Available: https://patentimages.storage.googleapis.com/43/cc/91/086a59a621f9af/WO2023139137A1.pdf

20. Morgulis A, Coulouris G, Raytselis Y, Madden TL, Agarwala R, Schäffer AA. Database indexing for production MegaBLAST searches. Bioinformatics. 2008;24: 1757–1764. doi:10.1093/bioinformatics/btn322

21. Altschul SF, Gish W, Miller W, Myers EW, Lipman DJ. Basic local alignment search tool. J Mol Biol. 1990;215: 403–410. doi:10.1016/S0022-2836(05)80360-2

22. Parhi L, Alon-Maimon T, Sol A, Nejman D, Shhadeh A, Fainsod-Levi T, et al. Breast cancer colonization by Fusobacterium nucleatum accelerates tumor growth and metastatic progression. Nat Commun. 2020;11: 3259. doi:10.1038/s41467-020-16967-2

23. Abed J, Maalouf N, Manson AL, Earl AM, Parhi L, Emgård JEM, et al. Colon Cancer-Associated Fusobacterium nucleatum May Originate From the Oral Cavity and Reach Colon Tumors via the Circulatory System. Front Cell Infect Microbiol. 2020;10: 400. doi:10.3389/fcimb.2020.00400

24. Sarkar N. Polyadenylation of mRNA in prokaryotes. Annu Rev Biochem. 1997;66: 173–197. doi:10.1146/annurev.biochem.66.1.173

25. Hajnsdorf E, Kaberdin VR. RNA polyadenylation and its consequences in prokaryotes. Philos Trans R Soc Lond B Biol Sci. 2018;373. doi:10.1098/rstb.2018.0166

26. Mohanty BK, Kushner SR. Bacterial/archaeal/organellar polyadenylation. Wiley Interdiscip Rev RNA. 2011;2: 256–276. doi:10.1002/wrna.51

27. Lin H, Peddada SD. Analysis of compositions of microbiomes with bias correction. Nat Commun. 2020;11: 3514. doi:10.1038/s41467-020-17041-7

28. Liu MC, Oxnard GR, Klein EA, Swanton C, Seiden MV, Liu MC, et al. Sensitive and specific multi-cancer detection and localization using methylation signatures in cell-free DNA. Ann Oncol. 2020;31: 745–759. doi:10.1016/j.annonc.2020.02.011

29. Rivera-Pinto J, Egozcue JJ, Pawlowsky-Glahn V, Paredes R, Noguera-Julian M, Calle ML. Balances: a New Perspective for Microbiome Analysis. mSystems. 2018;3. doi:10.1128/mSystems.00053-18

30. Morton JT, Marotz C, Washburne A, Silverman J, Zaramela LS, Edlund A, et al. Establishing microbial composition measurement standards with reference frames. Nat Commun. 2019;10: 2719. doi:10.1038/s41467-019-10656-5

31. Martino C, Morton JT, Marotz CA, Thompson LR, Tripathi A, Knight R, et al. A Novel Sparse Compositional Technique Reveals Microbial Perturbations. mSystems. 2019;4. doi:10.1128/mSystems.00016-19

Supplementary Figures

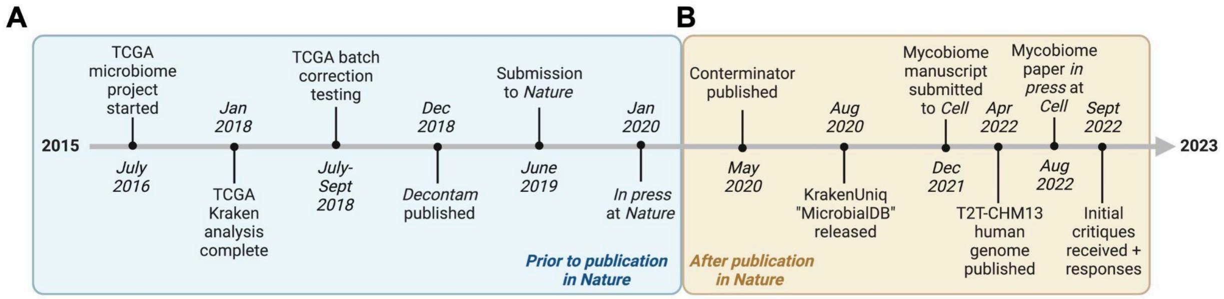

C

Disease Type	TCGA Abbreviation
Adrenocortical Carcinoma	ACC
Acute Myeloid Leukemia	LAML
Bladder Urothelial Carcinoma	BLCA
Brain Lower Grade Glioma	LGG
Breast Invasive Carcinoma	BRCA
Cervical Squamous Cell Carcinoma and Endocervical Adenocarcinoma	CESC
Cholangiocarcinoma	CHOL
Colon Adenocarcinoma	COAD
Esophageal Carcinoma	ESCA
Glioblastoma Multiforme	GBM
Head and Neck Squamous Cell Carcinoma	HNSC
Kidney Chromophobe	KICH
Kidney Renal Clear Cell Carcinoma	KIRC
Kidney Renal Papillary Cell Carcinoma	KIRP
Liver Hepatocellular Carcinoma	LIHC
Lung Adenocarcinoma	LUAD
Lung Squamous Cell Carcinoma	LUSC

Disease Type	TCGA Abbreviation
Lymphoid Neoplasm Diffuse Large B-cell Lymphoma	DLBC
Mesothelioma	MESO
Ovarian Serous Cystadenocarcinoma	OV
Pancreatic Adenocarcinoma	PAAD
Pheochromocytoma and Paraganglioma	PCPG
Prostate Adenocarcinoma	PRAD
Rectum Adenocarcinoma	READ
Sarcoma	SARC
Skin Cutaneous Melanoma	SKCM
Stomach Adenocarcinoma	STAD
Testicular Germ Cell Tumors	TGCT
Thymoma	THYM
Thyroid Carcinoma	THCA
Uterine Carcinosarcoma	UCS
Uterine Corpus Endometrial Carcinoma	UCEC
Uveal Melanoma	UVM

Supplementary Figure 1. Timeline providing context for this response. (A-B) Key software and project development milestones before (**A**) and after (**B**) our original manuscript was published. **(C)** List of TCGA cancer type abbreviations.

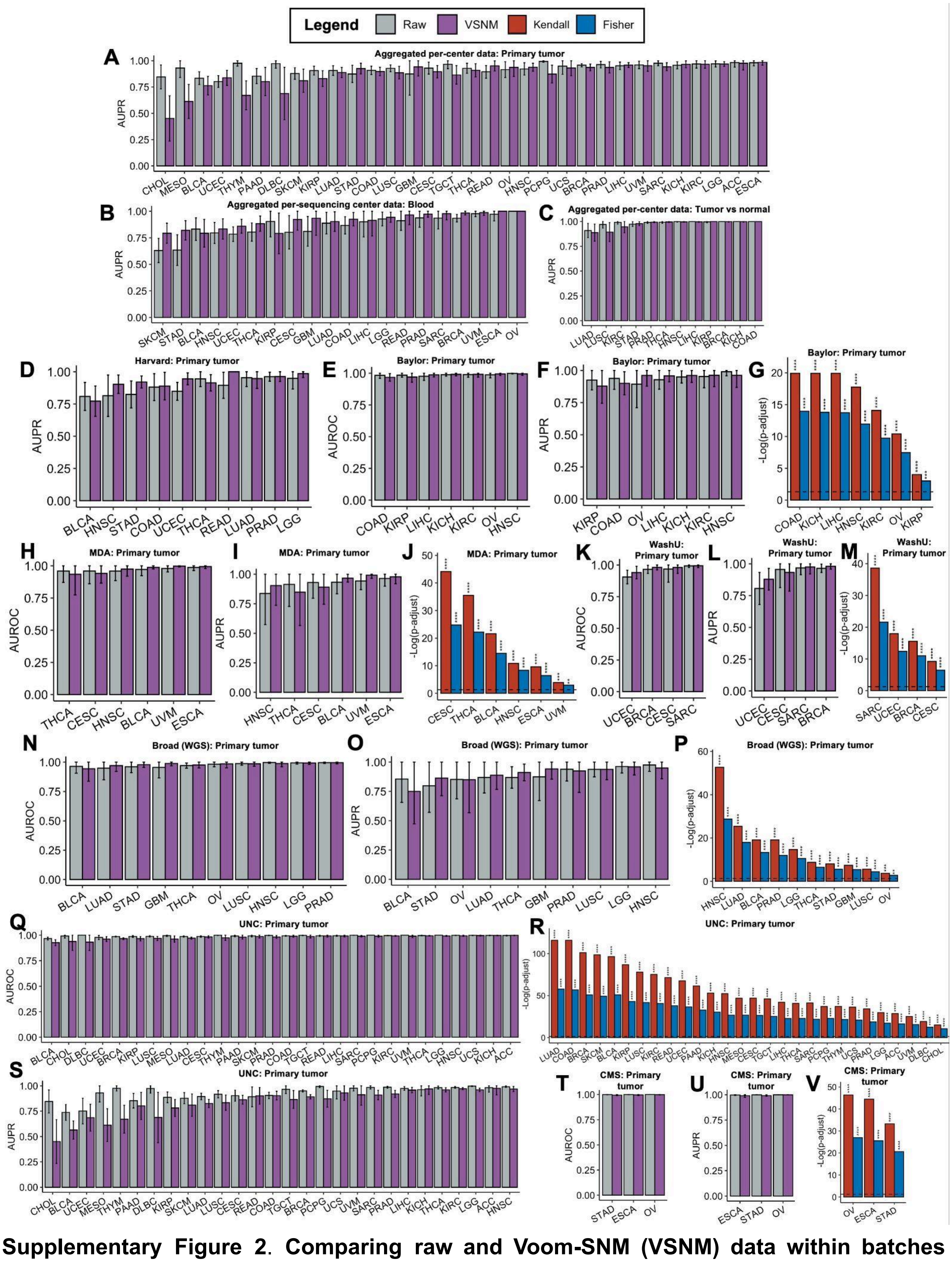

Supplementary Figure 2. **Comparing raw and Voom-SNM (VSNM) data within batches does not reveal a systematic bias from batch correction among primary tumors**. **(A-C)**

Aggregated AUPR data across all per-batch **(A)** primary tumor and **(B)** blood sample, and **(C)** tumor versus normal comparisons using the raw and VSNM data. **(D)** Raw-versus-VSNM AUPRs from comparing cancer types using primary tumors in Harvard Medical School. **(E-F)** Raw-versus-VSNM **(E)** AUROCs, **(F)** AUPRs, and **(G)** feature similarities from comparing cancer types using primary tumors in Baylor College of Medicine. **(H-J)** Raw-versus-VSNM **(H)** AUROCs, **(I)** AUPRs, and **(J)** feature similarities from comparing cancer types using primary tumors in MD Anderson. **(K-M)** Raw-versus-VSNM **(K)** AUROCs, **(L)** AUPRs, and **(M)** feature similarities from comparing cancer types using primary tumors in Washington University. **(N-P)** Raw-versus-VSNM **(N)** AUROCs, **(O)** AUPRs, and **(P)** feature similarities from comparing cancer types using primary tumors in the Broad Institute's WGS samples. **(Q-S)** Raw-versus-VSNM **(Q)** AUROCs, **(R)** feature similarities, and **(S)** AUPRs from comparing cancer types using primary tumors in the University of North Carolina. **(T-V)** Raw-versus-VSNM **(T)** AUROCs, **(U)** AUPRs, and **(V)** feature similarities from comparing cancer types using primary tumors in the Canada's Michael Smith Genome Sciences Centre. **(A-F, H-I, K-L, N-O, Q, S-U)** Error bars denote 99% confidence intervals. **(G, J, M, P, R, V)** Kendall tau correlations are shown in red. Fisher exact tests are shown in blue. Logarithms are base 10.

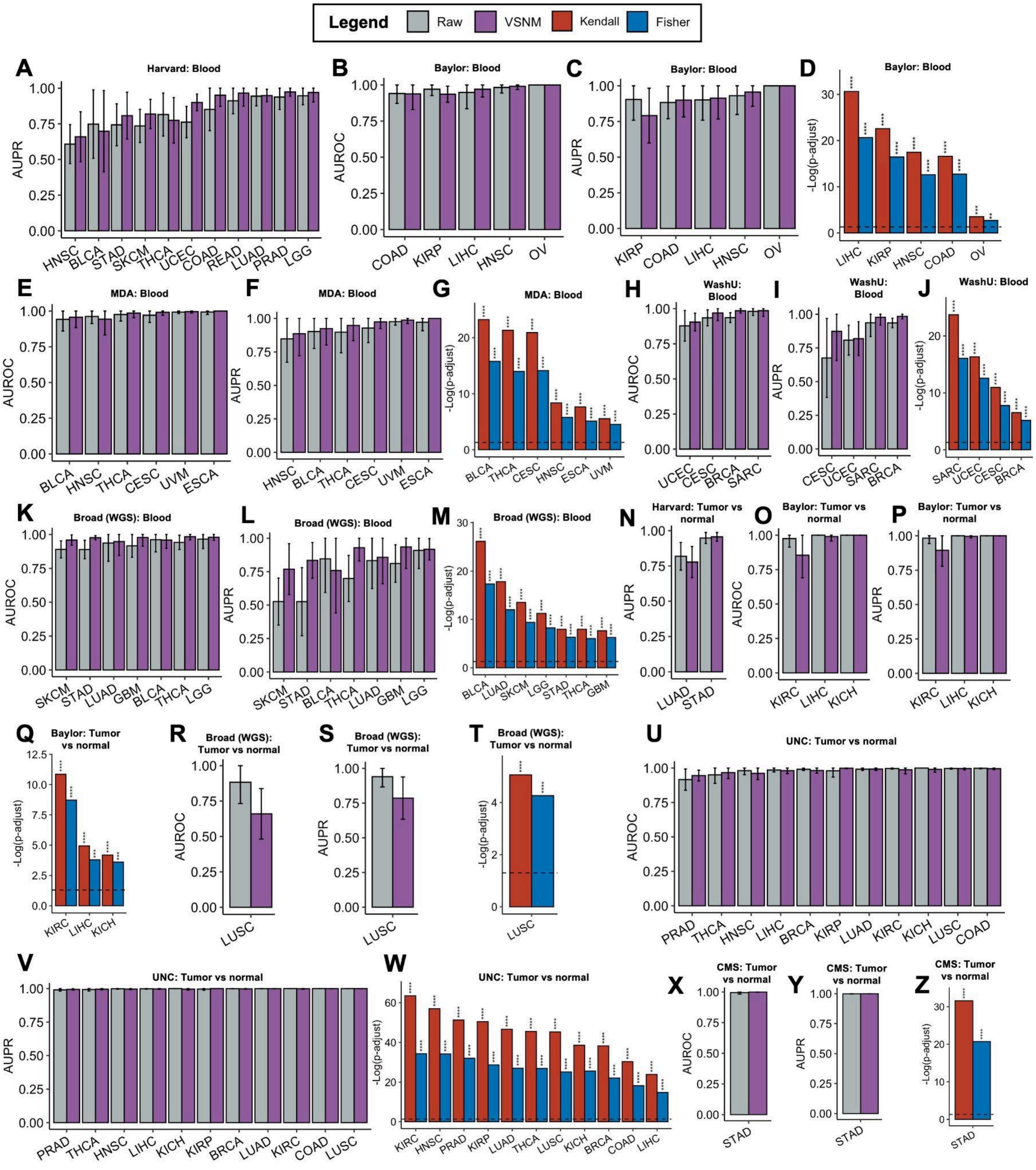

Supplementary Figure 3. Comparing raw and VSNM data within batches does not reveal a systematic bias from batch correction among blood samples or tumor versus normals. **(A)** Raw-versus-VSNM AUPRs from comparing cancer types using blood samples in Harvard Medical School. **(B-D)** Raw-versus-VSNM **(E)** AUROCs, **(F)** AUPRs, and **(G)** feature similarities from comparing cancer types using blood samples in Baylor College of Medicine. **(E-G)** Raw-versus-VSNM **(E)** AUROCs, **(F)** AUPRs, and **(G)** feature similarities from comparing cancer types using primary tumors in MD Anderson. **(H-J)** Raw-versus-VSNM **(H)** AUROCs, **(I)**

AUPRs, and **(J)** feature similarities from comparing cancer types using primary tumors in Washington University. **(K-M)** Raw-versus-VSNM **(K)** AUROCs, **(L)** AUPRs, and **(M)** feature similarities from comparing cancer types using primary tumors in the Broad Institute's WGS samples. **(N)** Raw-versus-VSNM AUPRs from comparing tumor versus normal samples in Harvard Medical School. **(O-Q)** Raw-versus-VSNM **(O)** AUROCs, **(P)** AUPRs, and **(Q)** feature similarities from comparing tumor versus normal samples in Baylor College of Medicine. **(R-T)** Raw-versus-VSNM **(R)** AUROCs, **(S)** AUPRs, and **(T)** feature similarities from comparing tumor versus normal in the Broad Institute's WGS samples. **(U-W)** Raw-versus-VSNM **(U)** AUROCs, **(V)** AUPRs, and **(W)** feature similarities from comparing tumor versus normal samples in the University of North Carolina. **(X-Z)** Raw-versus-VSNM **(X)** AUROCs, **(Y)** AUPRs, and **(Z)** feature similarities from comparing tumor versus normal samples in Canada's Michael Smith Genome Sciences Centre. **(A-C, E-F, H-I, K-L, N-P, R-S, U-V, X-Y)** Error bars denote 99% confidence intervals. **(D, G, J, M, Q, T, W, Z)** Kendall tau correlations are shown in red. Fisher exact tests are shown in blue. Logarithms are base 10.

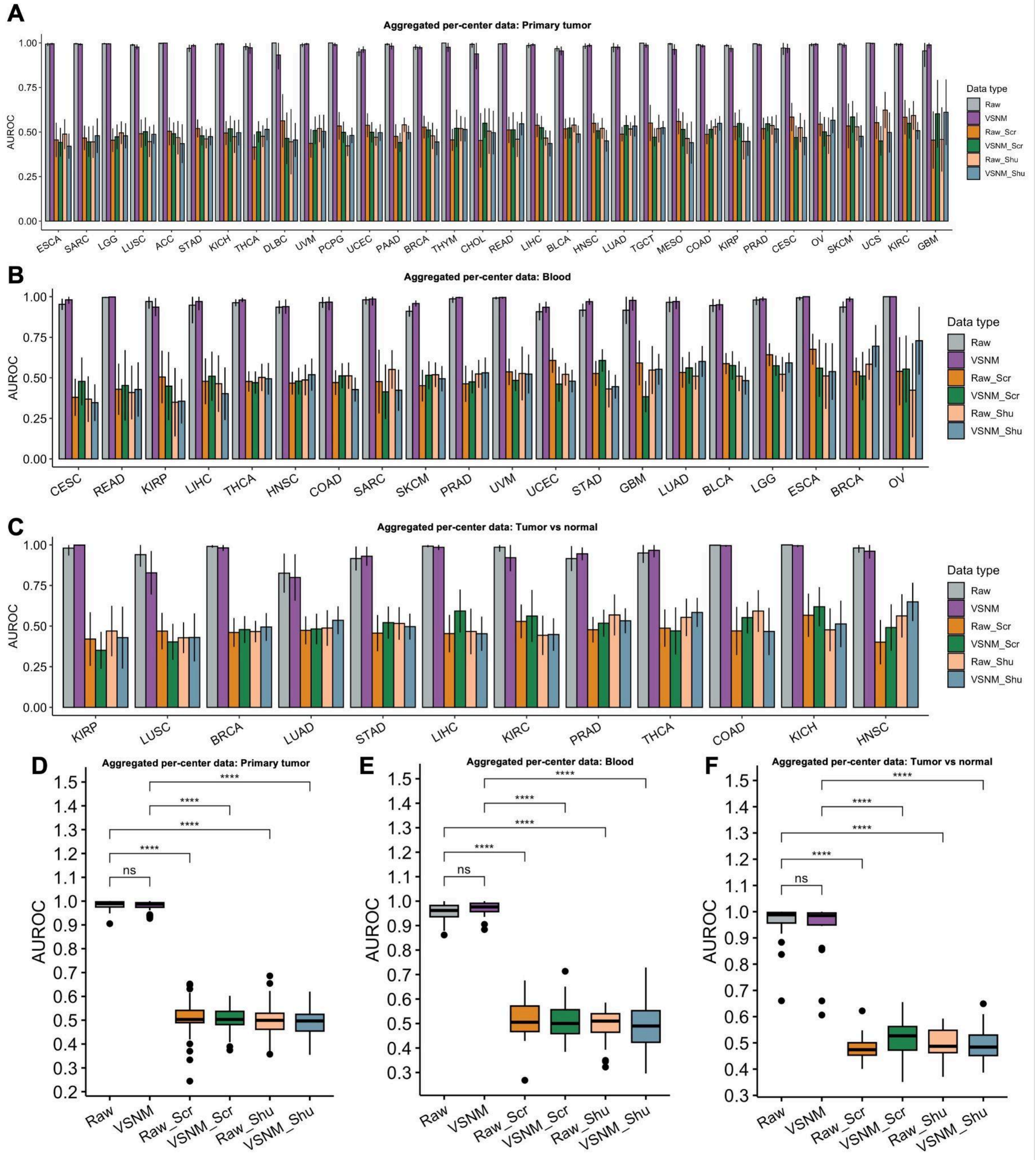

Supplementary Figure 4. Negative control analyses comparing raw and VSNM data does not reveal a systematic bias from batch correction among AUROCs across all cancer types and comparisons. **(A-C)** Aggregated AUROC data across all per-batch **(A)** primary tumor, **(B)** blood sample, and **(C)** tumor versus normal comparisons using the raw and VSNM data for each cancer type. Identical ML models were run while scrambling metadata labels or shuffling feature counts for each data type. Error bars denote 99% confidence intervals. **(D-F)** Per-cancer and per-batch AUROCs were averaged across their cross-validation folds and

compared among **(D)** primary tumor, **(E)** blood samples, and **(F)** tumor versus normal to calculate two-sided Wilcoxon tests with Benjamini-Hochberg correction.

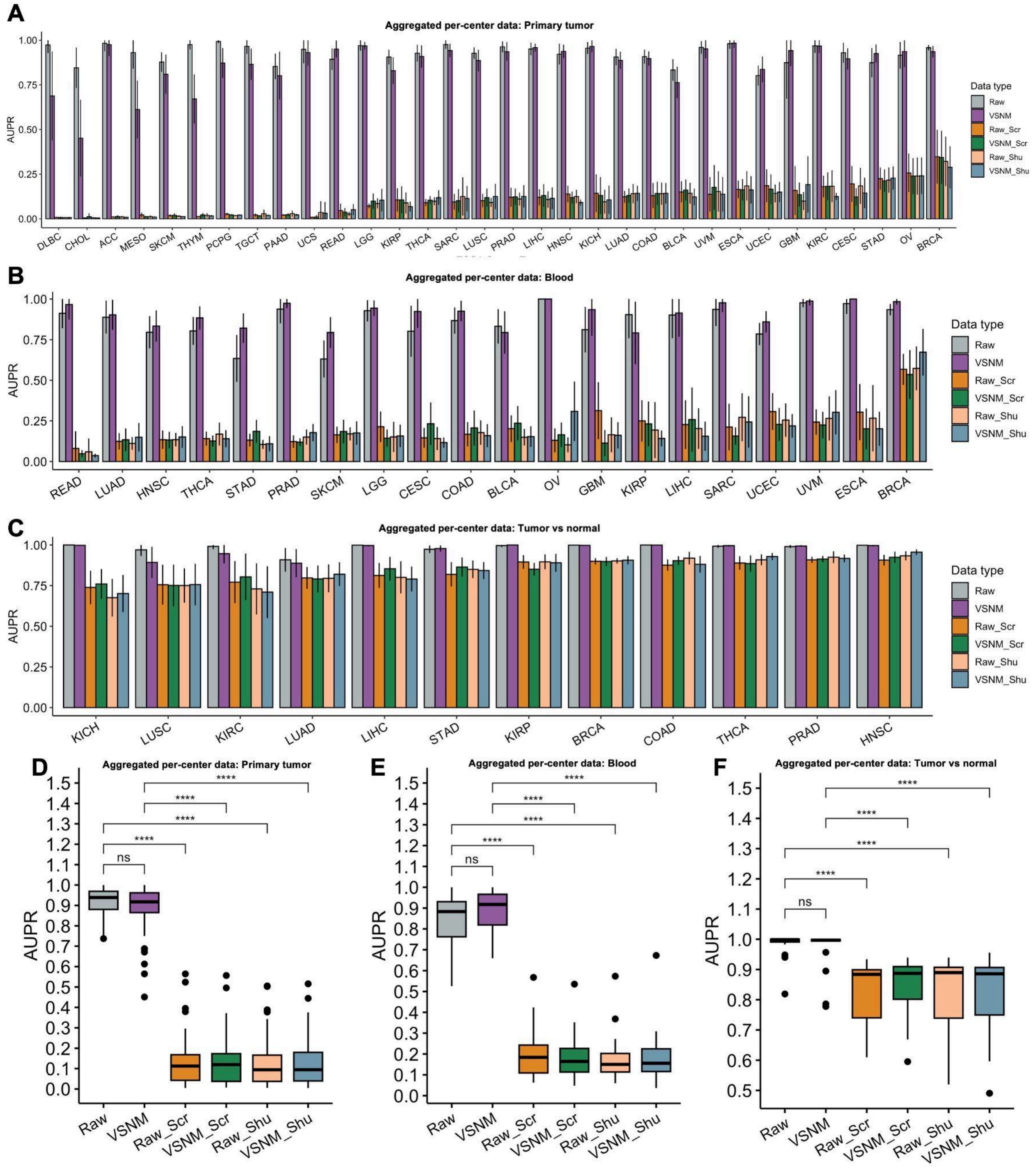

Supplementary Figure 5. **Negative control analyses comparing raw and VSNM data does not reveal a systematic bias from batch correction among AUPRs across all cancer types and comparisons**. **(A-C)** Aggregated AUPR data across all per-batch **(A)** primary tumor, **(B)** blood sample, and **(C)** tumor versus normal comparisons using the raw and VSNM data for each cancer type. Identical ML models were run while scrambling metadata labels or shuffling

feature counts for each data type. Error bars denote 99% confidence intervals. **(D-F)** Per-cancer and per-batch AUROCs were averaged across their cross-validation folds and compared among **(D)** primary tumor, **(E)** blood samples, and **(F)** tumor versus normal to calculate two-sided Wilcoxon tests with Benjamini-Hochberg correction.

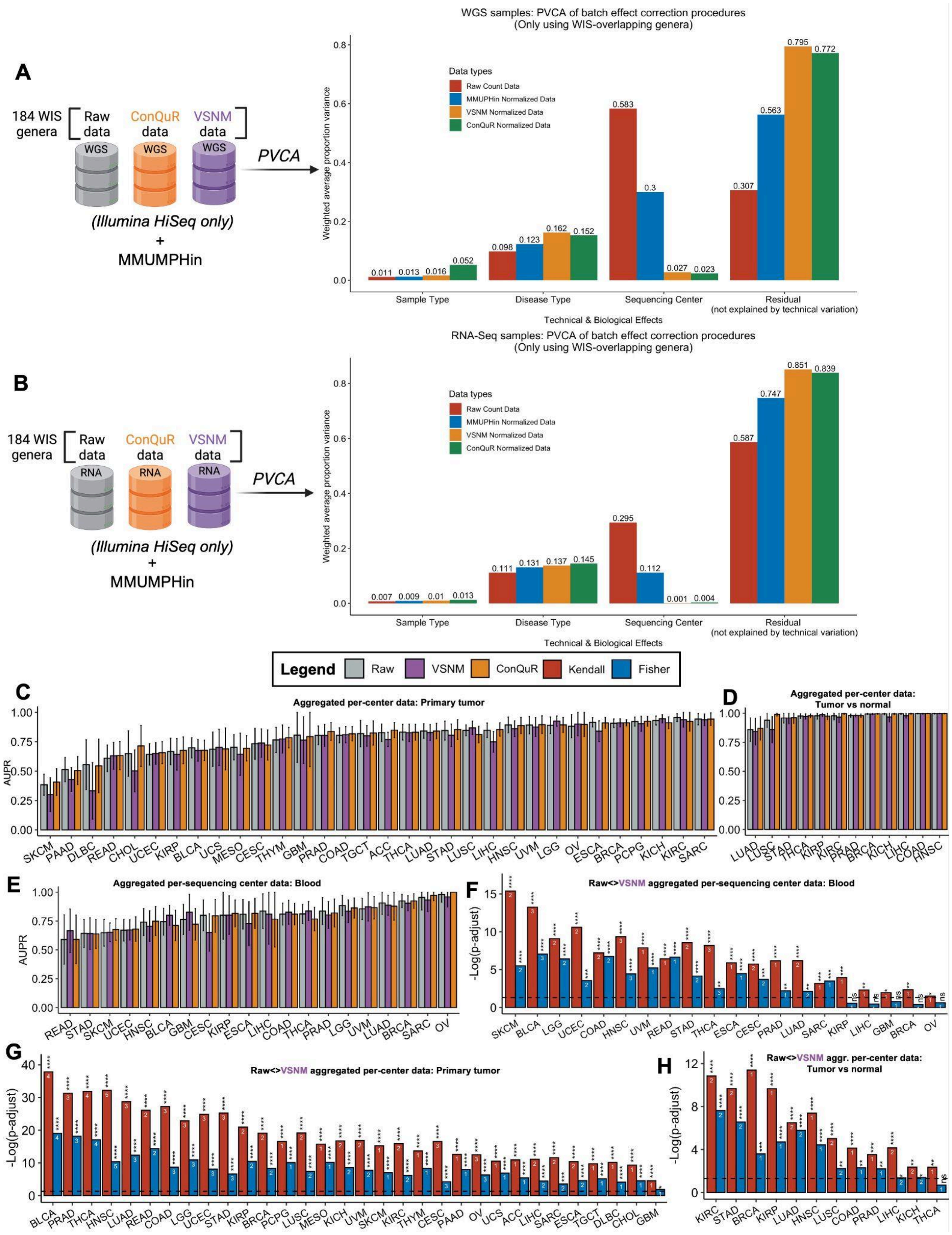

Supplementary Figure 6. ConQuR and VSNM methods provide similar levels of batch correction among TCGA sequencing centers, and comparing their normalized data to raw

data subsets provides similar ML performances and feature similarities. (A) Principal variance components analysis (PVCA) for sequencing center effects among the raw, ConQuR-normalized, or VSNM-normalized WIS-overlapping, WGS-only data. **(B)** Principal variance components analysis for sequencing center effects among the raw, ConQuR-normalized, or VSNM-normalized WIS-overlapping, RNA-Seq-only data. **(C-E)** Aggregated AUPR data across all per-batch **(C)** primary tumor **(D)** tumor versus normal, and **(E)** blood sample comparisons. Error bars denote 99% confidence intervals. **(F-H)** Aggregated and combined p-values from VSNM-versus-raw, per-batch Fisher exact tests (blue) and Kendall tau correlations (red) across all per-batch **(F)** blood, **(G)** primary tumor, and **(H)** tumor versus normal comparisons. Inset white numbers denote the number of batches (i.e., sequencing centers) from which data derived for each particular cancer type. P-values were combined across multiple batches using Fisher's method on the raw per-batch p-values, followed by Benjamini-Hochberg correction across cancer types. Logarithms are base 10.

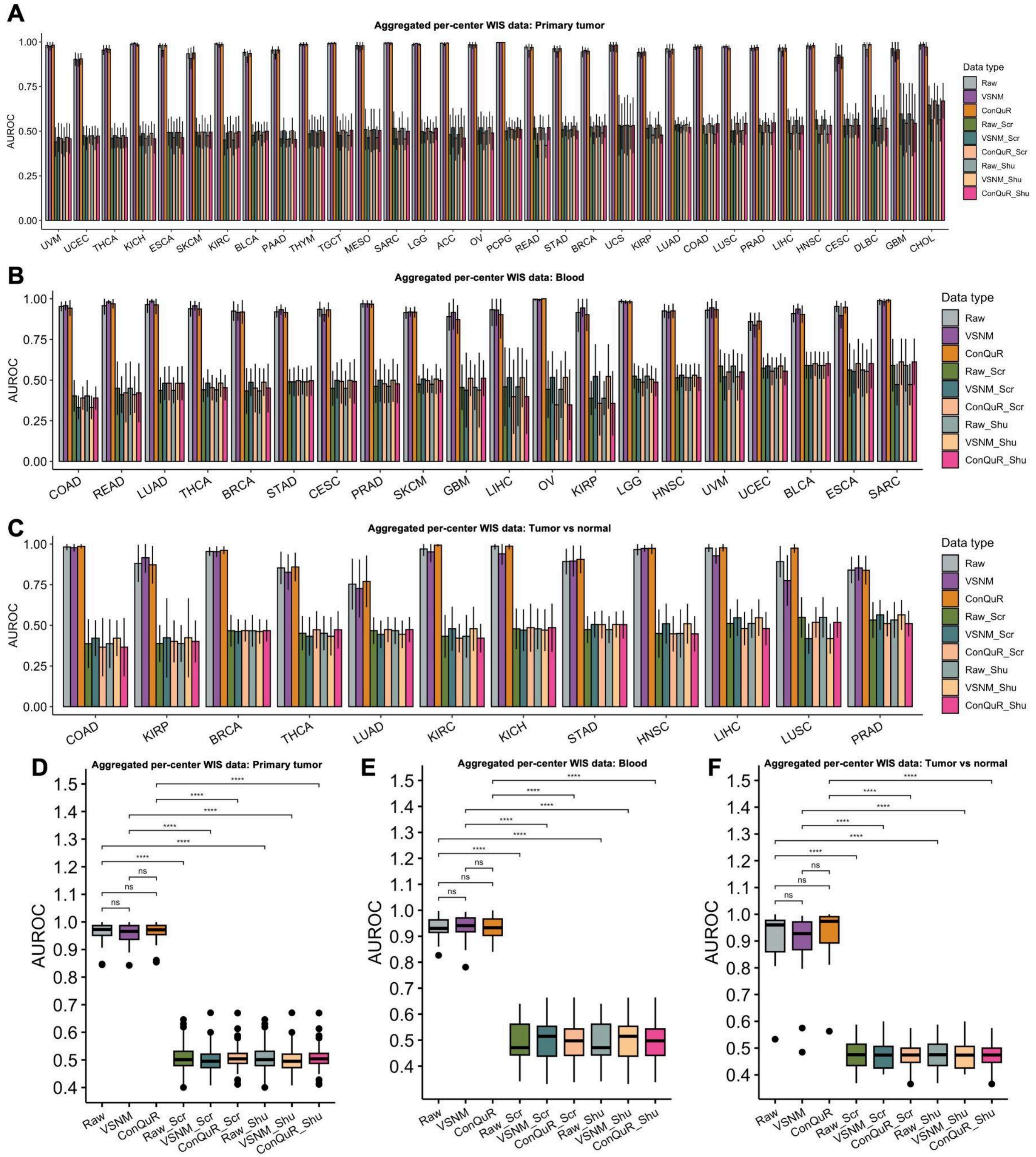

Supplementary Figure 7. Negative control analyses comparing raw, VSNM, and ConQuR WIS-overlapping data does not reveal a systematic bias from batch correction among AUROCs across all cancer types and comparisons. **(A-C)** Aggregated AUROC data across all per-batch **(A)** primary tumor, **(B)** blood sample, and **(C)** tumor versus normal comparisons using the raw, VSNM, and ConQuR WIS-overlapping data for each cancer type. Identical ML models were run while scrambling metadata labels or shuffling feature counts for each data type. Error bars denote 99% confidence intervals. **(D-F)** Per-cancer and per-batch AUROCs were averaged across their cross-validation folds and compared among **(D)** primary tumor, **(E)**

blood samples, and **(F)** tumor versus normal to calculate two-sided Wilcoxon tests with Benjamini-Hochberg correction.

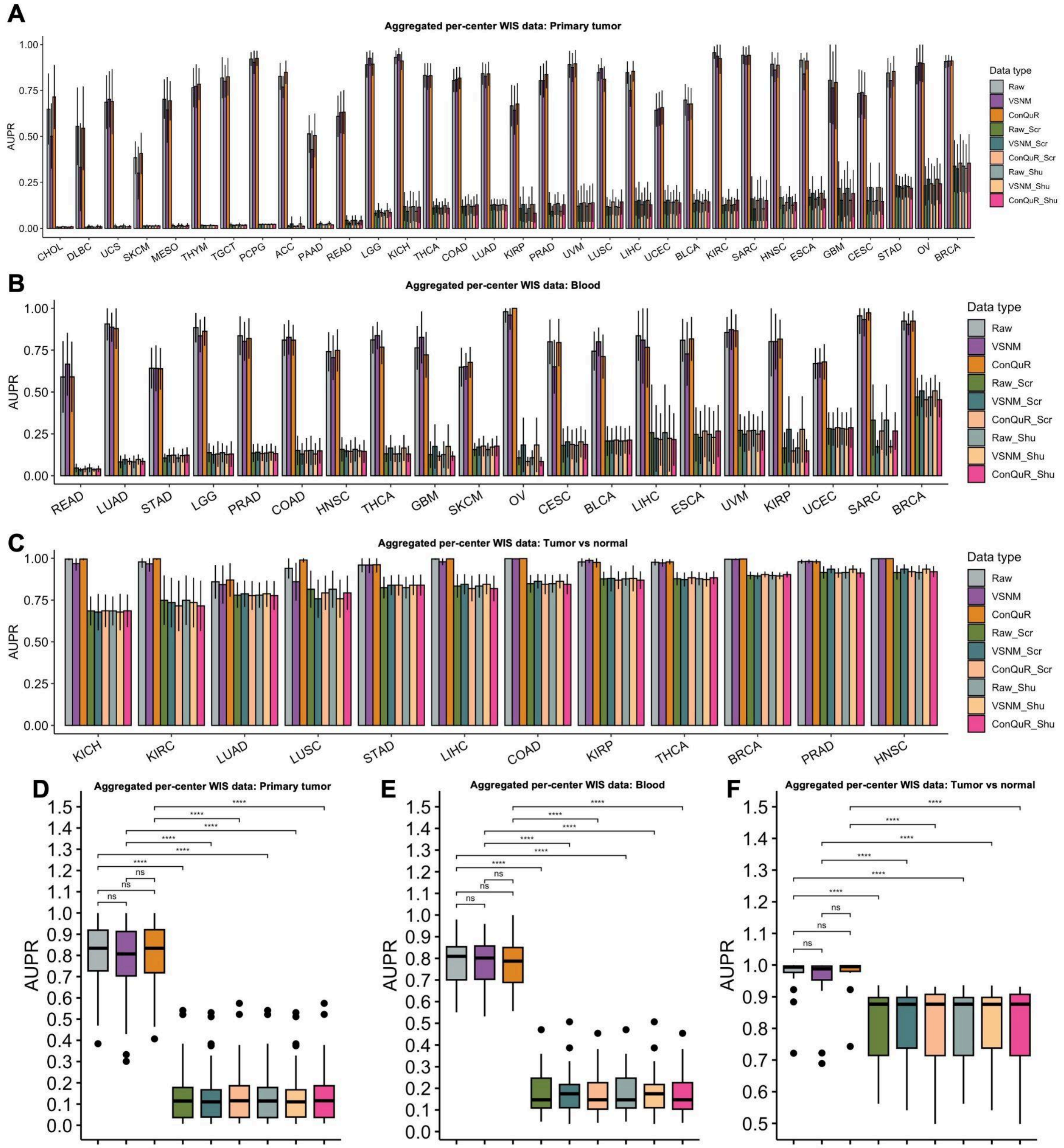

Supplementary Figure 8. Negative control analyses comparing raw, VSNM, and ConQuR WIS-overlapping data does not reveal a systematic bias from batch correction among AUPRs across all cancer types and comparisons. **(A-C)** Aggregated AUPR data across all per-batch **(A)** primary tumor, **(B)** blood sample, and **(C)** tumor versus normal comparisons using the raw, VSNM, and ConQuR WIS-overlapping data for each cancer type. Identical ML models

were run while scrambling metadata labels or shuffling feature counts for each data type. Error bars denote 99% confidence intervals. **(D-F)** Per-cancer and per-batch AUPRs were averaged across their cross-validation folds and compared among **(D)** primary tumor, **(E)** blood samples, and **(F)** tumor versus normal to calculate two-sided Wilcoxon tests with Benjamini-Hochberg correction.

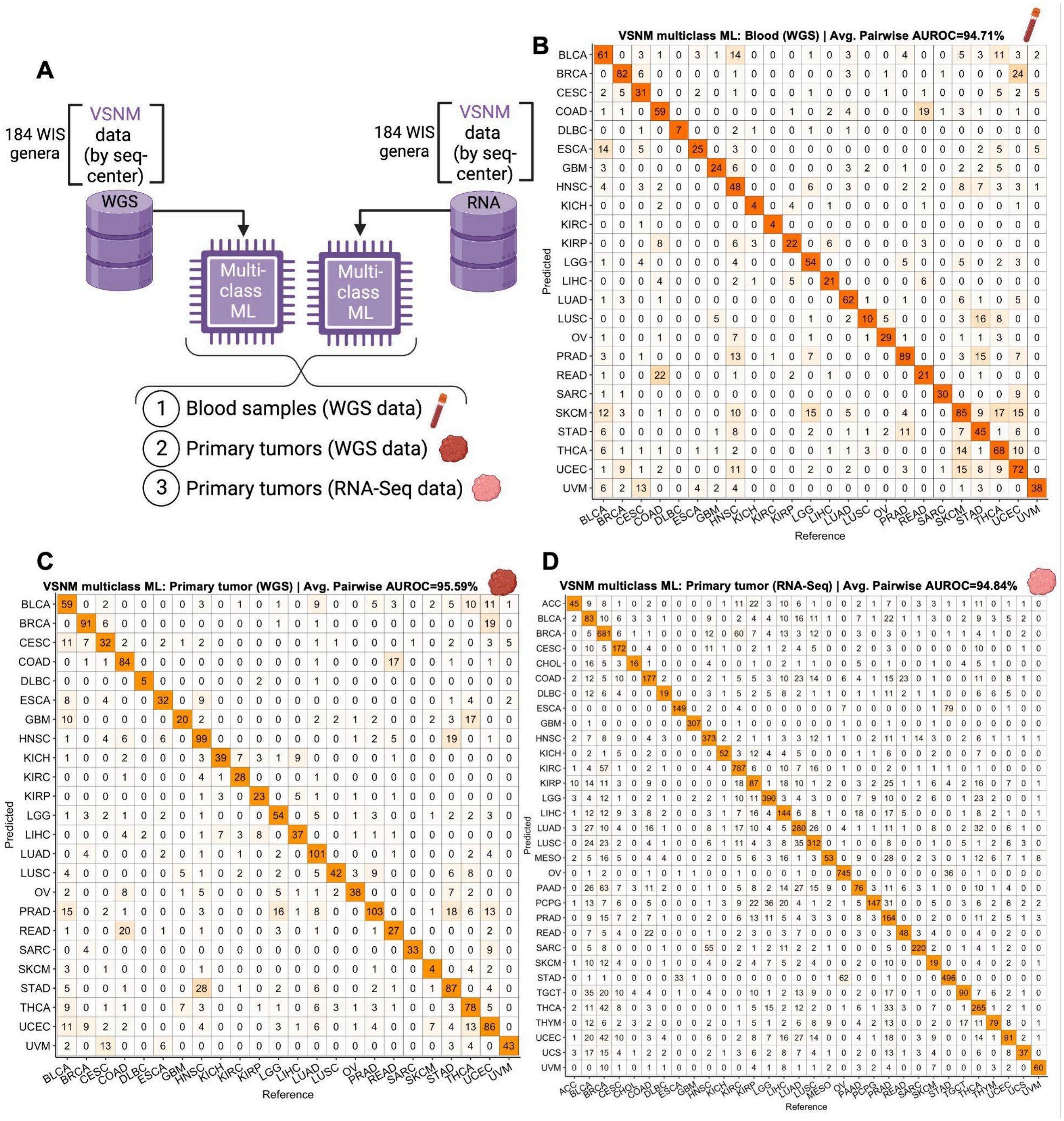

Supplementary Figure 9. VSNM-corrected, WIS-overlapping genera abundances demonstrate pan-cancer discrimination among blood and primary tumor samples across dozens of cancer types. (A) Diagram of data sources for the multiclass ML. To compare VSNM to ConQuR, which was limited to correcting one batch variable, sequencing center bias was corrected within WGS and RNA-Seq groups after subsetting analyses to Illumina HiSeq-processed samples. Multiclass ML was run independently on the VSNM-corrected WGS and RNA-Seq groups; of note, blood samples were only in the WGS group. **(B)** Multiclass gradient boosting ML across 24 cancer types using all blood samples in TCGA. Average pairwise AUROC is denoted above the confusion matrix. No information rate: 9.1%, mean balanced accuracy: 79.9%. **(C)** Multiclass gradient boosting ML across 24 cancer types using all

WGS primary tumors in TCGA. Average pairwise AUROC is denoted above the confusion matrix. No information rate: 8.5%, mean balanced accuracy: 80.9% **(D)** Multiclass gradient boosting ML across 32 cancer types using all RNA-Seq primary tumors in TCGA. Average pairwise AUROC is denoted above the confusion matrix. No information rate: 10.7%, mean balanced accuracy: 79.6%. (**B-D**) P-values are all less than 2.2×10^{-16} for comparing the no information rate to the observed accuracy. See Supplementary Fig. 1C for list of TCGA cancer type abbreviations.

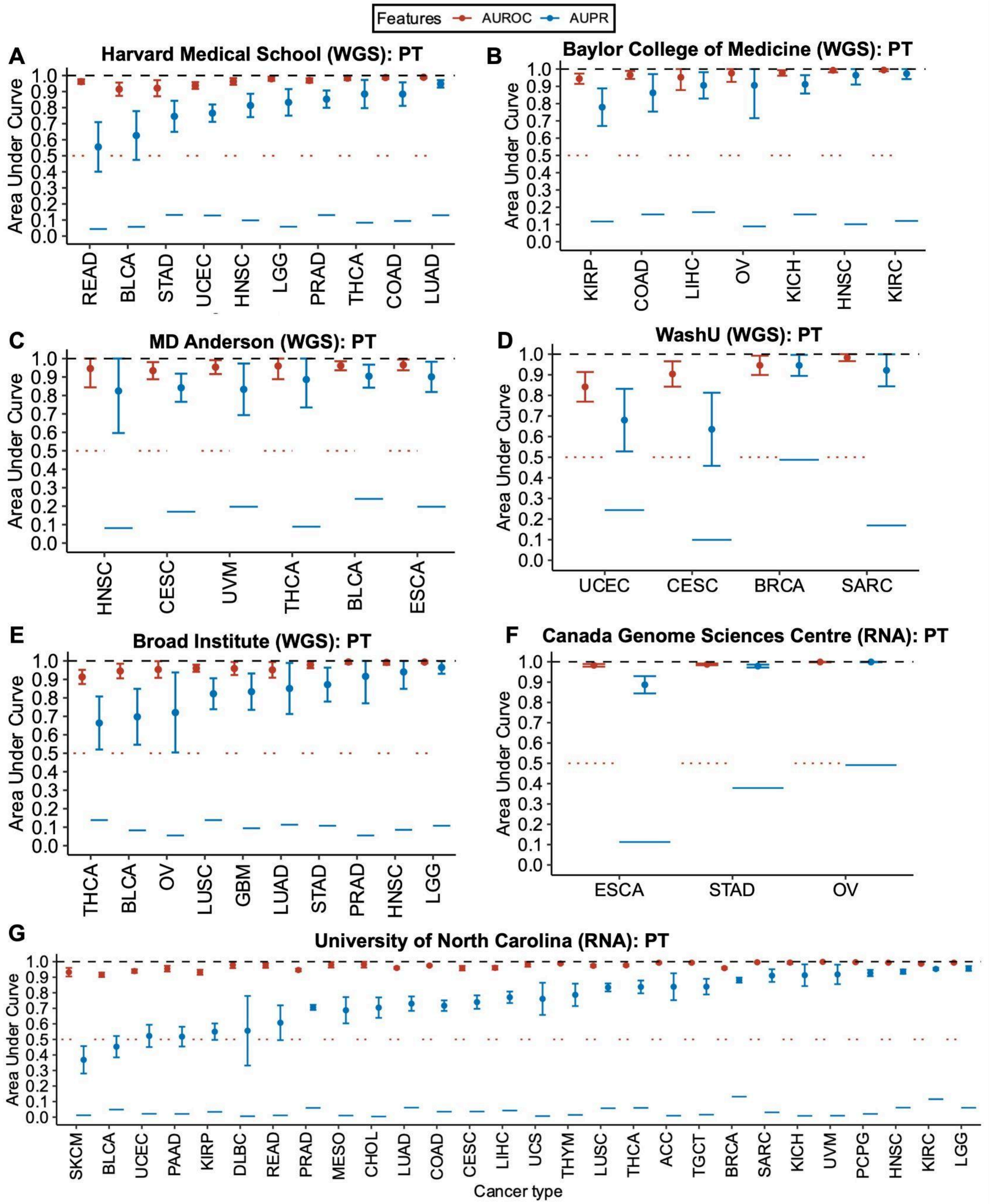

Supplementary Figure 10. Classifier performance using subset raw data with WIS-overlapping bacterial genera for cancer discrimination using primary tumor-derived microbial nucleic acids. Raw count data using WIS-overlapping bacterial genera were subset to a single sequencing center, experimental strategy (WGS or RNA-Seq), and sequencing platform (Illumina HiSeq) prior to evaluating ten-fold cross-validation gradient boosting machine

learning models. Notably, 6 of 7 sequencing centers only used one type of experimental strategy (WGS or RNA-Seq); the exception was the Broad Institute, which performed RNA-Seq on glioblastoma tumors, but since it was the only cancer type to have RNA from Broad, it could not be compared in one-cancer-type-versus-all-other predictions. Predictions were made on each of the ten holdout folds to generate average and 95% confidence intervals of discriminatory performance, as measured by AUROC and AUPR. A minimum of 20 samples were required in any comparison to be tested. **(A)** One-cancer-type-versus-all-others predictions among WGS primary tumor samples from Harvard Medical School. **(B)** One-cancer-type-versus-all-others predictions among WGS primary tumor samples from Baylor College of Medicine. **(C)** One-cancer-type-versus-all-others predictions among WGS primary tumor samples from MD Anderson. **(D)** One-cancer-type-versus-all-others predictions among WGS primary tumor samples from Washington University. **(E)** One-cancer-type-versus-all-others predictions among WGS primary tumor samples from the Broad Institute. **(F)** One-cancer-type-versus-all-others predictions among RNA-Seq primary tumor samples from Canada's Michael Smith Genome Sciences Centre. **(G)** One-cancer-type-versus-all-others predictions among RNA-Seq primary tumor samples from the University of North Carolina. **(A-G)** AUROC and AUPR measured on independent holdout folds (ten-fold cross-validation (CV)) to estimate averages (dots) and 95% confidence intervals (brackets). Red horizontal dotted lines under AUROC denote null values. Blue solid horizontal lines under AUPR denote null values, which equates the prevalence of the positive class (each cancer type) among the full set of all cancer types within a sequencing center-experimental strategy-platform subset. PT, primary tumor.

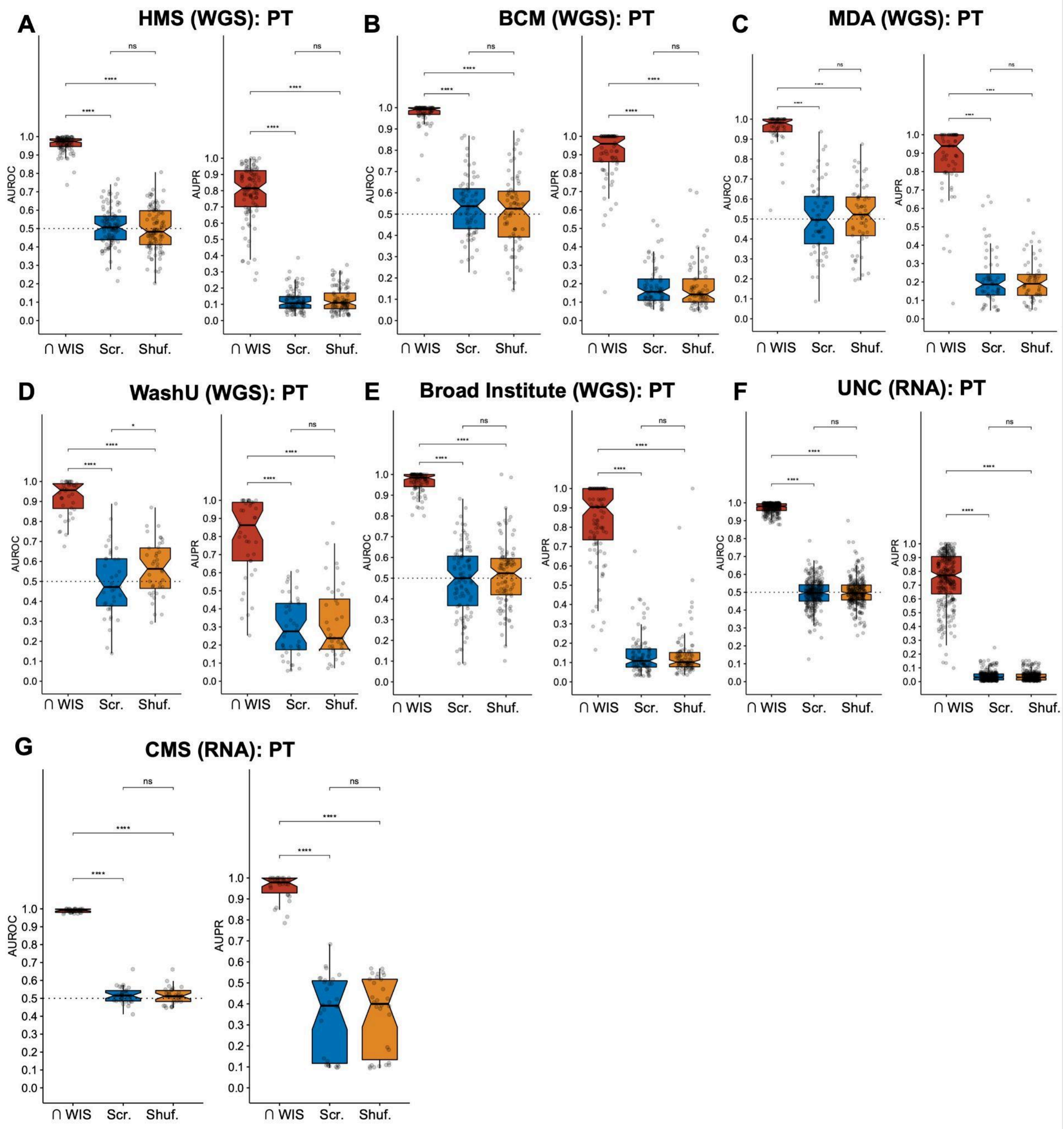

Supplementary Figure 11. WIS-overlapping raw data control analyses to verify TCGA primary tumor classifier performances. Raw count data using WIS-overlapping bacterial genera were subset to a single sequencing center, experimental strategy (WGS or RNA-Seq), and sequencing platform (Illumina HiSeq) prior to evaluating ten-fold cross-validation gradient boosting machine learning models. Performances on these raw data subsets were then compared to equivalent ML models built with scrambled metadata or shuffled samples. **(A)** Comparison of primary tumor-based ML models built on raw count data versus equivalent ones built using scrambled metadata labels or shuffled count data using WGS samples from Harvard Medical School (HMS). **(B)** Comparison of primary tumor-based ML models built on raw count

data versus equivalent ones built using scrambled metadata labels or shuffled count data using WGS samples from Baylor College of Medicine (BCM). **(C)** Comparison of primary tumor-based ML models built on raw count data versus equivalent ones built using scrambled metadata labels or shuffled count data using WGS samples from MD Anderson (MDA). **(D)** Comparison of primary tumor-based ML models built on raw count data versus equivalent ones built using scrambled metadata labels or shuffled count data using WGS samples from Washington University (WashU). **(E)** Comparison of primary tumor-based ML models built on raw count data versus equivalent ones built using scrambled metadata labels or shuffled count data using WGS samples from the Broad Institute. **(F)** Comparison of primary tumor-based ML models built on raw count data versus equivalent ones built using scrambled metadata labels or shuffled count data using RNA-Seq samples from the University of North Carolina (UNC). **(G)** Comparison of primary tumor-based ML models built on raw count data versus equivalent ones built using scrambled metadata labels or shuffled count data using RNA-Seq samples from Canada's Michael Smith Genome Sciences Centre (CMS). **(A-G)**, Pairwise two-sided Wilcox tests using performances from all ML folds, corrected for multiple hypothesis testing using the Benjamini-Hochberg method, are shown. ns: not significant (q>0.05); *: q≤0.05; **: q≤0.01; ***: q≤0.001; ****: q≤0.0001. PT, primary tumor.

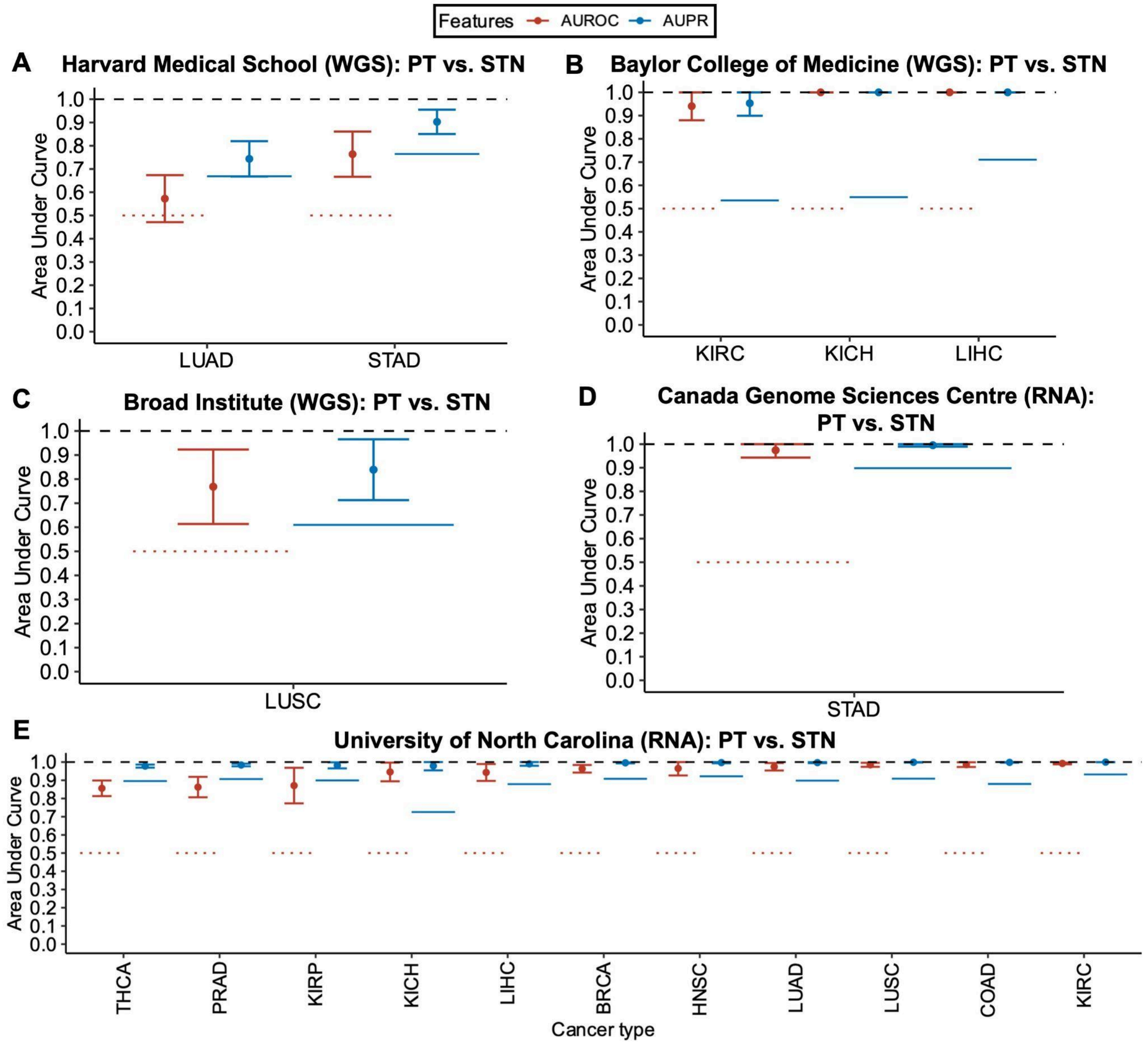

Supplementary Figure 12. Classifier performance using subset raw data with WIS-overlapping bacterial genera for primary tumor versus adjacent tissue normal discrimination. Raw count data using WIS-overlapping bacterial genera were subset to a single sequencing center, experimental strategy (WGS or RNA-Seq), and sequencing platform (Illumina HiSeq) prior to evaluating ten-fold cross-validation gradient boosting machine learning models. Predictions were made on each of the ten holdout folds to generate average and 95% confidence intervals of discriminatory performance, as measured by AUROC and AUPR. A minimum of 20 samples were required in any comparison to be tested, and neither MD Anderson nor Washington University had sufficient tumor versus normal samples in this setting to evaluate. **(A)** Primary tumor vs. adjacent normal using WGS tissue samples from Harvard Medical School. **(B)** Primary tumor vs. adjacent normal using WGS tissue samples from Baylor College of Medicine. **(C)** Primary tumor vs. adjacent normal using WGS tissue samples from the Broad Institute. **(D)** Primary tumor vs. adjacent normal using RNA-Seq tissue samples from Canada's Michael Smith Genome Sciences Centre. **(E)** Primary tumor vs. adjacent normal using RNA-Seq tissue samples from the University of North Carolina. **(A-E)** AUROC and AUPR

measured on independent holdout folds (ten-fold cross-validation (CV)) to estimate averages (dots) and 95% confidence intervals (brackets). Red horizontal dotted lines under AUROC denote null values. Blue solid horizontal lines under AUPR denote null values, which equates the prevalence of the positive class (each cancer type) among the full set of all cancer types within a sequencing center-experimental strategy-platform subset. PT, primary tumor; STN, solid tissue normal.

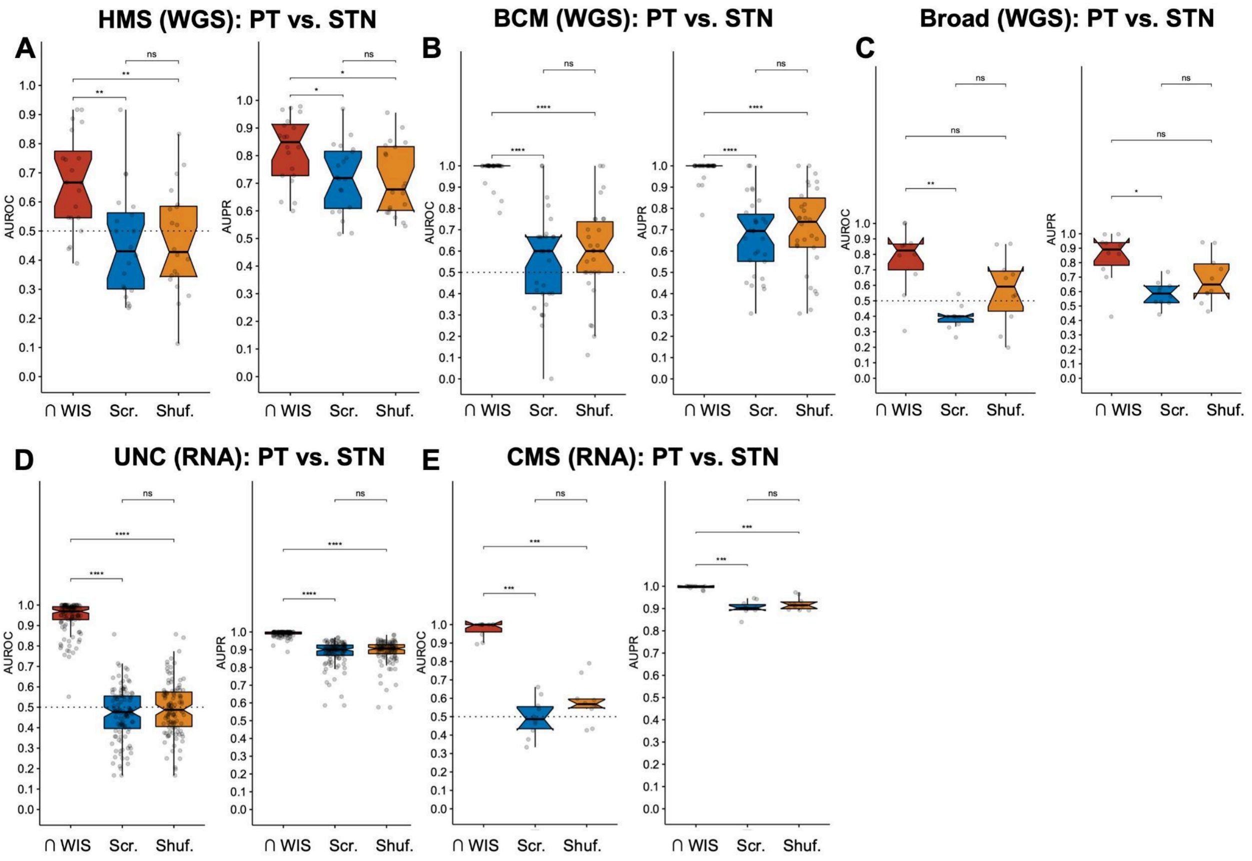

Supplementary Figure 13. WIS-overlapping raw data control analyses to verify TCGA primary tumor vs. adjacent normal classifier performances. Raw count data using WIS-overlapping bacterial genera were subset to a single sequencing center, experimental strategy (WGS or RNA-Seq), and sequencing platform (Illumina HiSeq) prior to evaluating ten-fold cross-validation gradient boosting machine learning models. Tumor vs. adjacent normal performances on these raw data subsets were then compared to equivalent ML models built with scrambled metadata or shuffled samples. **(A)** Comparison of primary tumor vs. adjacent normal ML models built on raw count data versus equivalent models built using scrambled metadata labels or shuffled count data using WGS samples from Harvard Medical School (HMS). **(B)** Comparison of primary tumor vs. adjacent normal ML models built on raw count data versus equivalent models built using scrambled metadata labels or shuffled count data using WGS samples from Baylor College of Medicine (BCM). **(C)** Comparison of primary tumor vs. adjacent normal ML models built on raw count data versus equivalent models built using scrambled metadata labels or shuffled count data using WGS samples from the Broad Institute (Broad). **(D)** Comparison of primary tumor vs. adjacent normal ML models built on raw count data versus equivalent models built using scrambled metadata labels or shuffled count data using RNA-Seq samples from the University of North Carolina (UNC). **(E)** Comparison of primary tumor vs. adjacent normal ML models built on raw count data versus equivalent models built using scrambled metadata labels or shuffled count data using RNA-Seq samples from

Canada's Michael Smith Genome Sciences Centre (CMS). **(A-E)** Pairwise two-sided Wilcox tests using performances from all ML folds, corrected for multiple hypothesis testing using the Benjamini-Hochberg method, are shown. ns: not significant ($q>0.05$); *: $q \leq 0.05$; **: $q \leq 0.01$; ***: $q \leq 0.001$; ****: $q \leq 0.0001$. PT, primary tumor; STN, solid tissue normal.

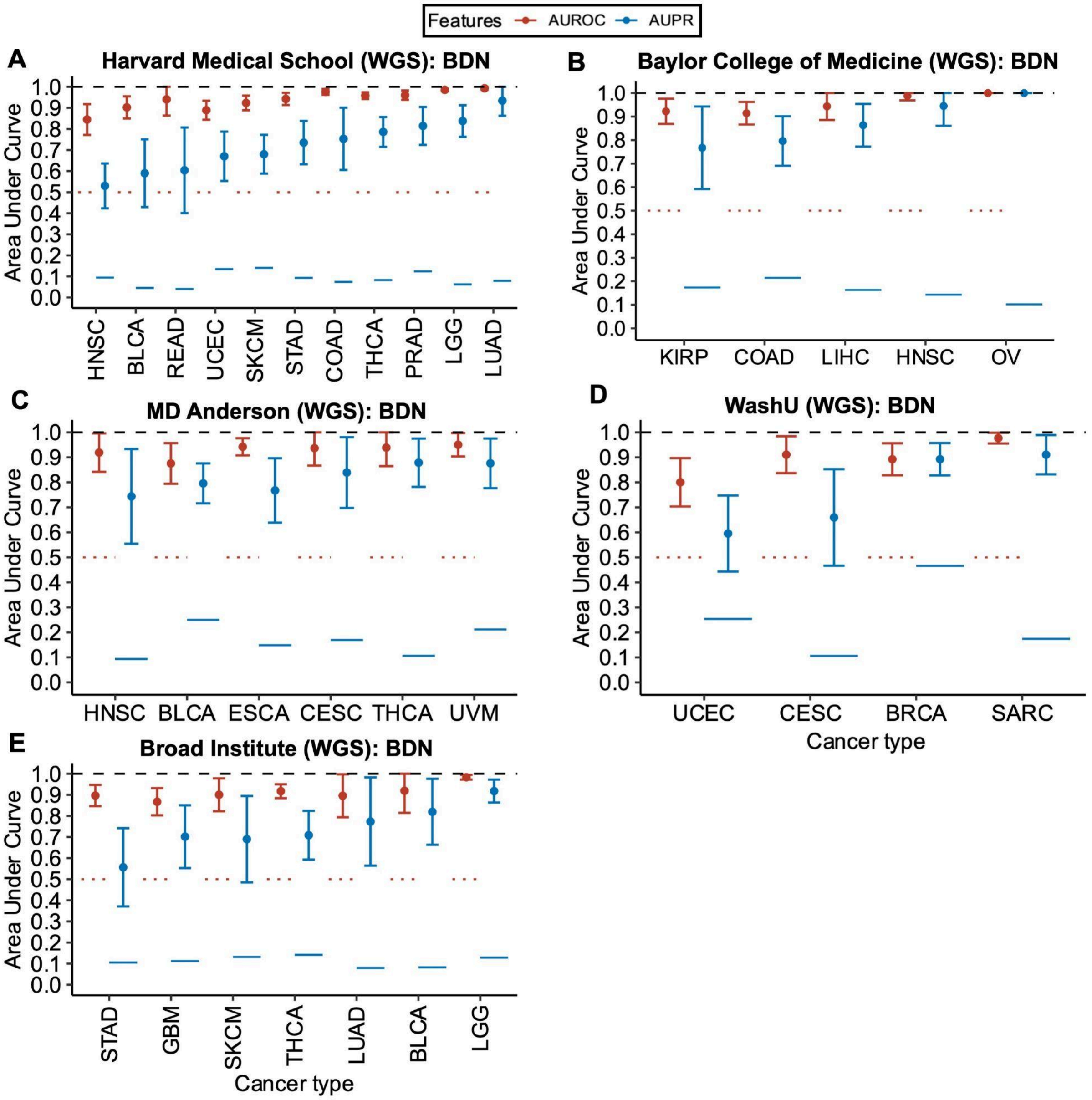

Supplementary Figure 14. Classifier performance using subset raw data with WIS-overlapping bacterial genera for cancer discrimination using blood-derived microbial nucleic acids. Raw count data using WIS-overlapping bacterial genera were subset to a single sequencing center, experimental strategy (WGS or RNA-Seq), and sequencing platform (Illumina HiSeq) prior to evaluating ten-fold cross-validation gradient boosting machine learning models. All blood derived normal (BDN) samples were WGS. Predictions were made on each of the ten holdout folds to generate average and 95% confidence intervals of discriminatory performance, as measured by AUROC and AUPR. A minimum of 20 samples were required in any comparison to be tested. **(A)** One-cancer-type-versus-all-others predictions among WGS blood samples from Harvard Medical School. **(B)** One-cancer-type-versus-all-others predictions among WGS blood samples from Baylor College

of Medicine. **(C)** One-cancer-type-versus-all-others predictions among WGS blood samples from MD Anderson. **(D)** One-cancer-type-versus-all-others predictions among WGS blood samples from Washington University (WashU). **(E)** One-cancer-type-versus-all-others predictions among WGS blood samples from the Broad Institute. **(A-E)** AUROC and AUPR measured on independent holdout folds (ten-fold cross-validation (CV)) to estimate averages (dots) and 95% confidence intervals (brackets). Red horizontal dotted lines under AUROC denote null values. Blue solid horizontal lines under AUPR denote null values, which equates the prevalence of the positive class (each cancer type) among the full set of all cancer types within a sequencing center-experimental strategy-platform subset. BDN, blood derived normal.

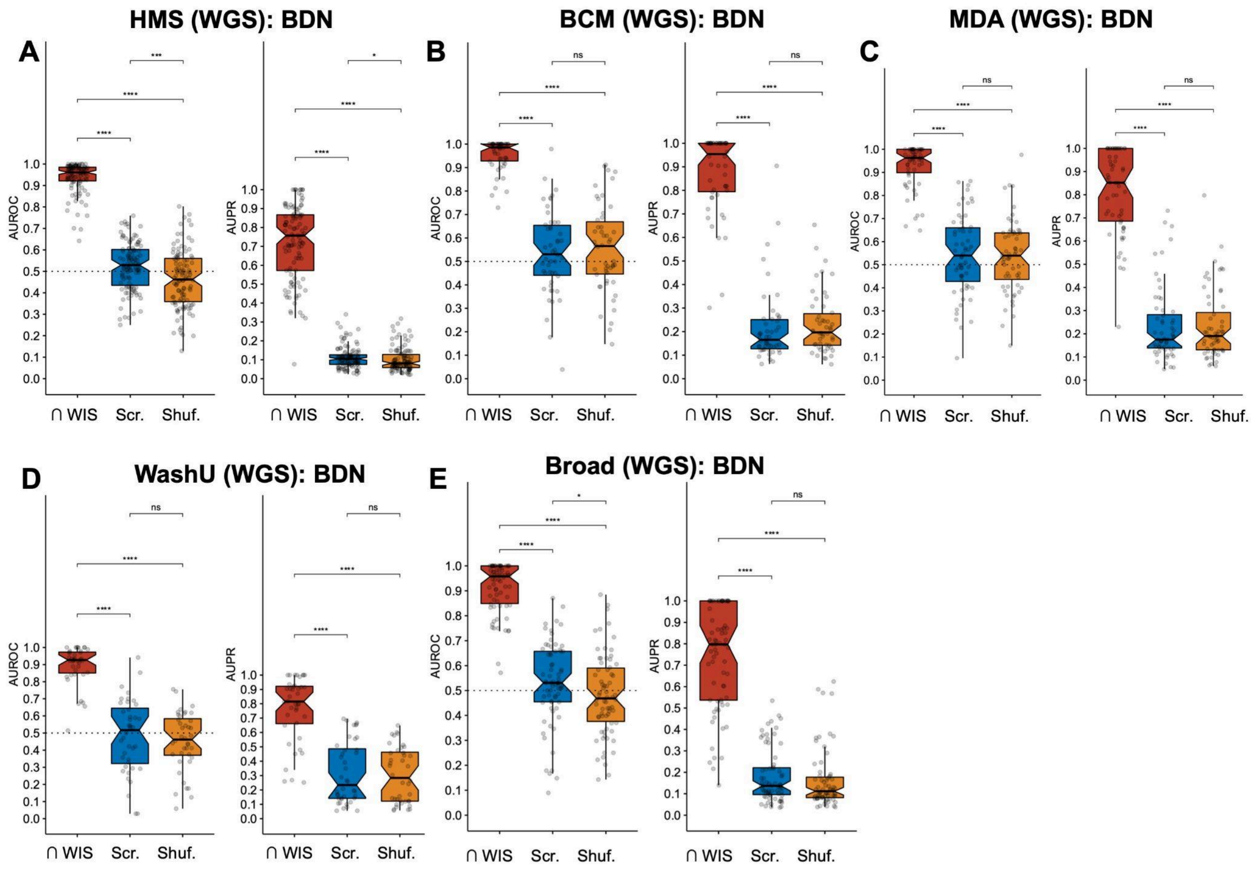

Supplementary Figure 15. WIS-overlapping raw data control analyses to verify TCGA blood classifier performances. Raw count data using WIS-overlapping bacterial genera were subset to a single sequencing center, experimental strategy (all blood samples were WGS), and sequencing platform (Illumina HiSeq) prior to evaluating ten-fold cross-validation gradient boosting machine learning models. Blood-related performances on these raw data subsets were then compared to equivalent ML models built with scrambled metadata or shuffled samples. **(A)** Comparison of blood-based ML models built on raw count data versus equivalent models built using scrambled metadata labels or shuffled count data using WGS samples from Harvard Medical School (HMS). **(B)** Comparison of blood-based ML models built on raw count data versus equivalent models built using scrambled metadata labels or shuffled count data using WGS samples from Baylor College of Medicine (BCM). **(C)** Comparison of blood-based ML models built on raw count data versus equivalent models built using scrambled metadata labels or shuffled count data using WGS samples from MD Anderson (MDA). **(D)** Comparison of blood-based ML models built on raw count data versus equivalent models built using scrambled metadata labels or shuffled count data using WGS samples from Washington University (WashU). **(E)** Comparison of blood-based ML models built on raw count data versus equivalent models built using scrambled metadata labels or shuffled count data using WGS samples from the Broad Institute. **(A-E)** Pairwise two-sided Wilcox tests using performances from all ML folds, corrected for multiple hypothesis testing using the Benjamini-Hochberg method, are shown. ns:

not significant (q>0.05); *: q≤0.05; **: q≤0.01; ***: q≤0.001; ****: q≤0.0001. BDN, blood derived normal.

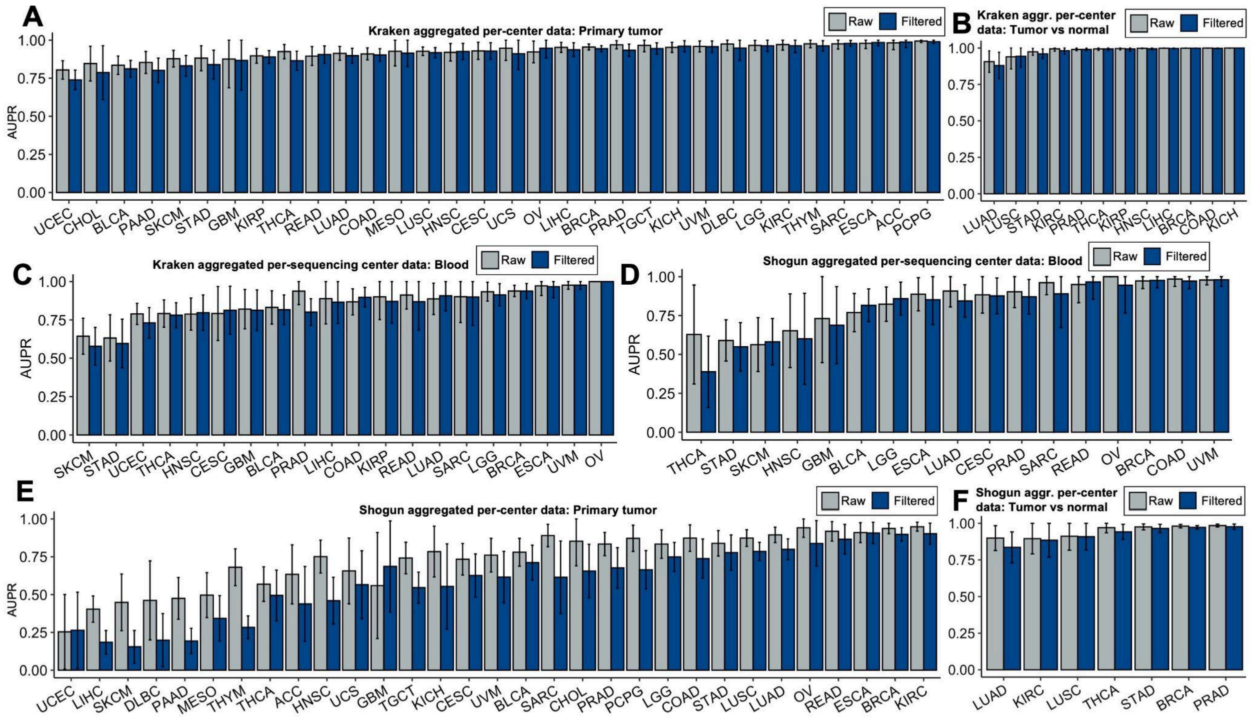

Supplementary Figure 16. Removing genera with any human sequence contamination in the original databases does not impact downstream conclusions. **(A-C)** Aggregated AUPR data across all per-batch **(A)** primary tumor and **(B)** tumor versus normal, and **(C)** blood sample comparisons using the original Kraken raw and filtered data. **(D-F)** Aggregated AUPR data across all per-batch **(D)** blood sample **(E)** primary tumor, and **(F)** tumor versus normal comparisons using the SHOGUN/WoLr1 raw and filtered data. **(A-F)** Error bars denote 99% confidence intervals.

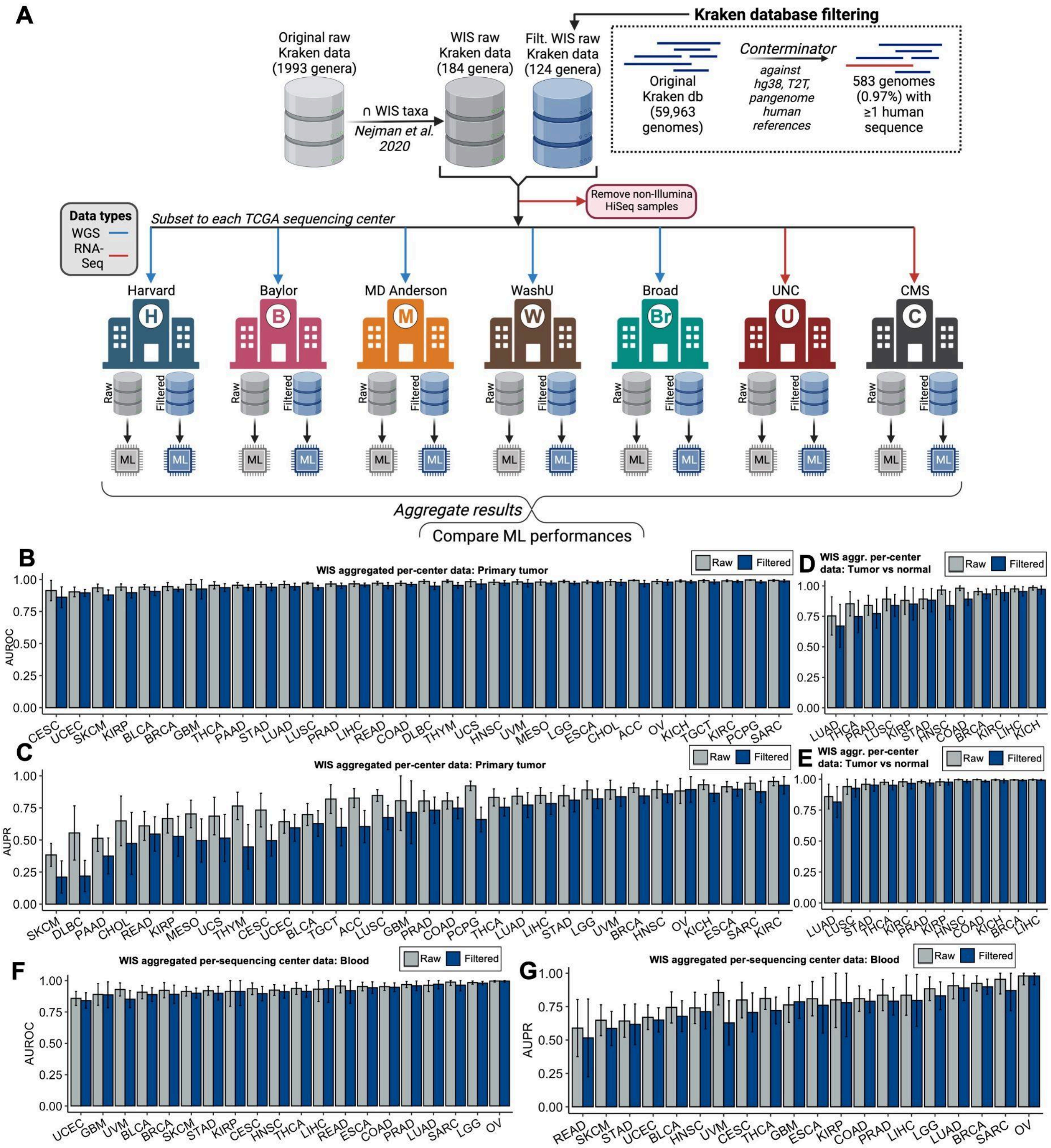

Supplementary Figure 17. Conservative removal of WIS-overlapping genera having any genome(s) with human sequences maintains cancer type-specific conclusions. (A) Microbial genomes identified by Conterminator to have any regions shared with hg38, T2T-CHM13, and human pangenome references were intersected with WIS-overlapping genera, and all implicated genera were conservatively discarded. A data splitting strategy was applied to compare filtered versus raw versions of the WIS-overlapping data. **(B-C)** Aggregated **(B)** AUROC and **(C)** AUPR data across all per-batch primary tumor comparisons. **(D-E)** Aggregated **(D)** AUROC and **(E)** AUPR data across all per-batch tumor versus normal

comparisons. **(F-G)** Aggregated **(G)** AUROC and **(G)** AUPR data across all per-batch blood sample comparisons.

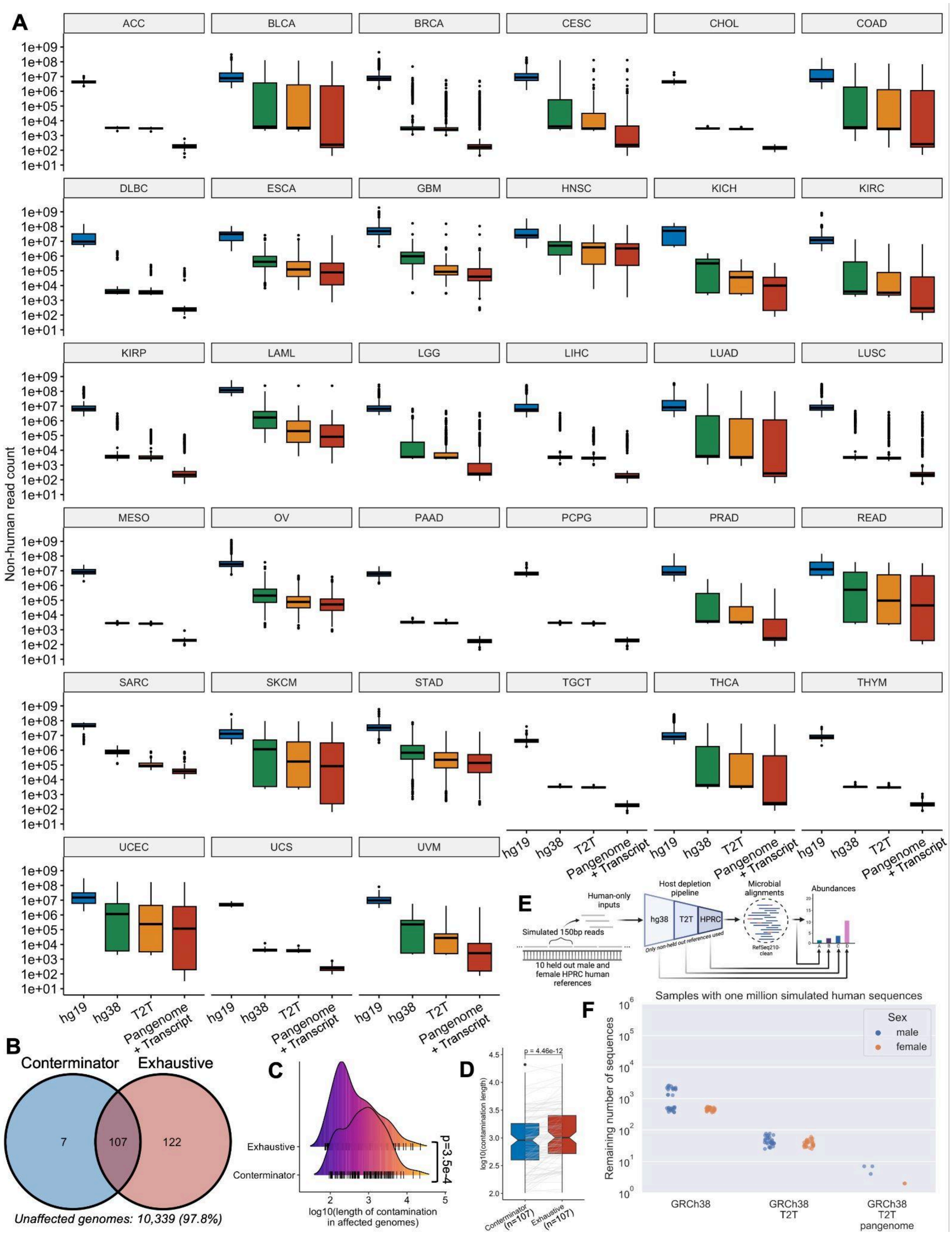

Supplementary Figure 18. Non-human read decreases in TCGA cancer types with successive host depletion and evaluation of Exhaustive versus Conterminator on WoLr1.

(A) Per cancer type decreases in non-human read counts after successive host depletion with hg19 (blue), hg38 (green), T2T-CHM13 (orange), and HPRC plus GENCODE transcripts (red). GENCODE transcripts were only used for host filtering with RNA-seq data. **(B)** Number of WoLr1 genomes found to have at least one region of human sequence overlap detected by Conterminator and/or Exhaustive. **(C)** Unpaired, cumulative length of human sequence contamination in WoLr1 genomes found to have at least one region of human sequence overlap via Conterminator or Exhaustive. Wilcoxon test inset. **(D)** Paired cumulative length of human sequence contamination in 107 WoLr1 genomes found to have at least one region of human sequence overlap via Conterminator and Exhaustive. Wilcoxon rank-sum test inset. **(E)** Simulation strategy for estimating false positive rate of human-only reads surviving successive host depletion. Hold out male and female HPRC genomes were used only for simulated Illumina read generation and not used for subsequent host filtering. Surviving reads were enumerated after each step. **(F)** Number of surviving human-only reads after each level of host depletion: hg38 alone, hg38 with T2T-CHM13, or hg38 with T2T-CHM13 and HPRC. Colored by biological sex of originally held HPRC genomes.

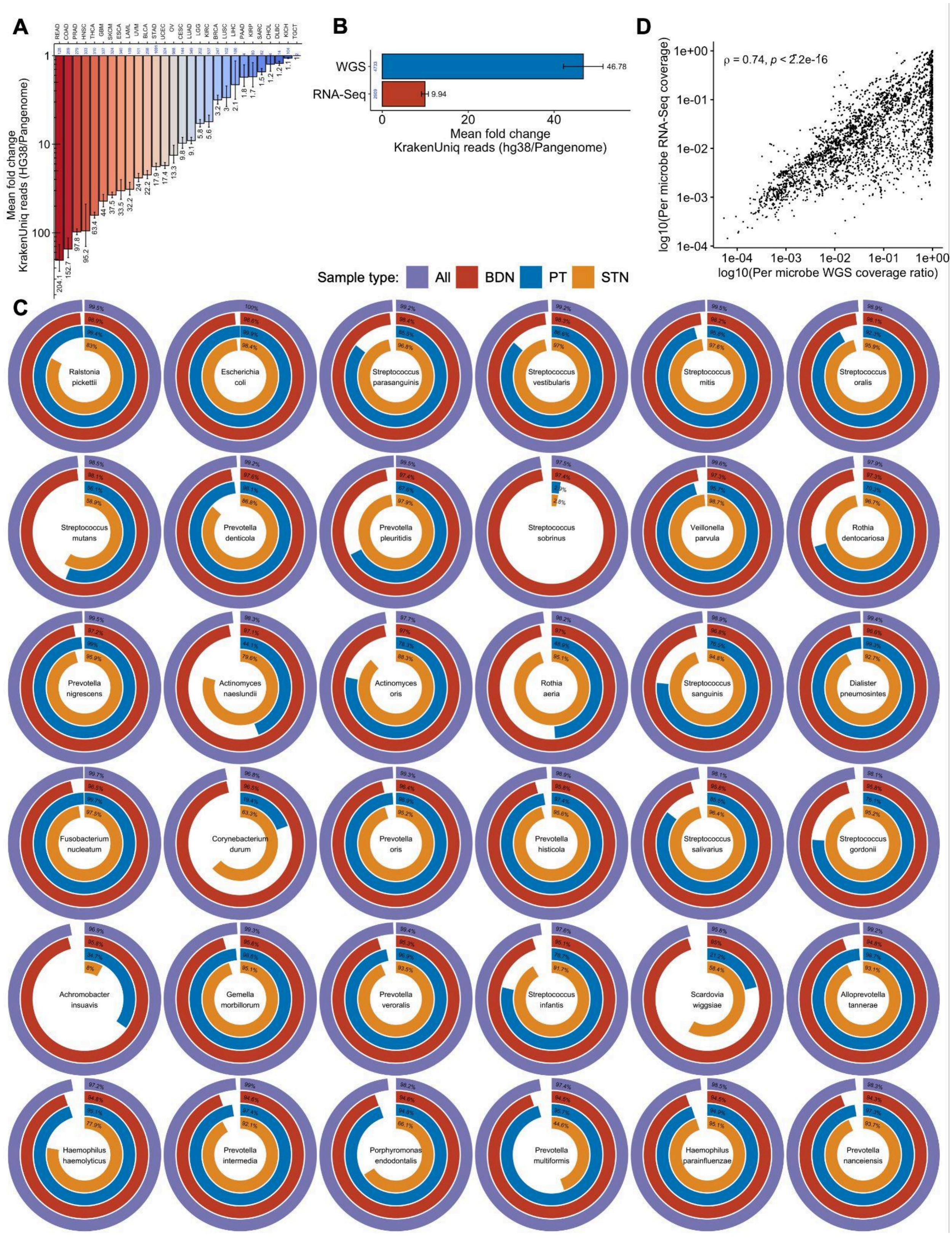

Supplementary Figure 19. Impact of pangenome host depletion on KrakenUniq-MicrobialDB microbial read counts and high aggregate genome coverages of

RS210-clean mapped species. **(A)** Per-sample, per-cancer type mean fold changes (MFCs) in KrakenUniq-MicrobialDB counts between hg38- and HPRC-transcript-depleted data. Per-cancer average MFCs inset below bars. Sample counts inset in blue above bars. Overlaid error bars denote standard errors. Zero-valued samples excluded to avoid ratios of infinity. **(B)** Per-sample, per-experimental strategy MFCs in KrakenUniq-MicrobialDB counts between hg38- and HPRC-transcript-depleted data. Per-experimental strategy average MFCs inset to right of bars. Sample counts inset in blue to left of bars. Overlaid error bars denote standard errors. Zero-valued samples excluded to avoid ratios of infinity. **(C)** Radial bar plots of top 36 RS210-clean-mapping species based on aggregate genome coverages in blood samples. Microbial genome coverages are colored by the samples over which they were aggregated: all (purple), blood only (red), primary tumor only (blue), solid tissue normal only (orange). **(D)** Comparison of WGS-and RNA-Seq derived aggregate microbial genome coverages in TCGA among non-viral, human-associated species found in UNITN, UHGG, WIS, or pathogenic bacteria references. Each dot denotes a genome associated with those species.

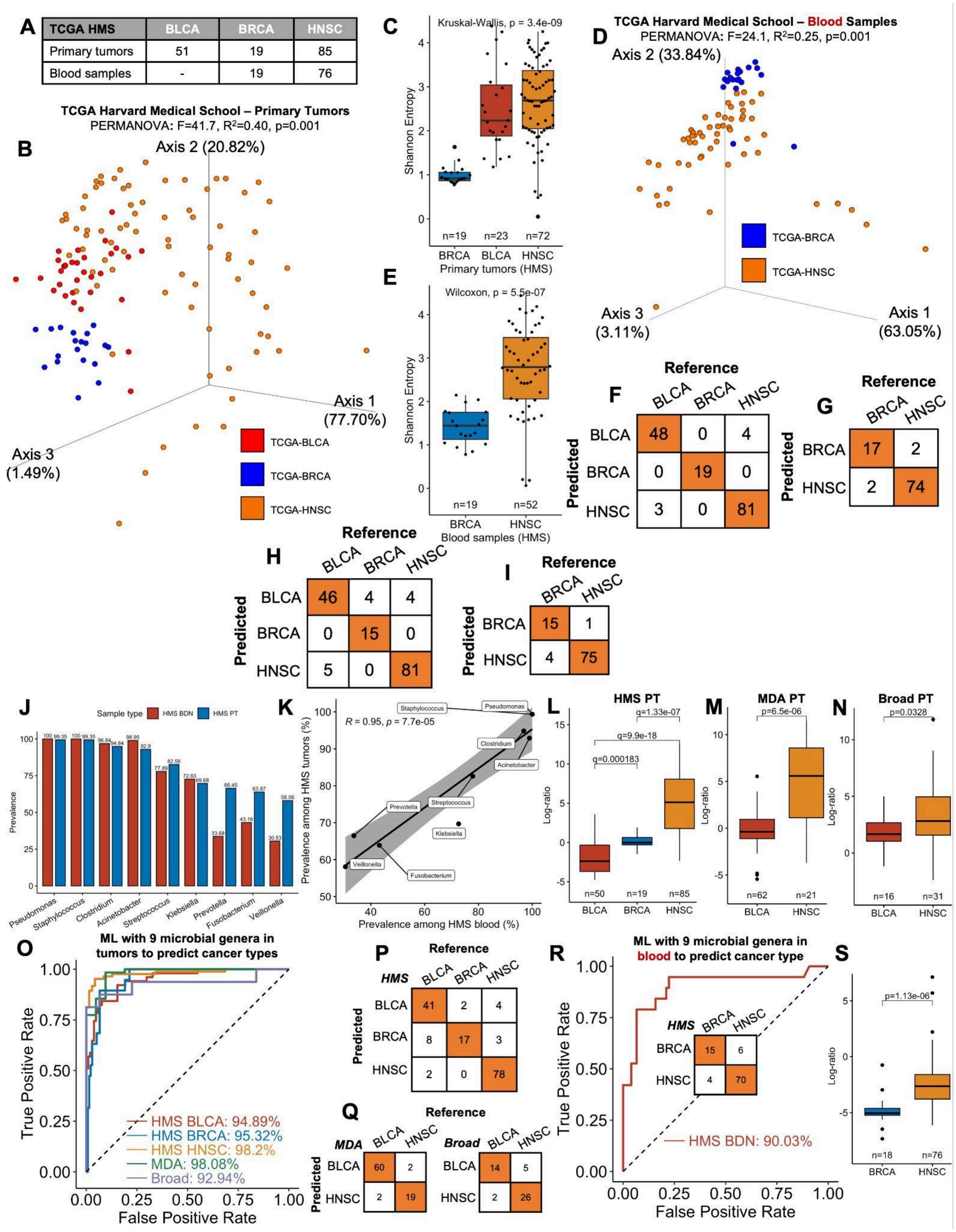

Supplementary Figure 20. Re-analysis of the data provided by Gihawi et al. [8] **reveal cancer type-specific microbiomes. (A)** The breakdown of samples derived from TCGA's

Harvard Medical School (HMS), which contributed the most samples of any sequencing center among their samples (284 of 728, 39.0%), and was the only sequencing center with >1 cancer type among both PT and BDN samples. To avoid the need for batch correction, samples were analyzed using raw supplementary data from Gihawi et al. [8] in subsets from a single sequencing center (here, HMS), sequencing platform (Illumina HiSeq), and experimental strategy (WGS). The data were not transformed in any way other than removing the *Homo* genus counts since it was included in their KrakenUniq-MicrobialDB mappings. **(B)** Aitchison-based principal coordinates analysis (RPCA) on the raw data, excluding *Homo* counts, across all primary tumors (PTs) from HMS. RPCA does not require rarefaction and none was used. PERMANOVA with 999 iterations was run to estimate effect sizes of cancer type separation, which was significant (inset values, p=0.001). Colors reflect cancer types and are uniform across all subpanels of this figure. **(C)** After RPCA, Shannon entropies were calculated on all PT samples from HMS using raw data (excluding *Homo* counts) rarefied to approximately the first quartile of sample counts. A Kruskal-Wallis test showed significant differences in Shannon diversity among cancer types ($p=3.4\times10^{-9}$). **(D)** RPCA was then applied to all blood derived normal (BDN) samples from HMS using raw data (excluding *Homo* counts). PERMANOVA with 999 iterations was run to estimate effect sizes of cancer type separation, which was significant (inset values, p=0.001). **(E)** After RPCA, Shannon entropies were calculated on all BDN samples from HMS using raw data (excluding *Homo* counts) rarefied to approximately the first quartile of sample counts. A Kruskal-Wallis test showed significant differences in Shannon diversity among cancer types ($p=5.5\times10^{-7}$). **(F)** Having found cancer type-specific differences, we evaluated if multiclass machine learning could discriminate between cancer types using the raw data (excluding *Homo* counts) from all HMS PT samples. Gradient boosting machines were applied with 10-fold cross-validation such that every sample was left out once, and their predictions were used to generate a confusion matrix. The mean balanced accuracy was 96.9% in comparison to the no information rate (NIR) of 54.84% ($p<2.2\times10^{-16}$). **(G)** 10-fold cross-validation using gradient boosting machines on HMS BDN samples. The balanced accuracy was 93.4% in comparison to the NIR of 80% ($p=9.3\times10^{-6}$). **(H)** To guard against contamination, features were subset to WIS-overlapping genera (n=149 genera) to evaluate if multiclass machine learning could discriminate between cancer types using the raw data from all HMS PT samples. Gradient boosting machines were applied with 10-fold cross-validation, and their predictions were used to generate a confusion matrix. The mean balanced accuracy was 91.6% in comparison to the no information rate (NIR) of 54.84% ($p<2.2\times10^{-16}$). **(I)** After subsetting to WIS-overlapping genera (n=149 genera), 10-fold cross-validation using gradient boosting machines was applied on HMS BDN samples. The balanced accuracy was 88.82% in comparison to the NIR of 80% ($p=4.4\times10^{-5}$). **(J)** Nine WIS-overlapping bacterial genera were selected in the Gihawi et al. [8] raw data that are commonly associated with human health and disease: *Pseudomonas, Staphylococcus, Clostridium, Acinetobacter, Streptococcus, Klebsiella, Prevotella, Fusobacterium,* and *Veillonella*. Prevalences for these 9 genera were calculated in the HMS subset of PT and BDN samples. Across all 9 genera, the average PT prevalence was 80.79%, and the average BDN prevalence was 72.63%. **(K)** The PT and BDN prevalences in panel (J) were compared, finding significant correlation (Pearson R=0.95, $p=7.7\times10^{-5}$), suggesting the relatedness of tumors and blood, despite differences in their sample processing. The absolute prevalence is higher in

tumors than blood, which is expected based on the relative reduction in microbial biomass. **(L-N)** Cancer type differences were then evaluated using log-ratios of the 9 genera raw abundances, which is a compositionally-coherent comparison [29,30]. The following 7 genera abundances were summed in the numerator per sample: *Prevotella, Fusobacterium, Streptococcus, Veillonella, Klebsiella, Clostridium, Pseudomonas*. The following 2 genera abundances were summed in the denominator per sample: *Staphylococcus, Acinetobacter*. After summating their abundances, the numerators and denominators were divided, followed by taking the log2 to obtain the per sample log-ratio. This process was done within each sequencing center subset to avoid needing batch correction, including **(L)** HMS PT samples, **(M)** MD Anderson PT samples, and **(N)** the Broad Institute PT samples. No other sequencing center subsets in the Gihawi et al. [8] data had >1 cancer type available to compare. All statistical comparisons used Wilcoxon tests with Holm multiple testing correction in panel (L). **(O)** Using the 9 genera's raw abundances in primary tumors, machine learning (ML) was run within each sequencing center subset to predict cancer type. Since HMS had 3 cancer types, multiclass ML was run, followed by calculating the performance using each class versus all others to obtain 2-class ROC curves (e.g., "HMS BLCA" denotes the performance of bladder cancer versus breast and head and neck cancers at HMS). MD Anderson (MDA) primary tumors only comprised BLCA and HNSC cancers, and the same was true for Broad Institute ("Broad") primary tumors. The AUROC was calculated for each 2-class model (inset text) and the ROC curves were overlaid. **(P)** Confusion matrix of multiclass ML among the HMS PT samples. The mean balanced accuracy was 90.82%, which was significantly better than the no information rate (NIR) ($p<2.2\times10^{-16}$). **(Q)** Left: Confusion matrix of 2-class ML among the MDA PT samples, wherein the balanced accuracy was 93.63%, which was significantly better than the no information rate (NIR) ($p=8.61\times10^{-7}$). Right: Confusion matrix of 2-class ML among the Broad Institute PT samples, wherein the balanced accuracy was 85.69%, which was significantly better than the no information rate (NIR) ($p=2.84\times10^{-3}$). **(R)** ROC curve of the 2-class ML results among HMS BDN samples, with AUC and the confusion matrix inset. Balanced accuracy was 85.53% and significantly better than the no information rate (NIR) (p=0.011). **(S)** Log-ratios using the 9 genera's raw abundances were calculated among HMS blood samples using the same numerators and denominators as in panels (C-E), with significant separation between cancer types ($p=1.13\times10^{-6}$). Wilcoxon testing was used to compare the log-ratios.

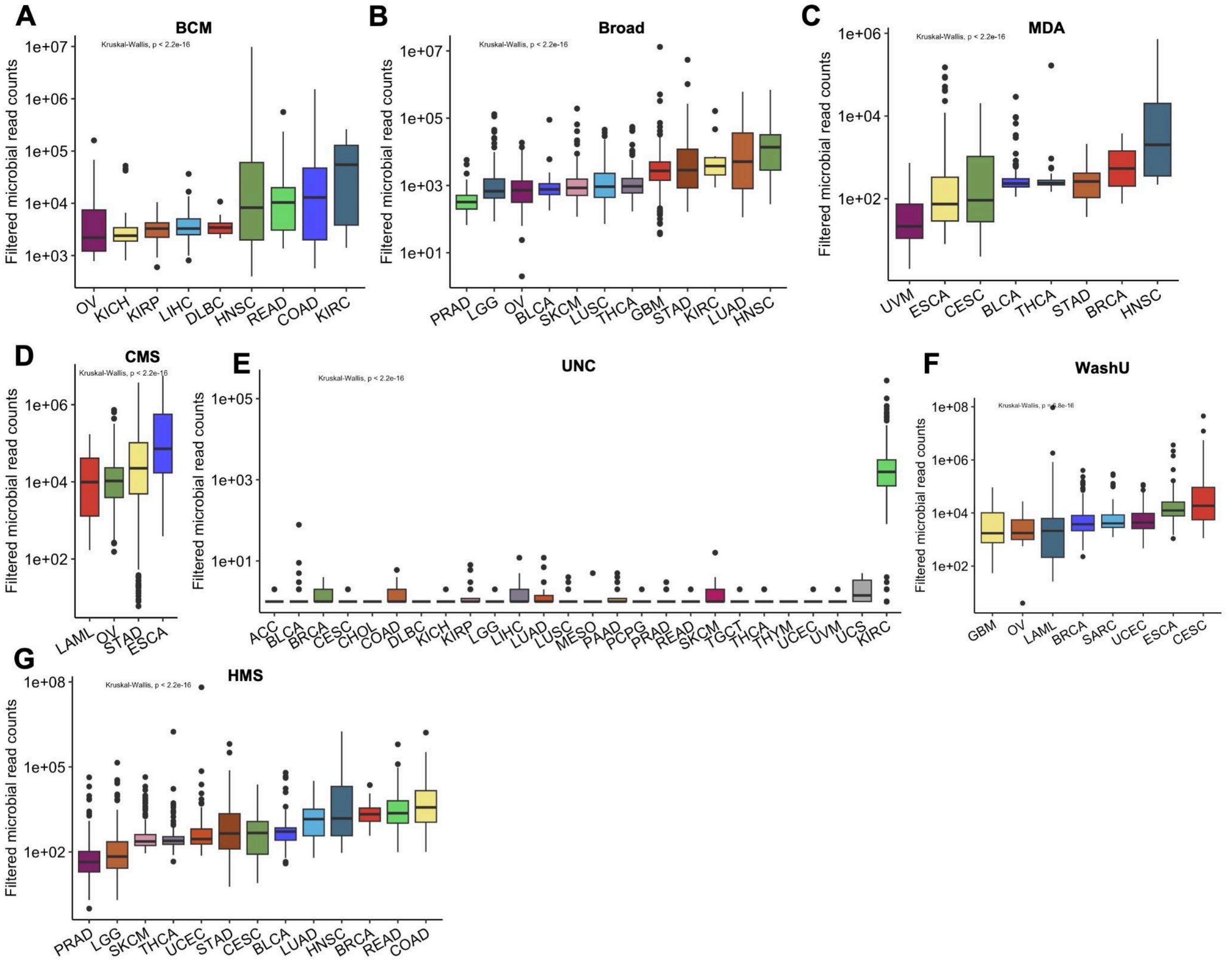

Supplementary Figure 21. Filtered microbial reads per TCGA sequencing center using the T2T-KrakenUniq-MicrobialDB pipeline. (A-G) Summated KrakenUniq-MicrobialDB microbial read counts from filtered 294 genera across **(A)** Baylor College of Medicine (WGS), **(B)** the Broad Institute (WGS), **(C)** MD Anderson (WGS), **(D)** Canada's Michael Smith Genome Sciences Centre (RNA-Seq), **(E)** the University of North Carolina (RNA-Seq), **(F)** Washington University (WGS), and **(G)** Harvard Medical School (WGS). All samples from each center included. Kruskall-Wallis test values inset.

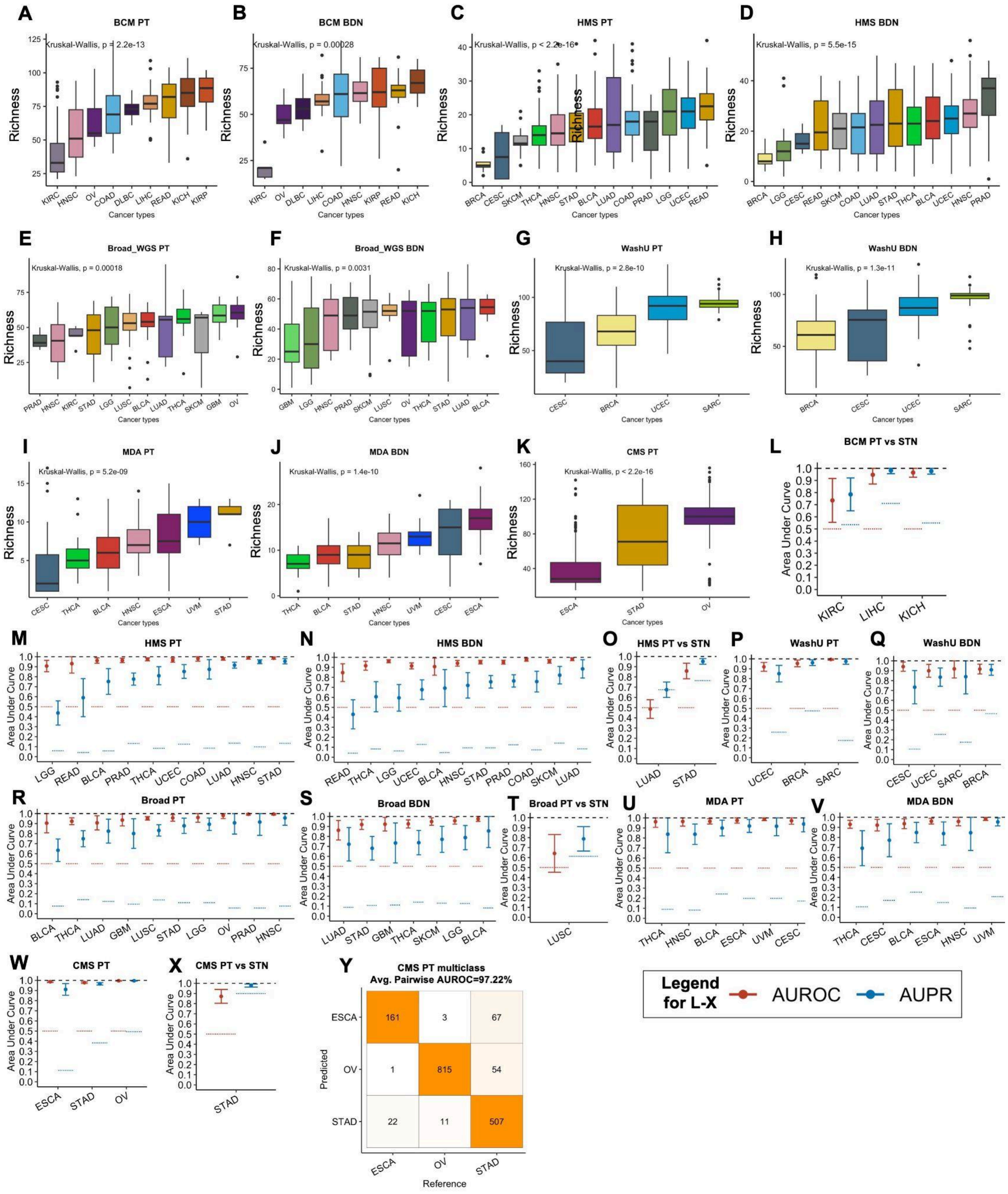

Supplementary Figure 22. Alpha diversity and machine learning (ML) reveals cancer type specific microbiomes in individual TCGA batches using KrakenUniq-MicrobialDB filtered genera. (A-K) Observed number of filtered genera after rarefaction to approximately the first quartile of read counts per sequencing center and sample type (primary tumor or blood) TCGA batch among **(A)** Baylor College of Medicine primary tumors (PTs), **(B)** Baylor College of

Medicine blood derived normals (BDNs), **(C)** Harvard Medical School PTs, **(D)** Harvard Medical School BDNs, **(E)** Broad Institute (WGS only) PTs, **(F)** Broad Institute (WGS only) BDNs, **(G)** Washington University PTs, **(H)** Washington University BDNs, **(I)** MD Anderson PTs, **(J)** MD Anderson BDNs, and **(K)** Canada's Michael Smith Genome Sciences Centre PTs. Kruskal-Wallis tests inset. **(L-X)** Per batch, one-cancer-type-versus-all-others ML using PTs or BDNs, or tumor-versus-normal (PT vs STN) ML, among **(L)** Baylor College of Medicine, **(M-O)** Harvard Medical School, **(P-Q)** Washington University, **(R-T)** Broad Institute (WGS only), **(U-V)** MD Anderson, **(W-X)** Canada's Michael Smith Genome Sciences Centre (CMS). Error bars denote averages (dots) and 95% confidence intervals (brackets) of 10-fold cross-validation. Null AUPR and AUPR shown as dotted horizontal lines. **(Y)** Multiclass ML within CMS.

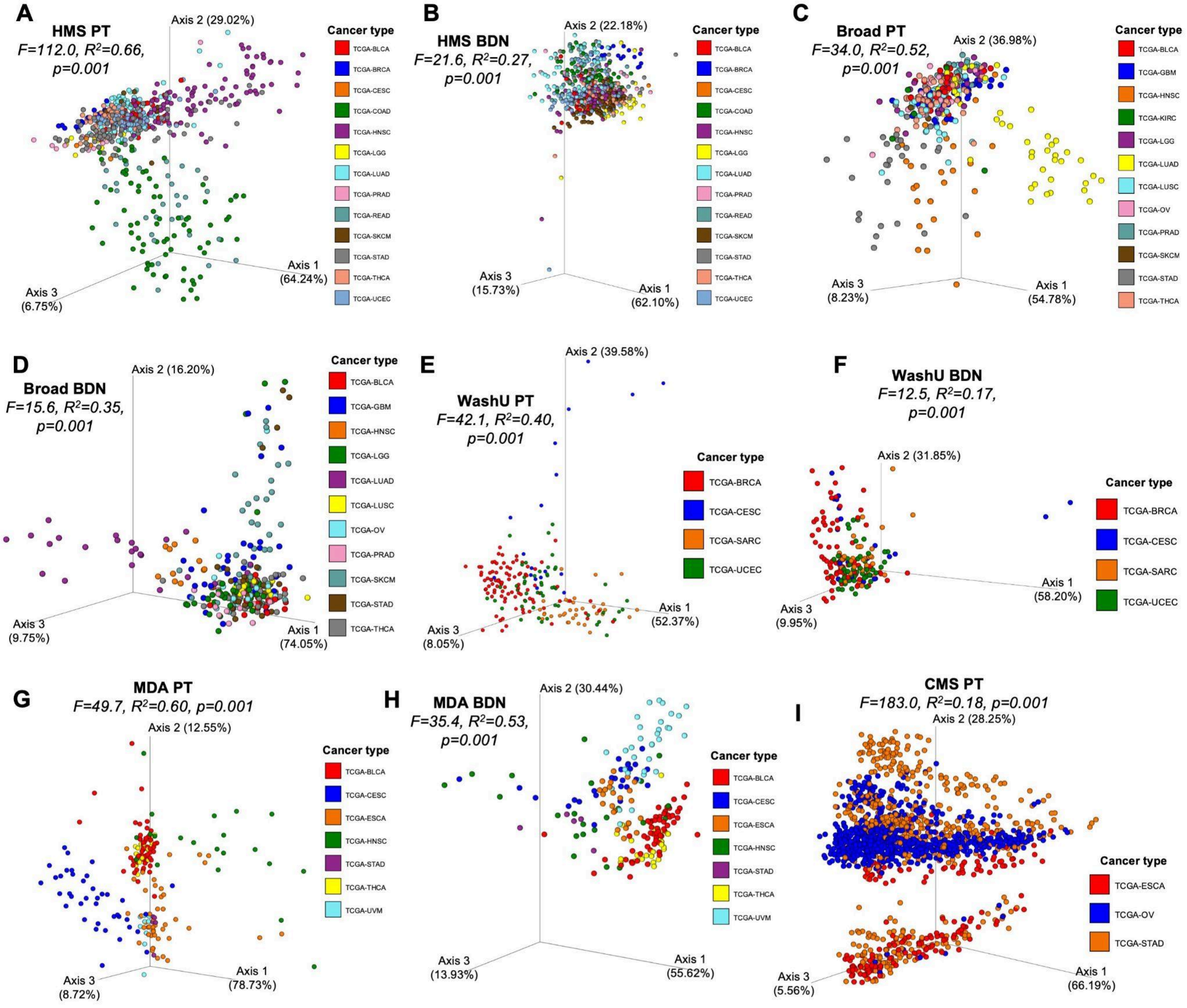

Supplementary Figure 23. Aitchison beta diversity in individual TCGA batches using KrakenUniq-MicrobialDB filtered genera reveals cancer type specific microbiomes. (A-I) Aitchison beta diversities calculated by RPCA [31] using KrakenUniq-MicrobialDB-derived 294 filtered genera among **(A)** Harvard Medical School primary tumors (PTs), **(B)** Harvard Medical School blood derived normals (BDNs), **(C)** Broad Institute (WGS only) PTs, **(D)** Broad Institute BDNs, **(E)** Washington University PTs, **(F)** Washington University BDNs, **(G)** MD Anderson PTs, **(H)** MD Anderson BDNs, **(I)** Canada's Michael Smith Genome Sciences Centre PTs. PERMANOVA values inset, 999 permutations. Samples colored by cancer type within each batch.

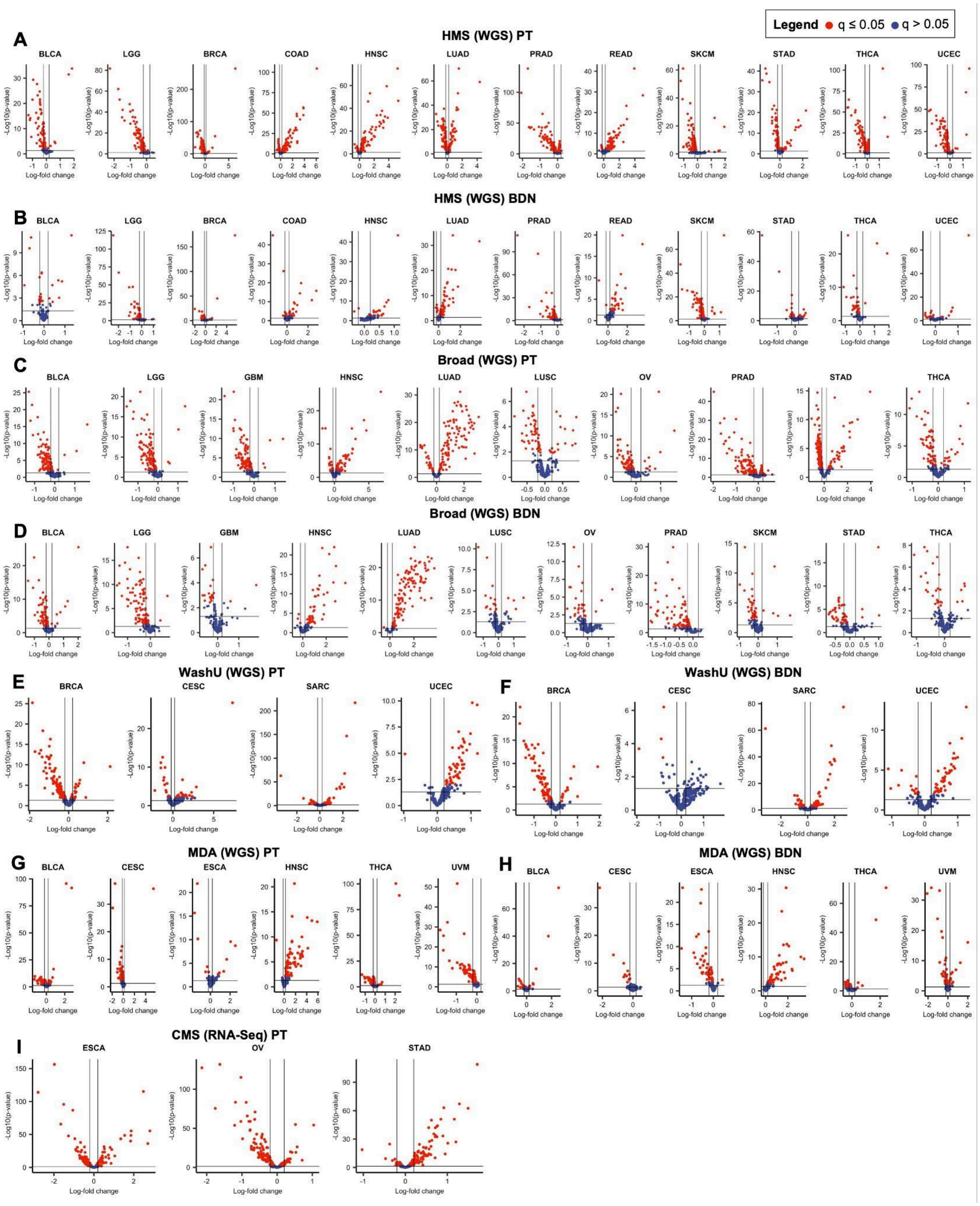

Supplementary Figure 24. Microbial differential abundances using KrakenUniq-MicrobialDB filtered genera abundances provide evidence for cancer type specific microbiomes. (A-I) Per TCGA batch, per cancer type, differential abundances by ANCOM-BC [27] using KrakenUniq-MicrobialDB-derived 294 filtered genera among **(a)** Harvard

Medical School primary tumors (PTs), **(B)** Harvard Medical School blood derived normals (BDNs), **(C)** Broad Institute PTs, **(D)** Broad Institute BDNs, **(E)** Washington University PTs, **(F)** Washington University BDNs, **(G)** MD Anderson PTs, **(H)** MD Anderson BDNs, and **(I)** Canada's Michael Smith Genome Sciences Centre PTs. Red dots denote microbes with $q \leq 0.05$. Positive log-fold changes denote microbes associated with that particular cancer type.

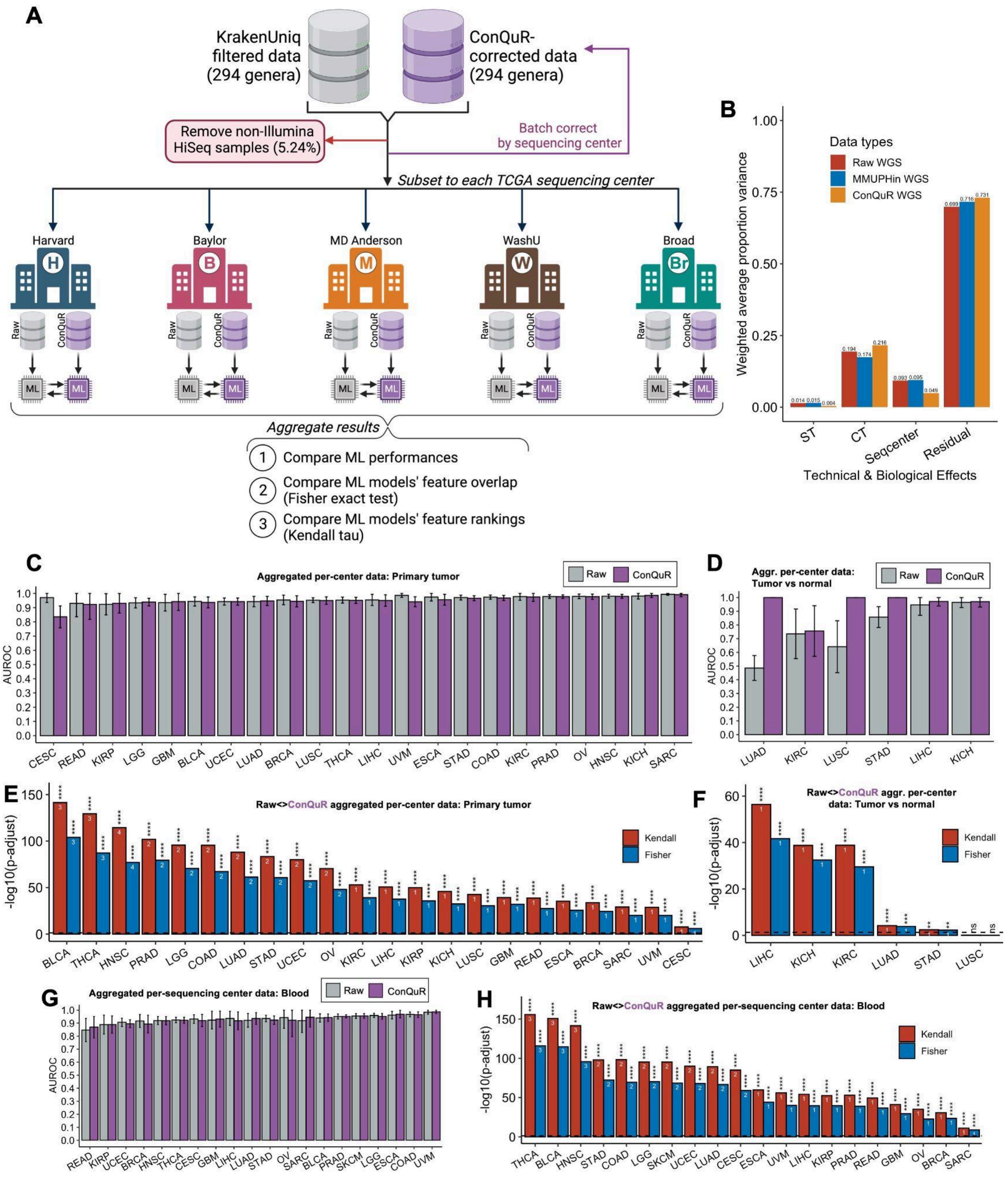

Supplementary Figure 25. Evaluation of efficacy and impact of ConQuR batch correction using KrakenUniq-MicrobialDB-derived filtered genera. (A) ConQuR batch correction was performed on WGS Illumina HiSeq-processed samples to account for sequencing center bias among 294 filtered genera abundances. Raw and corrected data were then subset to individual TCGA WGS batches to compare ML performances and feature importance similarities. **(B)**

Principal variance components analysis comparing raw (red) and ConQuR-corrected (blue) data. ST: sample type; CT: cancer type; seqcenter: TCGA sequencing center; residual denotes variance not otherwise explained by these factors. **(C)** Aggregated per-batch AUROCs for one-cancer-type-versus-all-others ML using primary tumors (PTs). **(D)** Aggregated per-batch AUROCs for tumor-versus-normal ML. **(E-F)** Aggregated and combined p-values from per-batch Fisher exact tests (blue) and Kendall tau correlations (red) across all per-batch **(E)** primary tumor and **(F)** tumor versus normal comparisons. **(G)** Aggregated per-batch AUROCs for one-cancer-type-versus-all-others ML using blood samples. **(H)** Aggregated and combined p-values from per-batch Fisher exact tests (blue) and Kendall tau correlations (red) across all per-batch blood sample comparisons. **(C-D, G)** Error bars denote 95% confidence intervals. **(E-F, H)** Inset white numbers denote the number of batches (i.e., sequencing centers) from which data derived for each particular cancer type. When p-values were combined across multiple batches, Fisher's method was used on the raw per-batch p-values, followed by Benjamini-Hochberg correction across cancer types. Logarithms are base 10.

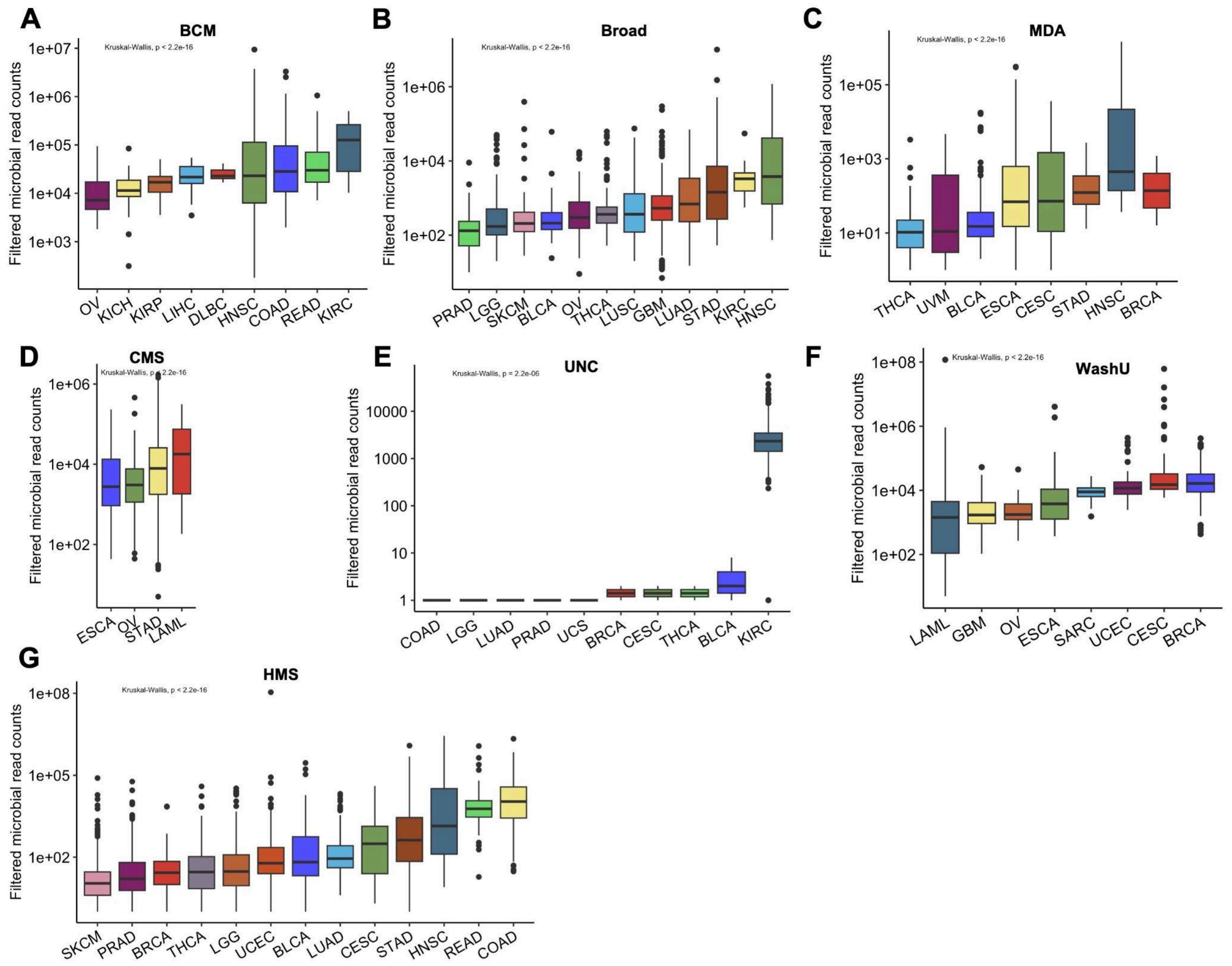

Supplementary Figure 26. Filtered microbial reads per TCGA sequencing center using the SHOGUN/Woltka pipeline against RS210-clean. (A-G) Summated RS210-clean microbial read counts from filtered 689 species across **(A)** Baylor College of Medicine (WGS), **(B)** the Broad Institute (WGS), **(C)** MD Anderson (WGS), **(D)** Canada's Michael Smith Genome Sciences Centre (RNA-Seq), **(E)** the University of North Carolina (RNA-Seq), **(F)** Washington University (WGS), and **(G)** Harvard Medical School (WGS). All samples from each center included. Kruskall-Wallis test values inset.

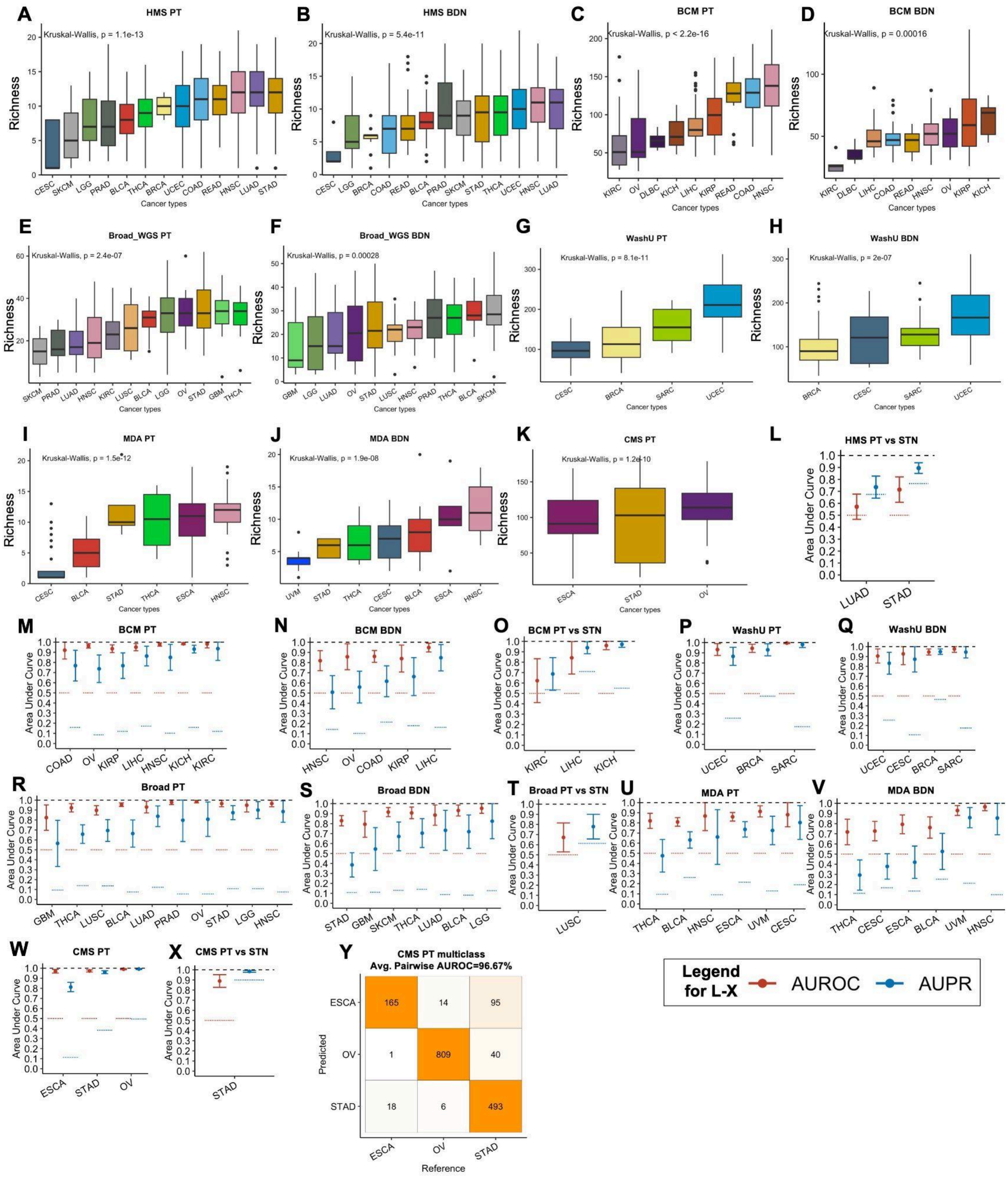

Supplementary Figure 27. Alpha diversity and machine learning (ML) reveals cancer type specific microbiomes in individual TCGA batches using RS210-clean filtered species. **(A-K)** Observed number of filtered species after rarefaction to approximately the first quartile of read counts per sequencing center and sample type (primary tumor or blood) TCGA batch among **(A)** Harvard Medical School PTs, **(B)** Harvard Medical School BDNs, **(C)** Baylor College

of Medicine primary tumors (PTs), **(D)** Baylor College of Medicine blood derived normals (BDNs), **(E)** Broad Institute (WGS only) PTs, **(F)** Broad Institute (WGS only) BDNs, **(G)** Washington University PTs, **(H)** Washington University BDNs, **(I)** MD Anderson PTs, **(J)** MD Anderson BDNs, and **(k)** Canada's Michael Smith Genome Sciences Centre PTs. Kruskal-Wallis tests inset. **(L-X)** Per batch, one-cancer-type-versus-all-others ML using PTs or BDNs, or tumor-versus-normal (PT vs STN) ML, among **(L)** Harvard Medical School, **(M-O)** Baylor College of Medicine, **(P-Q)** Washington University, **(R-T)** Broad Institute (WGS only), **(U-V)** MD Anderson, **(W-X)** Canada's Michael Smith Genome Sciences Centre (CMS). Error bars denote averages (dots) and 95% confidence intervals (brackets) of 10-fold cross-validation. Null AUPR and AUPR shown as dotted horizontal lines. **(Y)** Multiclass ML within CMS.

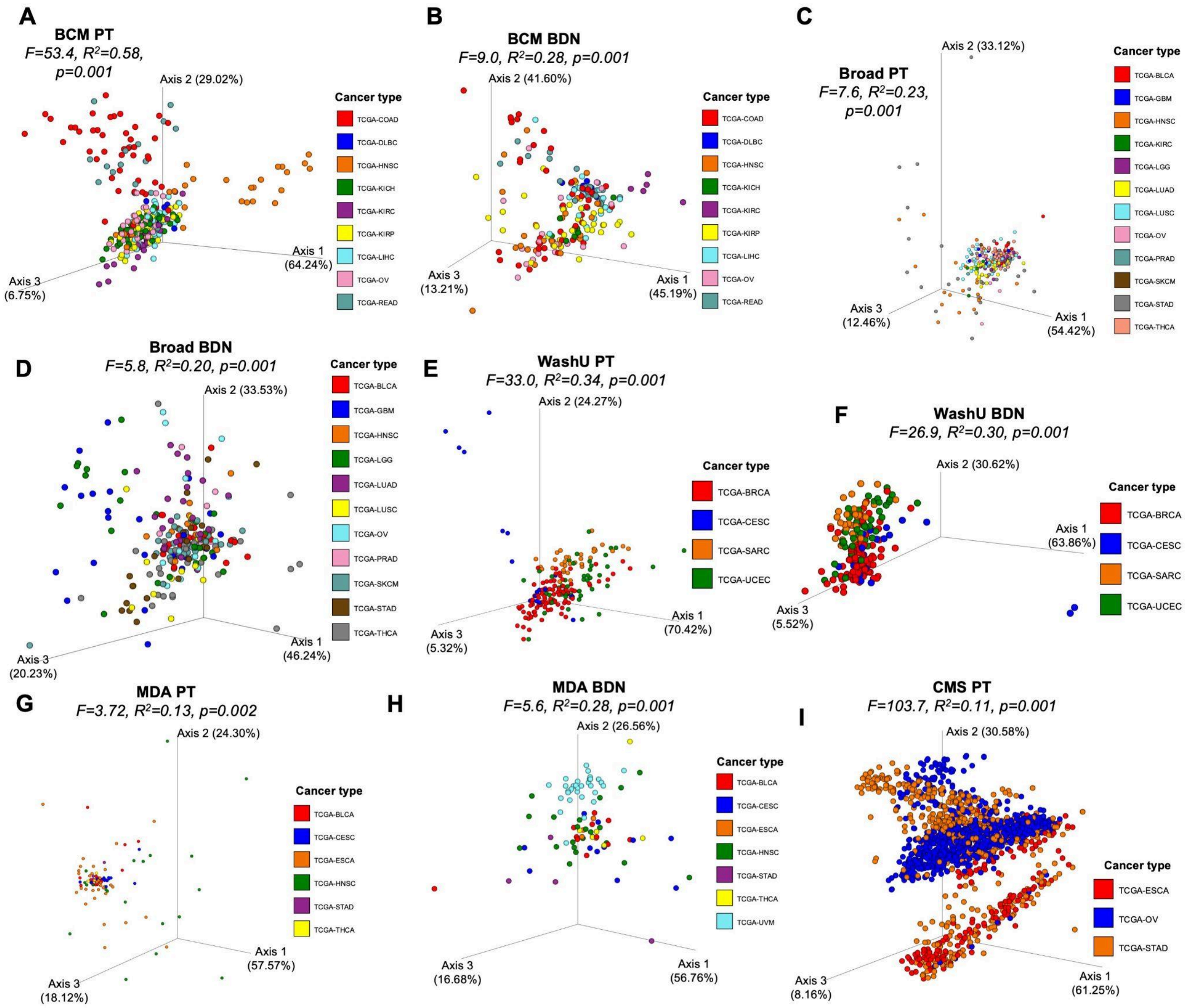

Supplementary Figure 28. Aitchison beta diversity in individual TCGA batches using RS210-clean filtered species reveals cancer type specific microbiomes. (A-I) Aitchison beta diversities calculated by RPCA [31] using RS210-clean-derived 689 filtered species among **(A)** Harvard Medical School primary tumors (PTs), **(B)** Harvard Medical School blood derived normals (BDNs), **(C)** Broad Institute (WGS only) PTs, **(D)** Broad Institute BDNs, **(E)** Washington University PTs, **(F)** Washington University BDNs, **(G)** MD Anderson PTs, **(H)** MD Anderson BDNs, **(I)** Canada's Michael Smith Genome Sciences Centre PTs. PERMANOVA values inset, 999 permutations. Samples colored by cancer type within each batch.

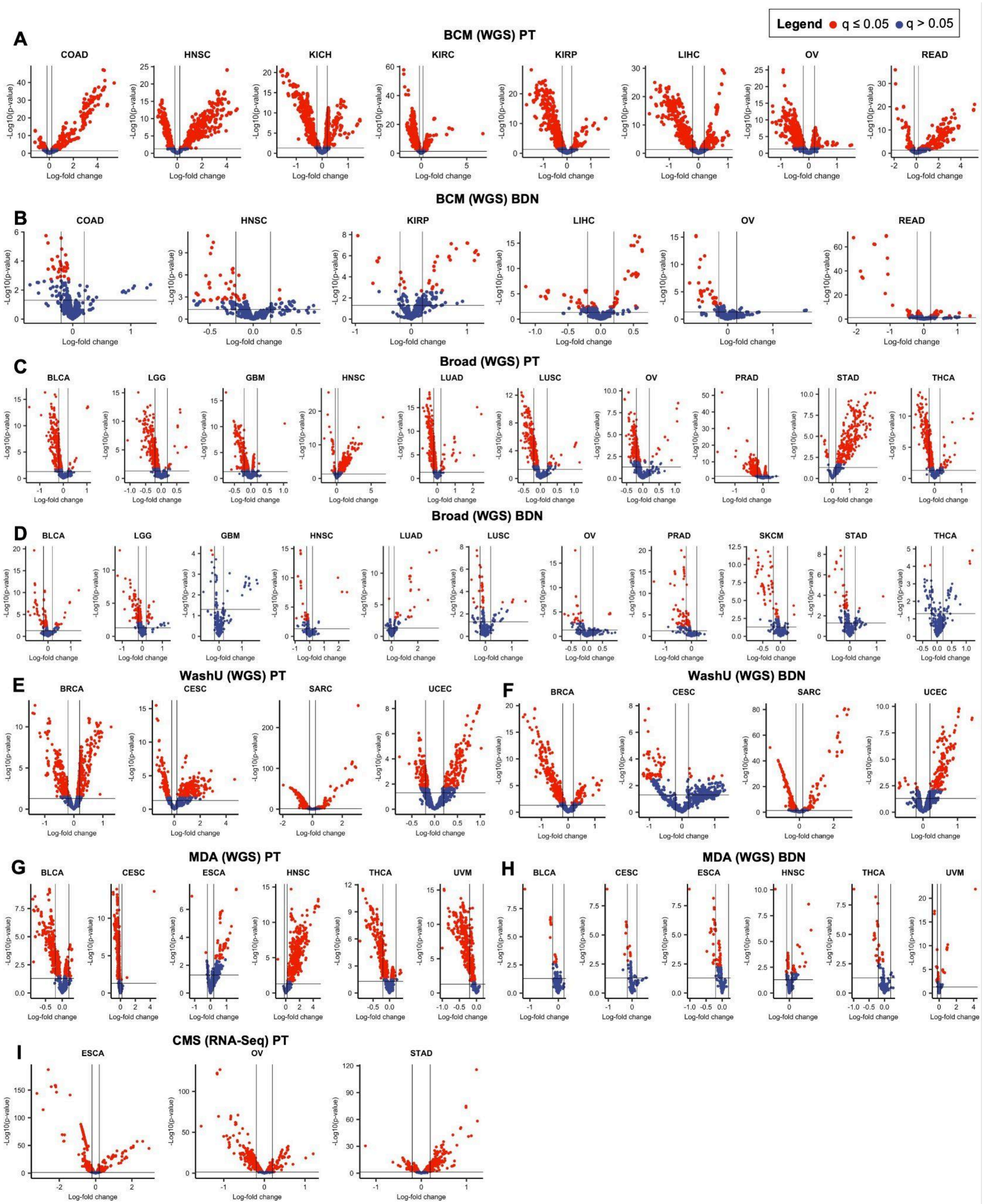

Supplementary Figure 29. Microbial differential abundances using RS210-clean filtered species abundances provide evidence for cancer type specific microbiomes. (A-I) Per TCGA batch, per cancer type, differential abundances by ANCOM-BC [27] using RS210-clean-derived 689 filtered species among **(A)** Baylor College of Medicine primary tumors

(PTs), **(B)** Baylor College of Medicine blood derived normals (BDNs), **(C)** Broad Institute PTs, **(D)** Broad Institute BDNs, **(E)** Washington University PTs, **(F)** Washington University BDNs, **(G)** MD Anderson PTs, **(H)** MD Anderson BDNs, and **(I)** Canada's Michael Smith Genome Sciences Centre PTs. Red dots denote microbes with $q \leq 0.05$. Positive log-fold changes denote microbes associated with that particular cancer type.

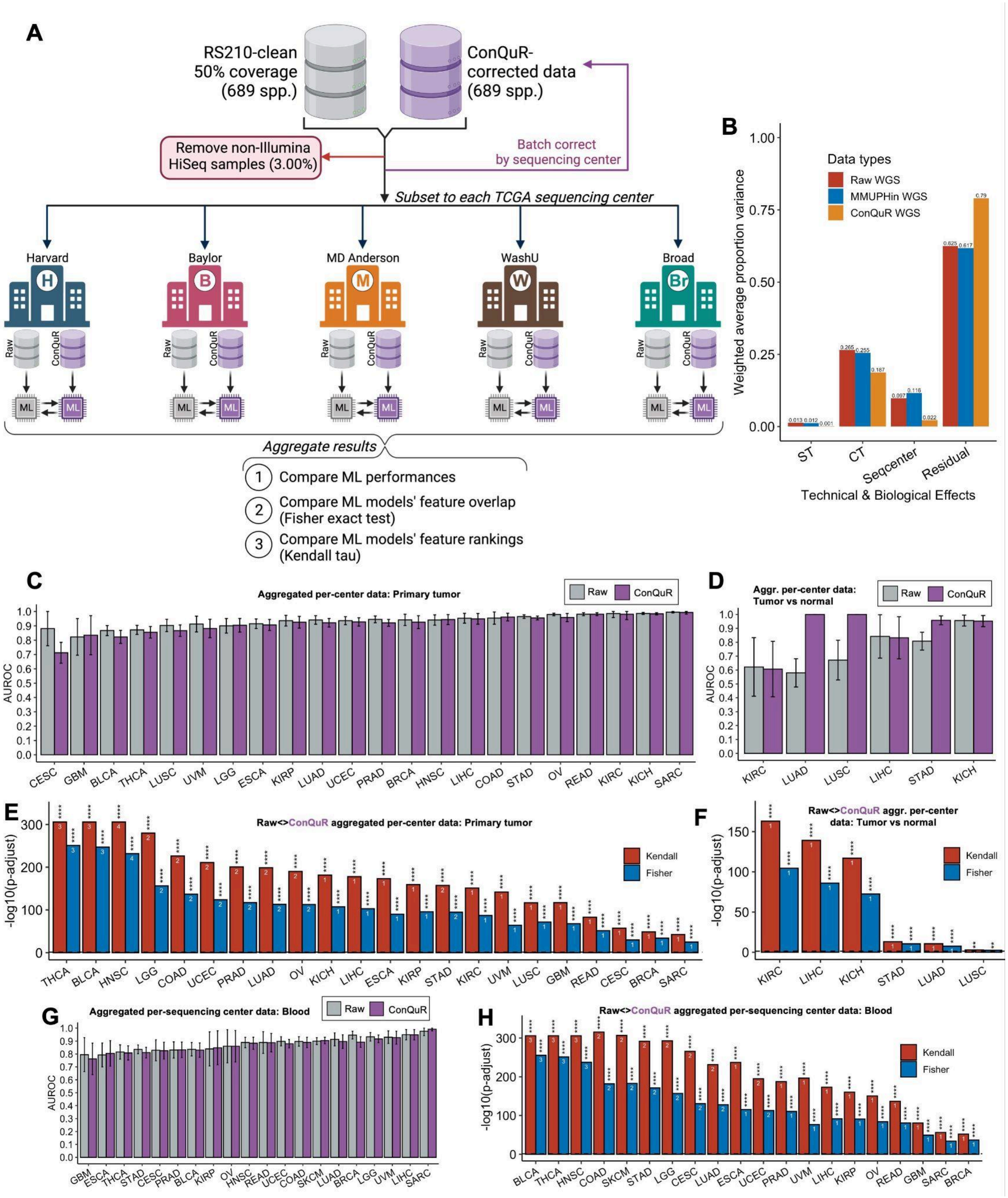

Supplementary Figure 30. Evaluation of efficacy and impact of ConQuR batch correction using RS210-clean-derived filtered species. (A) ConQuR batch correction was performed on WGS Illumina HiSeq-processed samples to account for sequencing center bias among 689 filtered species abundances. Raw and corrected data were then subset to individual TCGA WGS batches to compare ML performances and feature importance similarities. **(B)** Principal variance components analysis comparing raw (red) and ConQuR-corrected (blue) data. ST:

sample type; CT: cancer type; seqcenter: TCGA sequencing center; residual denotes variance not otherwise explained by these factors. **(C)** Aggregated per-batch AUROCs for one-cancer-type-versus-all-others ML using primary tumors (PTs). **(D)** Aggregated per-batch AUROCs for tumor-versus-normal ML. **(E-F)** Aggregated and combined p-values from per-batch Fisher exact tests (blue) and Kendall tau correlations (red) across all per-batch **(E)** primary tumor and **(F)** tumor versus normal comparisons. **(G)** Aggregated per-batch AUROCs for one-cancer-type-versus-all-others ML using blood samples. **(H)** Aggregated and combined p-values from per-batch Fisher exact tests (blue) and Kendall tau correlations (red) across all per-batch blood sample comparisons. **(C-D, G)** Error bars denote 95% confidence intervals. **(E-F, H)** Inset white numbers denote the number of batches (i.e., sequencing centers) from which data derived for each particular cancer type. When p-values were combined across multiple batches, Fisher's method was used on the raw per-batch p-values, followed by Benjamini-Hochberg correction across cancer types. Logarithms are base 10.

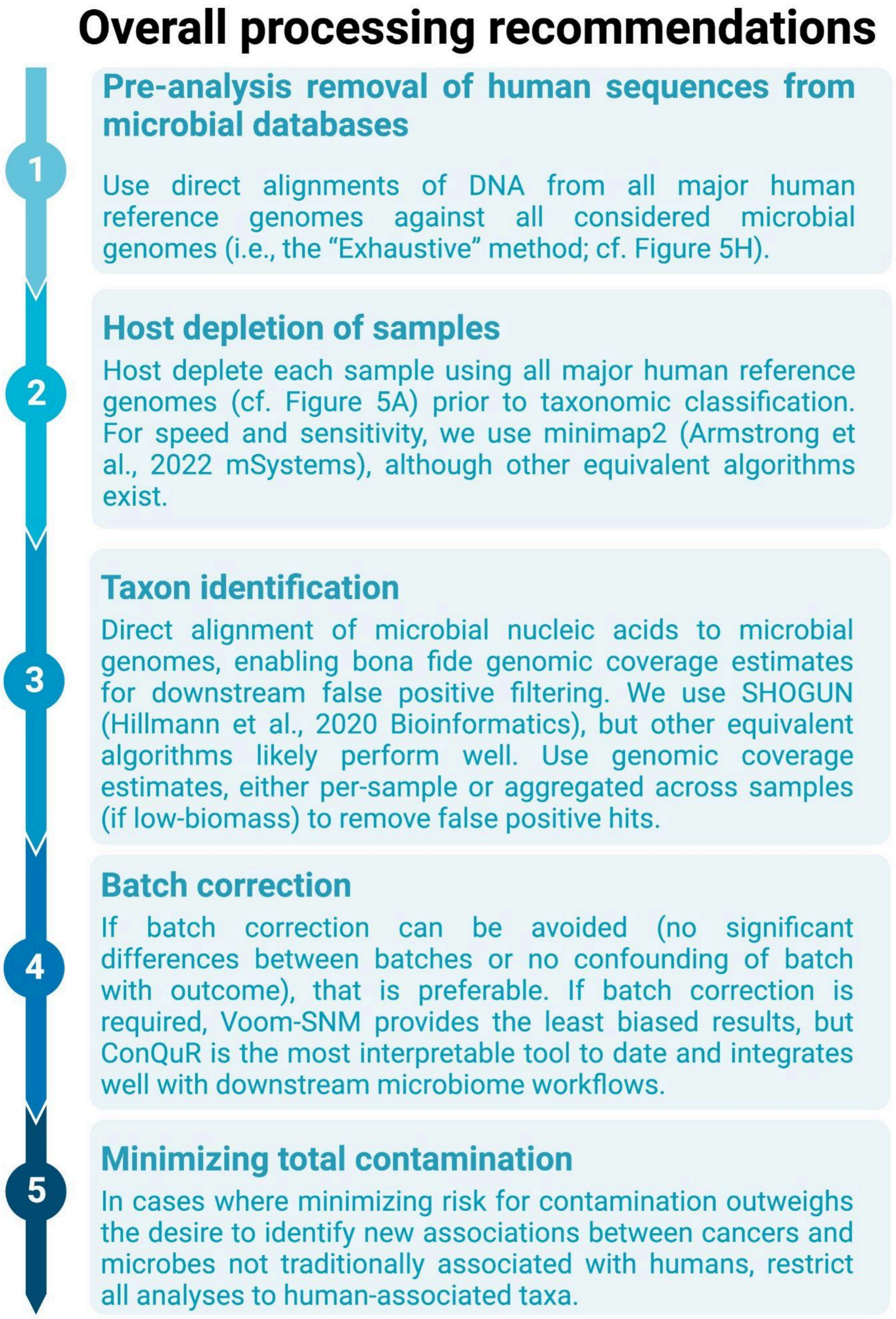

Supplementary Figure 31. Recommendations for each step of the cancer microbiome workflow.

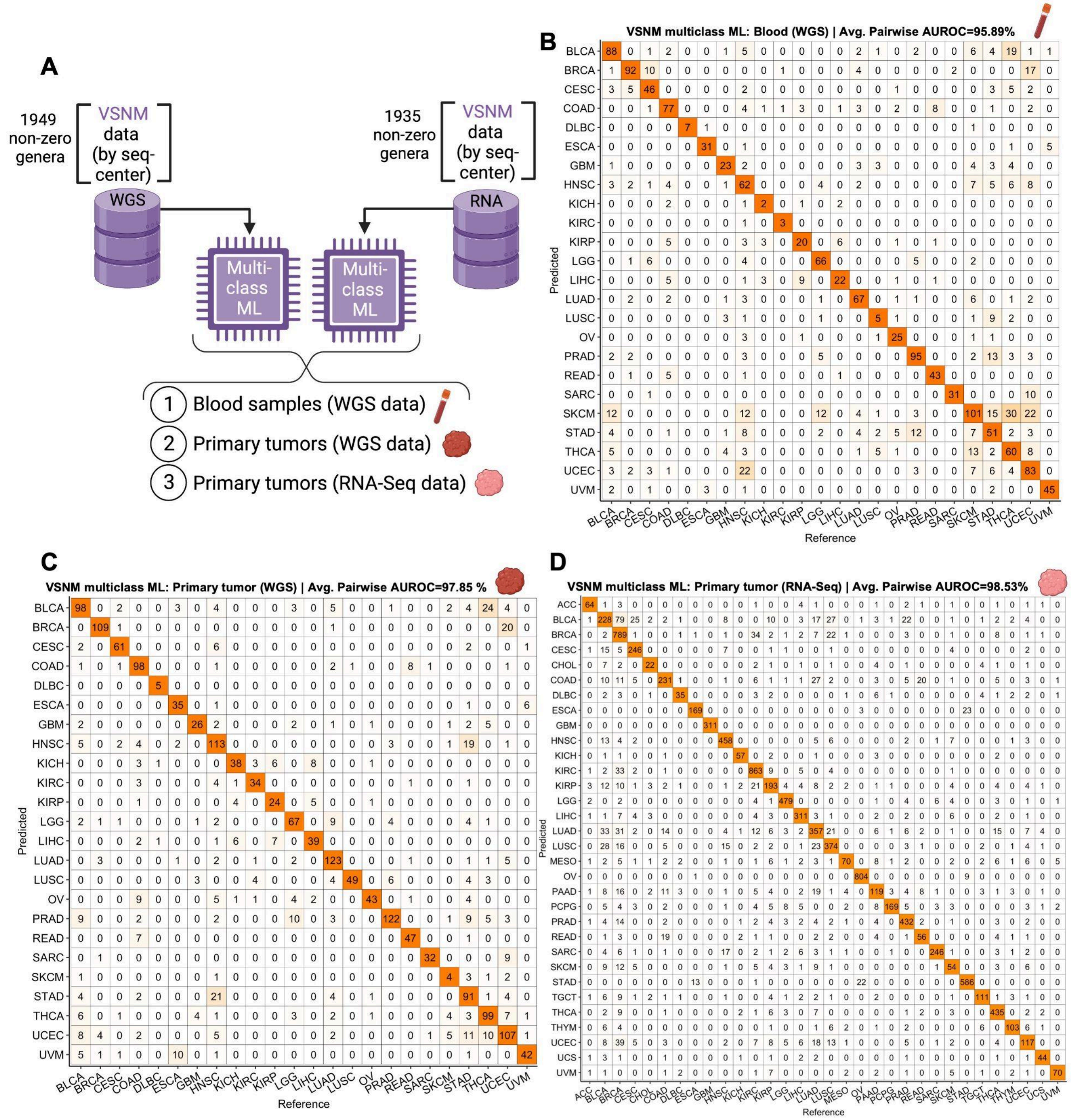

Supplementary Figure 32. VSNM-corrected, full genera abundances demonstrate pan-cancer discrimination among blood and primary tumor samples across dozens of cancer types. (A) Diagram of data sources for the multiclass ML. To compare VSNM to ConQuR, which was limited to correcting one batch variable, sequencing center bias was corrected within WGS and RNA-Seq groups after subsetting analyses to Illumina HiSeq-processed samples. Multiclass ML was run independently on the VSNM-corrected WGS and RNA-Seq groups; of note, blood samples were only in the WGS group. **(B)** Multiclass gradient boosting ML across 24 cancer types using all blood samples in TCGA. Average pairwise AUROC is denoted above the confusion matrix. No information rate: 9.1%, mean balanced accuracy: 82.4%. **(C)** Multiclass gradient boosting ML across 24 cancer types using all

WGS primary tumors in TCGA. Average pairwise AUROC is denoted above the confusion matrix. No information rate: 8.5%, mean balanced accuracy: 86.8% **(D)** Multiclass gradient boosting ML across 32 cancer types using all RNA-Seq primary tumors in TCGA. Average pairwise AUROC is denoted above the confusion matrix. No information rate: 10.7%, mean balanced accuracy: 89.7%. (**B-D**) P-values are all less than 2.2×10^{-16} for comparing the no information rate to the observed accuracy. See **Supplementary Fig. 1C** for list of TCGA cancer type abbreviations.

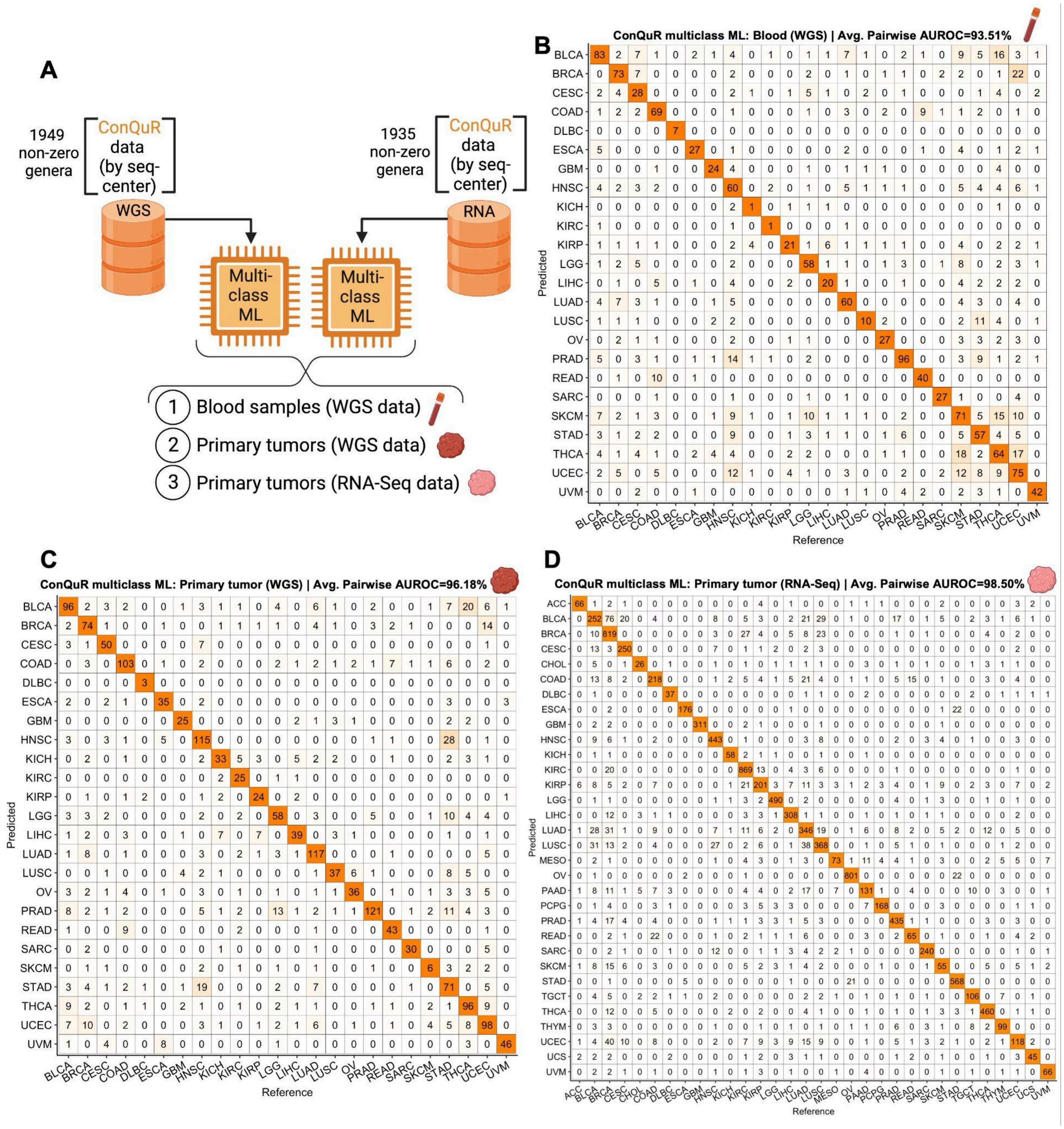

Supplementary Figure 33. ConQuR-corrected, full genera abundances demonstrate pan-cancer discrimination among blood and primary tumor samples across dozens of cancer types. (A) Diagram of data sources for the multiclass ML. Since ConQuR was limited to correcting one batch variable, sequencing center bias was corrected within WGS and RNA-Seq groups after subsetting analyses to Illumina HiSeq-processed samples. Multiclass ML was run independently on the ConQuR-corrected WGS and RNA-Seq groups; of note, blood samples were only in the WGS group. **(B)** Multiclass gradient boosting ML across 24 cancer types using all blood samples in TCGA. Average pairwise AUROC is denoted above the confusion matrix. No information rate: 9.1%, mean balanced accuracy: 79.3%. **(C)** Multiclass gradient boosting ML across 24 cancer types using all WGS primary tumors in TCGA. Average pairwise AUROC

is denoted above the confusion matrix. No information rate: 8.5%, mean balanced accuracy: 83.1% **(D)** Multiclass gradient boosting ML across 32 cancer types using all RNA-Seq primary tumors in TCGA. Average pairwise AUROC is denoted above the confusion matrix. No information rate: 10.7%, mean balanced accuracy: 90.3%. (**B-D**) P-values are all less than 2.2×10^{-16} for comparing the no information rate to the observed accuracy. See **Supplementary Fig. 1C** for list of TCGA cancer type abbreviations.

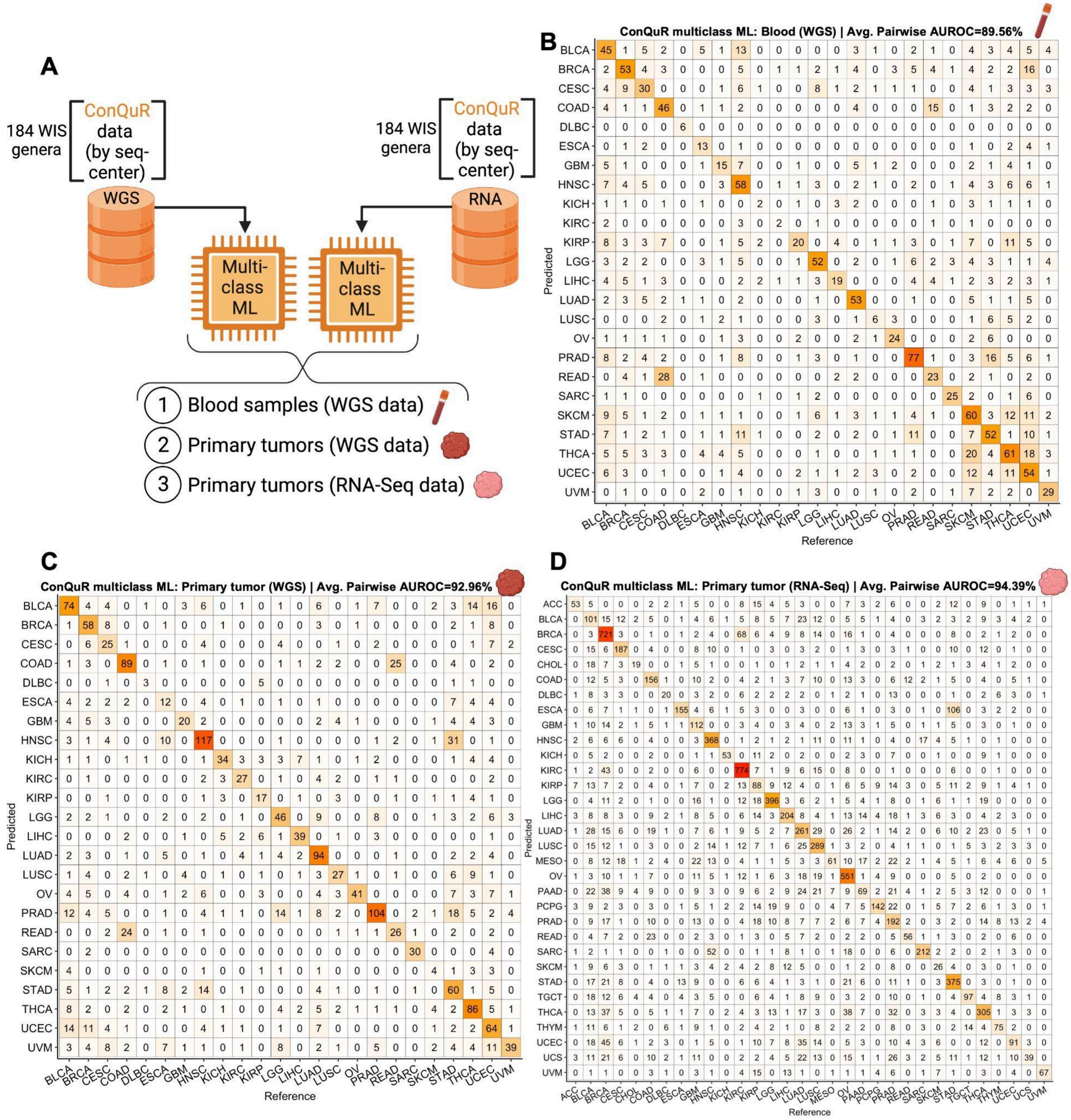

Supplementary Figure 34. Alternative version of Fig. 3 using a single color gradient for confusion matrices. We carefully note that single color gradients on confusion matrices with imbalanced classes can make it falsely appear as if less prevalent cancer types consistently have low performance. However, since some readers may prefer this color scheme, we have presented it in addition to confusion matrices with colored diagonals.

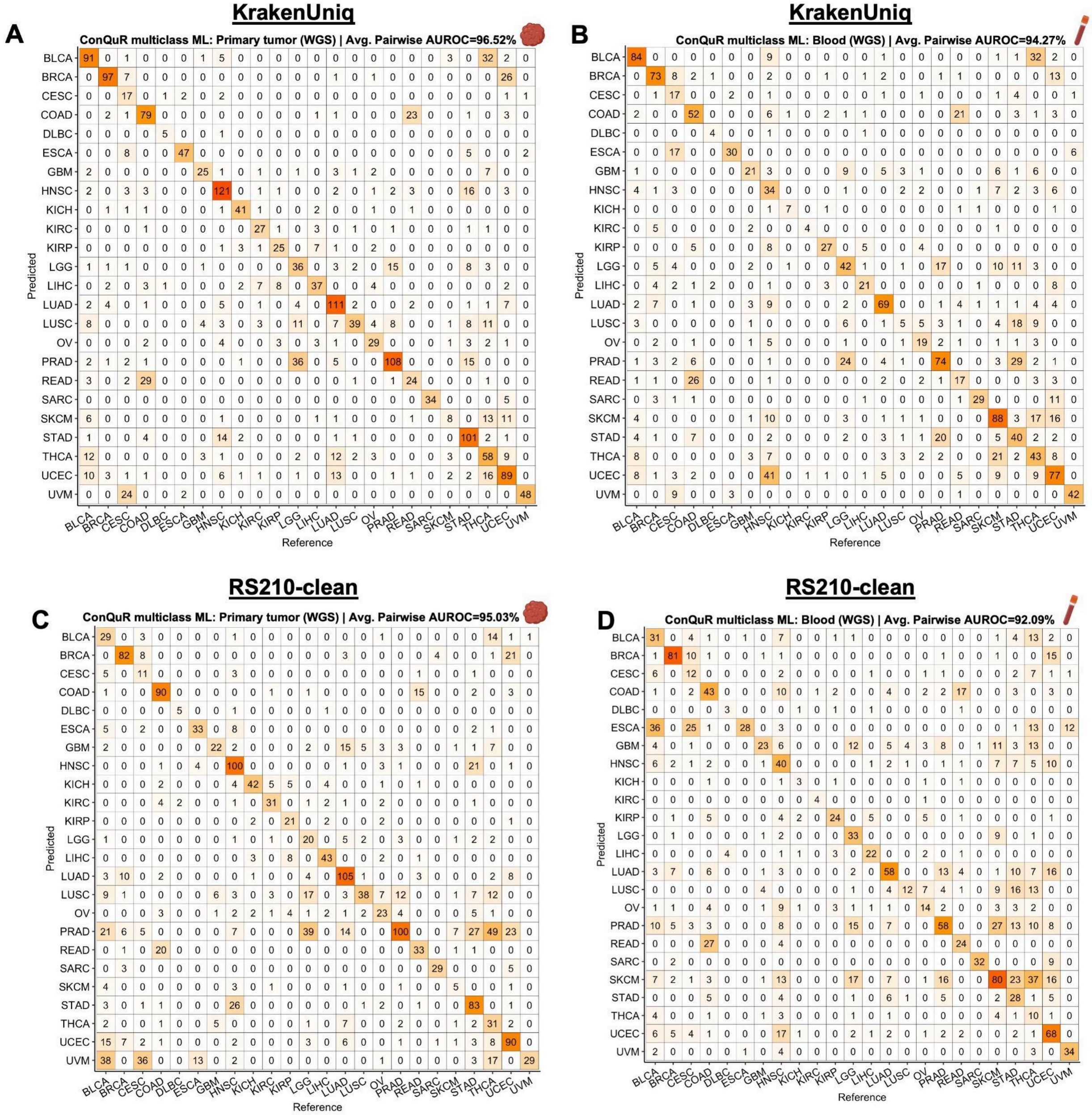

Supplementary Figure 35. Alternative versions of Fig. 6J-K and Fig. 8J-K using a single color gradient for confusion matrices. (**A-B**) Alternative coloring scheme of Fig. 6J-K. (**C-D**) Alternative coloring scheme of Fig. 8J-K. We carefully note that single color gradients on confusion matrices with imbalanced classes can make it falsely appear as if less prevalent cancer types consistently have low performance. However, since some readers may prefer this color scheme, we have presented it in addition to confusion matrices with colored diagonals.

Supplementary Table Legends

Table S1: A section-by-section response to the claims raised by Gihawi et al.
Table S2: A high-level summary of our analyses.
Table S3: Genomes in the original Kraken database with ≥1 Conterminator-detected human sequence.
Table S4: Genomes in the WoLr1 database with ≥1 Conterminator-detected human sequence.
Table S5: List of KrakenUniq-MicrobialDB 294 filtered genera.
Table S6: Aggregate genome coverages of human-associated, non-viral RS210-clean species.
Table S7: List of RS210-clean filtered unique species having ≥50%, ≥75%, or ≥90% aggregate genome coverage.
Table S8: T2T-KrakenUniq-MicrobialDB filtered genera abundances in TCGA (full set).
Table S9: Metadata for T2T-KrakenUniq-MicrobialDB filtered genera in TCGA (full set).
Table S10: hg38-KrakenUniq-MicrobialDB filtered genera abundances in TCGA (overlapping samples with T2T and HPRC).
Table S11: T2T-KrakenUniq-MicrobialDB filtered genera abundances in TCGA (overlapping samples with hg38 and HPRC).
Table S12: HPRC-KrakenUniq-MicrobialDB filtered genera abundances in TCGA (overlapping samples with hg38 and T2T).
Table S13: RS210-clean filtered genome-level abundances in TCGA (≥50% genome coverage).
Table S14: Metadata for RS210-clean filtered data in TCGA (≥50% genome coverage).
Table S15: List of multiclass ML features derived on original Kraken, ConQuR-corrected, WIS-overlapping features, related to Fig. 3.
Table S16: List of multiclass ML features derived on original Kraken, VSNM-corrected, WIS-overlapping features, related to Supplementary Fig. 9.
Table S17: List of multiclass ML features derived on KrakenUniq, ConQuR-corrected features, related to Fig. 6J-K and Supplementary Fig. 22Y.
Table S18: List of multiclass ML features derived on RS210-clean, ConQuR-corrected features, related to Fig. 8J-K and Supplementary Fig. 27Y.
Table S19: List of per-cancer type, per-taxon differential abundance derived using ANCOM-BC on ConQuR-corrected, KrakenUniq data, related to Figure 6J-K.
Table S20: List of per-cancer type, per-taxon differential abundance derived using ANCOM-BC on ConQuR-corrected, RS210-clean data, related to Figure 8J-K.
Table S21: List of multiclass ML features derived on original Kraken, VSNM-corrected, full features, related to Table S16.
Table S22: List of multiclass ML features derived on original Kraken, ConQuR-corrected, full features, related to Table S15.
Table S23: Overlap statistics between multiclass ML models developed on Kraken data (VSNM or ConQuR-corrected) from Poore & Kopylova et al. 2020 vs. that from RS210-clean with ConQuR-correction.
