# Supplementary material for: Robustness of cancer microbiome signals over a broad range of methodological variation
Source: Oncogene. 2024 Feb 23;43(15):1127–48. doi: 10.1038/s41388-024-02974-w (PMC10997506; doi:10.1038/s41388-024-02974-w)
Supplement: Supplementary file 2 — Supplementary Tables [file 41388_2024_2974_MOESM2_ESM.zip › Tables copy/Table S1.docx]

| **Table S1. A section-by-section response to the claims raised by Gihawi et al.** [1] | | |
| --- | --- | --- |
| **Section(s) in Gihawi et al., in order of appearance** | **Summary of claim** | **Our response** |
| **Filtered "non-human" reads contained millions of human reads** | Certain reads that are unmapped to GRCh37/38 in TCGA map to T2T-CHM13v2.0. | We agree with this issue, and have demonstrated similar results in work since the 2020 paper using newer human genome references [2].  The resources needed to assess this claim (T2T-CHM13v2.0) were not available at the time of the original analysis.  The cancer-specific microbial signatures remain even with newer genome references and are robust to this issue. |
| **Bacterial read counts were inflated by many orders of magnitude** | The presence of human reads that were not detected using hg19 (and are only detected with T2T-CHM13v2.0), combined with known human contamination of microbial reference and the lack of human genome in the Kraken database would result in wrongly assigning human reads as microbial.  The presence of vector contamination would result in wrongly assigning reads as microbial. | This claim does not appear supported by any analysis in Gihawi et al. The databases in the original Poore et al. paper are publicly available (WoLr1) or well described (original Kraken database), and we are unsure why the concern of human and vector sequence was not explicitly evaluated by the authors. **Fig. 4A** shows that human contamination in the databases used by the original paper was minimal.  This claim additionally ignores the analysis in the original paper performed with SHOGUN via bowtie2, which would not be affected by the lack of a human genome in the database. Simulations in **Fig. 4H-J** show that the number of reads that were misclassified in this analysis because of an “error” in the database is minimal.  The following evidence shows vector contamination is not a major source of signal: (1) Many genomes including those independently associated with cancer types (e.g., *Fusobacterium nucleatum*) exhibit very high coverage (**Fig. 5M**), whereas if the signal were driven by vector sequences, the anticipated effect would be low coverage spanning only the vector-contaminated regions. (2) The KrakenUniq MicrobialDB contains UniVec sequences as reported by Gihawi et al., and the feature tables produced by Gihawi et al. assessed using KrakenUniq against this database exhibit robust cancer-specific microbial signals (**Supplementary Fig. 20**).  Gihawi et al.’s claims regarding database contamination with human and vector sequence data are not supported by analyzing the databases utilized in the original Poore et al. 2020 study. Our analysis of those databases here shows that the claim, although theoretically possible, is not supported by the data. |
| **Re-analysis of bladder cancer samples.**  **Re-analysis of head and neck cancer and breast cancer samples.**  **Nearly all of the raw numbers in the Poore et al. study are incorrect and far too high.**  **How human reads create the false appearance of bacteria.** | A subset of cancer samples analyzed in the original Poore et al. paper were filtered using T2T-CHM13 and mapped to a Kraken database that contains only complete microbial genomes and a human genome. Analysis shows:   - Substantially lower counts of many taxa and samples compared to the original paper were observed. - 1-5% of reads mapped to the human genome. - A single sample (s2707) mapped to a database containing all available genomes from 2 genera shows higher read counts than if first depleted using T2T-CHM13, or mapped to a reference containing it. | The claim by Gihawi et al. is that the higher bacterial counts in the original paper stem from an error in the database used. However, their analysis conflates a “corrected” database with improved host-depletion using non-contemporaneous resources. Our analysis in **Fig. 5** and **Supplementary Fig. 18-19** shows that this reduction in bacterial counts results from host depletion with T2T-CHM13, a resource that was not available at the time of the original analysis.  This analysis is further conflated by restricting the database to complete genomes. The diversity of complete genomes is significantly lower, making a reduction in mapping unsurprising, especially in less-studied ecosystems [3,4]. This observation does not directly demonstrate human contamination of the database used. Our analysis in **Fig. 4A** shows that human contamination of databases used in the original analyses was minimal.  We demonstrate that following contemporary state-of-the-art practices not available at the time of our original study, near-complete accounting for human reads is possible (**Supplementary Fig. 18E-F**).  The analysis of a single sample, s2707, similarly conflates depletion with T2T-CHM13, a non-contemporaneous resource, with a database error.  We therefore disagree with the claim that the microbial database is substantially contaminated with human reads, and that these human reads lead to tumor-specific microbial signatures. |
| **Normalization of the reads erroneously created a distinct signature for each cancer** | This analysis demonstrates that certain features, which were sparse (mostly 0) in the raw data in the original paper, were assigned nonzero values by the Voom-SNM transformation. These transformed values then exhibit strong associations with cancer types. It is claimed that these values are artificial data leakage [5], and account for the tumor-specific microbial signatures we report. | Batch-correction methods such as Voom-SNM apply a transformation to the entire dataset. We agree that it impacts interpretability, in that the values of every feature (sparse and non-sparse) are modified. This is typical of normalization and batch-correction methods, including microbiome-specific tools like ConQuR. However, this does not imply that the transformation leads to data leakage that could provide an unfair advantage to a classifier. The claim that the signal is artificial is not supported by any specific analysis done by Gihawi et al. that we are aware of. Critically, Voom-SNM was blinded to the labels (cancer types), so it did not have means to consider the labels during its application.  If Voom-SNM were introducing an artificial signal (data leakage), then running analyses on permuted data - scrambled metadata labels or shuffled counts - would still yield accurate classifiers. Our analyses in **Supplementary Figs. 4-5, 7-8** show that this is not the case.  We further show that an analysis without Voom-SNM done within batches shows similar accuracy to analysis with Voom-SNM (**Fig. 1-2**; **Supplementary Fig. 2-5**) and assigns similar feature importances (**Fig. 1-2**; **Supplementary Fig. 2-3**). This demonstrates that Voom-SNM is not the source of these signatures.  An analysis with ConQuR, a new, microbiome-specific batch-correction method that is independent of Voom-SNM, performs similarly to both Voom-SNM transformed data and to raw data (within batch) (**Fig. 2-3; Supplementary Fig. 6-8**). This analysis also rules out Voom-SNM as the source of the signatures.  We therefore stand by the use of Voom-SNM as a valid technique for dealing with microbiome batch effects. |
| **Replicating highly accurate classifiers on information-free raw data demonstrates flaws in the normalization process.** | The claim in this section is that the Voom-SNM transformation “invents” data in an information-free setting, which is then used by classifiers to make accurate predictions. This section also claims that the results obtained using blood WGS sequencing data in the original paper are flawed. | For a Voom-SNM transformation $V()$, input data $X$, and an operation that subsets $X$ to the originally-zero subset of the data $X_{[...]}$, Gihawi et al. make the claim that $V(X_{[...]})$, an application of Voom-SNM in an information-free scenario, yields accurate predictors. This is not, however, the analysis they performed. As Gihawi et al. write: “We then populated each cell in the empty matrix with its corresponding value from the Voom-SNM normalized data.”, the transformation performed is instead $V({X)}_{[...]}$- i.e., a simple subsetting of the original transformed matrix. This is not an analysis of information-free raw data, but instead the transformation is performed on the entire data, which uses the information from all non-empty features.  Furthermore, Gihawi et al. offer no evidence of flaws with the blood WGS sequencing analysis.  We therefore see these simulations as irrelevant to our application of Voom-SNM, although we agree that like many data processing techniques it can yield invalid results if misapplied. |
| **Discussion** | This section claims that the association between microbes and cancer types reported in the study is a fiction. | Gihawi et al. make this claim without using their filtered data to investigate associations with cancer types. When we perform this analysis, using their filtered data, we find strong signals of a tumor-specific microbiome as in the original analysis (**Supplementary Fig. 20**). Application of their pipeline to the rest of TCGA also shows cancer type-specific microbiomes (**Fig. 6; Supplementary Fig. 21-25**), with consistent signatures across varying degrees of host depletion (**Fig. 7**). Using many rigorous host-depletion and database cleaning methods, some more stringent than those proposed by Gihawi et al., we show that even with conservative state-of-the-art tools introduced after our 2020 paper, a strong cancer type association still exists (**Fig. 8; Supplementary Fig. 26-30**) |

**References**

1. Gihawi A, Ge Y, Lu J, Puiu D, Xu A, Cooper CS, et al. Major data analysis errors invalidate cancer microbiome findings. MBio. 2023; e0160723. doi:10.1128/mbio.01607-23

2. Narunsky-Haziza L, Sepich-Poore GD, Livyatan I, Asraf O, Martino C, Nejman D, et al. Pan-cancer analyses reveal cancer type-specific fungal ecologies and bacteriome interactions. Cell. 2022;185: 3789–3806.e17. doi:10.1016/j.cell.2022.09.005

3. Almeida A, Nayfach S, Boland M, Strozzi F, Beracochea M, Shi ZJ, et al. A unified catalog of 204,938 reference genomes from the human gut microbiome. Nat Biotechnol. 2021;39: 105–114. doi:10.1038/s41587-020-0603-3

4. Saheb Kashaf S, Proctor DM, Deming C, Saary P, Hölzer M, NISC Comparative Sequencing Program, et al. Integrating cultivation and metagenomics for a multi-kingdom view of skin microbiome diversity and functions. Nat Microbiol. 2022;7: 169–179. doi:10.1038/s41564-021-01011-w

5. Whalen S, Schreiber J, Noble WS, Pollard KS. Navigating the pitfalls of applying machine learning in genomics. Nat Rev Genet. 2022;23: 169–181. doi:10.1038/s41576-021-00434-9
