# Supplementary material for: Robustness of cancer microbiome signals over a broad range of methodological variation
Source: Oncogene. 2024 Feb 23;43(15):1127–48. doi: 10.1038/s41388-024-02974-w (PMC10997506; doi:10.1038/s41388-024-02974-w)
Supplement: Supplementary file 2 — Supplementary Tables [file 41388_2024_2974_MOESM2_ESM.zip › Tables copy/Table S2.docx]

| **Table S2. A high-level summary of our analyses.** | | | | | |
| --- | --- | --- | --- | --- | --- |
| **Claims \\\\\\\\**  **Response** | **Relevant figures** | **Reduced read counts** | **DB Error** | **Batch correction error** | **Microbial contamination** |
| Many tools used by Gihawi et al. were not available at time of original publication. | **Supplementary Fig. 1** | T2T-CHM13 is non-contemporaneous | MicrobialDB is non-contemporaneous; Conterminator was published after the original paper. | Voom-SNM was the most appropriate batch-correction method during original analyses. |  |
| Human contamination in databases used in original analyses was minimal and did not affect conclusions. | **Fig. 4; Supplementary Fig. 16; Tables S3-S4.** |  | Little human contamination found in databases used in original analysis (**Fig. 4A**).  Classification accuracy is preserved after removing human contamination from original databases (**Fig. 4B-G**; **Supplementary Fig.** **16**).  Results hold for Kraken (**Fig. 4B-D**; **Supplementary Fig.** **16A-C**) and SHOGUN (**Fig. 4E-G**; **Supplementary Fig.** **16D-F**). | Accuracy preserved within batch (**Fig. 4B-G**; **Supplementary Fig. 16**). |  |
| Human contamination in databases used in original analyses was minimal and did not affect conclusions; accounting for microbial contamination also maintains the same conclusions. | **Supplementary Fig. 17A-G**. |  | Classification accuracy is preserved after removing human contamination from original databases (**Supplementary Fig. 17A-G**). | Accuracy preserved within batch (**Supplementary Fig. 17A-G**). | This analysis was performed after filtering to microbes present in WIS analysis (**Supplementary Fig. 17A-G**). |
| Original analysis with SHOGUN was not sensitive to the lack of human genome in reference. | **Fig. 4H-J**. |  | Lack of human genome in reference did not affect original analyses with SHOGUN (**Fig. 4H-J**). |  |  |
| Host depletion with non-contemporaneous tools is responsible for the reduction in read counts observed by Gihawi et al. | **Fig. 5A-G**; **Supplementary Fig. 18-19**; **Tables S10-12** | Microbial read fraction retained with successive filtering steps (**Fig. 5D, G**).  Filtering human reads with non-contemporaneous tools drives reduced read counts (**Fig. 5B, E-F**; **Supplementary Fig. 18A, 19C-D**).  After sequential host read filtering and highly sensitive database host depletion with Exhaustive (**Fig. 5H-K**; **Supplementary Fig. 18B-D**), human reads from “unseen” individuals do not align to the database (**Supplementary Fig. 18E-F**).  Remaining microbial reads cover hundreds of microbial genomes well (**Fig. 5L-M**; **Supplementary Fig. 19C**; **Table S3-7**) | |  |  |
| Filtering and mapping pipeline used by Gihawi et al. (T2T-KrakenUniq) shows a unique cancer-microbiome signal. | **Fig. 6**; **Supplementary Fig. 20-25** | The non-contemporaneous pipeline used by Gihawi et al. nevertheless generates a cancer-specific microbial signature (**Fig. 6**; **Supplementary Fig. 20-25**). | | Classifiers remain accurate within most batches (**Fig. 6D-E**; **Supplementary Fig. 22L-Y**).  Classifiers remain accurate using ConQuR, a non-contemporaneous batch correction method (**Fig. 6J-K**; **Supplementary Fig. 25**).  ConQuR- and non-corrected-based classifiers assign similar feature importances (**Supplementary Fig. 25**) |  |
| Different levels of host depletion do not affect classification performance | **Fig. 7**. | Classifiers built using data filtered with GRCh38, T2T-CHM13, and HPRC show similar accuracy (**Fig. 7A-D**).  These classifiers also assign similar feature importances (**Fig. 7E-J**). |  | Classifiers are accurate within batches (**Fig. 7**). |  |
| The driving taxa found by different variants of the pipeline are highly correlated. | **Fig. 1-2, 7; Supplementary Fig. 2-3, 6F-H, 19-20, 25, 30** | Taxa are highly consistent at different levels of human depletion (**Fig. 7**) | Taxa are highly consistent across WGS and RNA-Seq (**Supplementary Fig. 19D**). Taxa are highly consistent in Gihawi et al. processed data across BDN and PT samples (**Fig. 20**). | Taxa are highly consistent with and without batch correction and across methods (**Fig. 1-2; Supplementary Fig. 2-3, 6F-H, 25, 30**). |  |
| Best-effort for host depletion and database filtering (TCGA-RefSeq) shows a unique cancer microbiome. | **Fig. 8; Supplementary Fig. 26-30** | A best-effort pipeline that depletes host reads with comprehensive references and filters reveals a cancer-specific microbial signature (**Fig. 8; Supplementary Fig. 26-30**). | | Classifiers remain accurate within most batches (**Fig. 8D-E; Supplementary Fig. 27L-Y**).  Classifiers remain accurate using ConQuR, a non-contemporaneous batch correction method (**Fig. 8J-K; Supplementary Fig. 30**).  ConQuR- and non-corrected-based classifiers assign similar feature importances (**Supplementary Fig. 30**) |  |
| Voom-SNM did not create an artificial tumor-type specific tag | **Fig. 1; Supplementary Fig. 2-5** |  |  | Classifiers based on Voom-SNM have equivalent performance to classifiers based on raw data that are applied within batches (**Fig. 1; Supplementary Fig. 2-3**).  Classifiers based on Voom-SNM assign similar feature importances as classifiers based on raw data that are applied within batches (**Fig. 1; Supplementary Fig. 2-3**).  Classifiers based on permuted data show little to no signal (**Supplementary Fig. 4-5**). |  |
| Voom-SNM shows equivalent results to ConQuR | **Fig. 2; Supplementary Fig. 6-8** |  |  | Voom-SNM and ConQuR show similar batch-correction performance (**Supplementary Fig. 6A-B**).  Classifiers based on Voom-SNM, ConQuR, and raw data had equivalent accuracies within batches (**Fig. 2; Supplementary Fig. 6**).  Classifiers based on Voom-SNM and ConQuR use similar features as classifiers based on raw data that are applied within batches (**Fig. 2; Supplementary Fig. 6**).  Classifiers based on permuted data show little to no signal (**Supplementary Fig. 7-8**). | This analysis was performed after filtering to microbes present in WIS analysis (**Fig. 2; Supplementary Fig. 6-8**). |
| Multi-class predictors applied to data controlling for microbial contamination reproduce original conclusions | **Fig. 3; Supplementary Fig. 9** |  |  | Multi-class classifiers based on non microbially-contaminated data processed with ConQuR shows high accuracy (**Fig. 3**).  Multi-class classifiers based on non microbially-contaminated data processed with Voom-SNM shows high accuracy (**Supplementary Fig. 9**) | This analysis was performed after filtering to microbes present in WIS analysis (**Fig. 3; Supplementary Fig. 9**). |
